# Competition-based, quantitative chemical proteomics in breast cancer cells identifies new target profiles for sulforaphane†
†Electronic supplementary information (ESI) available: ProteomeXchange with identifier PXD006279. See DOI: 10.1039/c6cc08797c


**DOI:** 10.1039/c6cc08797c

**Published:** 2017-04-25

**Authors:** James A. Clulow, Elisabeth M. Storck, Thomas Lanyon-Hogg, Karunakaran A. Kalesh, Lyn H. Jones, Edward W. Tate

**Affiliations:** a Department of Chemistry , Imperial College London , London , UK SW7 2AZ . Email: e.tate@imperial.ac.uk; b Medicine Design , Pfizer , 610 Main Street , Cambridge , MA 02139 , USA

## Abstract

Sulforaphane is a small molecule isothiocyanate which exhibits anticancer potential, yet its biological targets remain poorly understood. Here we employ a competition-based chemical proteomics strategy to profile sulforaphane's targets and identify over 500 targets along with their relative affinities. These targets provide a new set of mediators for sulforaphane's bioactivity, and aid understanding of its complex mode of action.

## Abstract 2

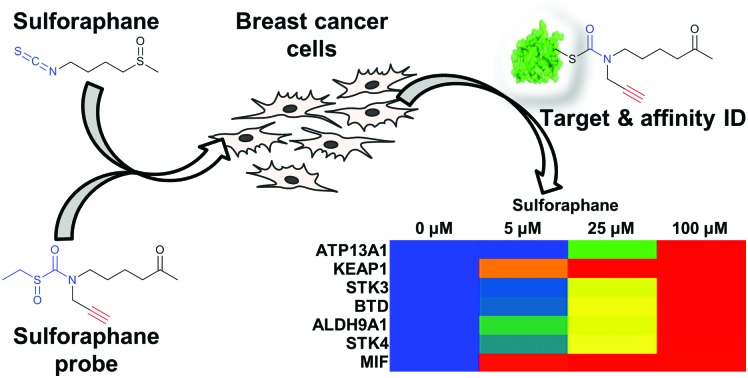
Protein targets of sulforaphane identified, and their affinities quantified, through competition-based chemical proteomics in two live breast cancer cell lines.

## 

Isothiocyanates are a class of bioactive, electrophilic compounds that are produced from metabolism of glucosinolates found in many cruciferous vegetables. Promising *in vitro* cell culture and *in vivo* animal studies for this compound class have led to intense research interest.^1^ One such isothiocyanate, (–)-1-isothiocyanato-(4*R*)-(methylsulfinyl)-butane (sulforaphane), has been particularly well studied owing to its potential anti-cancer activity. Sulforaphane has been suggested to prevent or suppress the development of many types of cancer, including breast cancer;^2^,^3^ its properties have been explored in over 1500 publications and many registered clinical trials, including ongoing trials of a stabilised sulforaphane–cyclodextrin complex in breast cancer. Sulforaphane can covalently modify proteins, as well as other biomolecules, and is widely appreciated to be a polypharmacological agent that likely affects multiple targets and signalling cascades.^4^,^5^ A small number of well-characterised protein targets have been identified for sulforaphane that provide an initial basis for explaining its cellular activities;^6^ however, despite growing interest in sulforaphane and its potential therapeutic application, its molecular targets and underlying mode of action remain poorly characterised.

Understanding the full protein target spectrum of sulforaphane could provide greater insight into its mode of action. The effect of sulforaphane treatment on cellular protein levels has previously been analysed by proteomic strategies;^7^–^9^ however, investigations into alterations in protein expression and/or degradation do not provide insight into direct target binding. Attempts to profile the covalent target spectrum of sulforaphane have employed sulforaphane probes labelled with radioisotopes or bioorthogonal alkyne reporters.^8^,^10^ Analysis of radiolabelled targets by 2D PAGE followed by proteomic identification has a strong bias towards highly abundant targets. In contrast, advances in the use of ‘click’ chemistry functionalisation of bioorthogonal reporters allow for a range of analysis, including global proteomic profiling.^11^ To date, however, a comprehensive and unbiased screen of sulforaphane's targets in a relevant cancer system is still lacking. We therefore sought to employ a quantitative, competition-based chemical proteomics strategy to profile sulforaphane's targets in two breast cancer cell lines, and to identify the relative affinities of these targets for sulforaphane.

Ahn *et al.* previously reported the synthesis and application of cell-permeable alkyne-tagged sulforaphane probe **1** (Fig. 1a);^10^ the electrophilic isothiocyanate group is replaced with sulfoxythiocarbamate, as the product of thiol addition to the latter is more stable. Sulforaphane's sulfoxide moiety can be replaced with a ketone without affecting activity.^10^**1** was reported to identify over 100 protein targets in HEK293 cells by mass spectrometry (MS); however, only two targets of this probe have been validated as genuine sulforaphane targets (macrophage migration inhibitory factor (MIF) and AKAP149), and profiling was not performed in cancer cell lines.^10^ We therefore synthesised **1** alongside a novel probe **2** (Fig. 1a) with greatly reduced steric encumbrance around the electrophilic warhead. **2** was synthesised using a modified procedure to introduce the alkyne handle in a short synthetic sequence from propargylamine (Schemes S1 and S2, ESI†). Sulforaphane and **2** display similar lipophilicity (clog *P* = 0.15 and –0.62, respectively), which is significantly lower than probe **1** (clog *P* = 2.7). This improvement in probe lipophilicity (Δclog *P* = 3.3) was expected to impart reduced non-specific binding to **2**. Probe **1** has already been shown to exert similar biological effects to sulforaphane,^10^ suggesting **2** would also mimic this biological activity. MCF7 and MDA-MB-231 breast cancer cell lines were selected as biologically relevant systems for treatment with **1** and **2**. Following in-cell labelling with each probe, cells were lysed and probe-labelled proteomes functionalised with azido-TAMRA capture reagent^12^ to specifically visualise covalent targets, using well-established copper-catalysed alkyne–azide cycloaddition (CuAAC) methodology.^13^ Proteomes were resolved by SDS-PAGE and analysed by in-gel fluorescence (Fig. 1). To determine the extent to which sulforaphane shares the same target profile as each probe, competition assays were performed whereby cells were incubated with sulforaphane prior to probe treatment. Sulforaphane concentration-dependent reduction in labelling by **1** and **2** was observed for the majority of bands, suggesting a high degree of target overlap (Fig. 1). Competition assays against other electrophilic alkylating agents (iodoacetamide and *N*-ethylmaleimide) indicated common target reactivity with the probes. The sulforaphane-mimetic probe **2** showed the most robust competition by sulforaphane (Fig. 1).

**Fig. 1:**
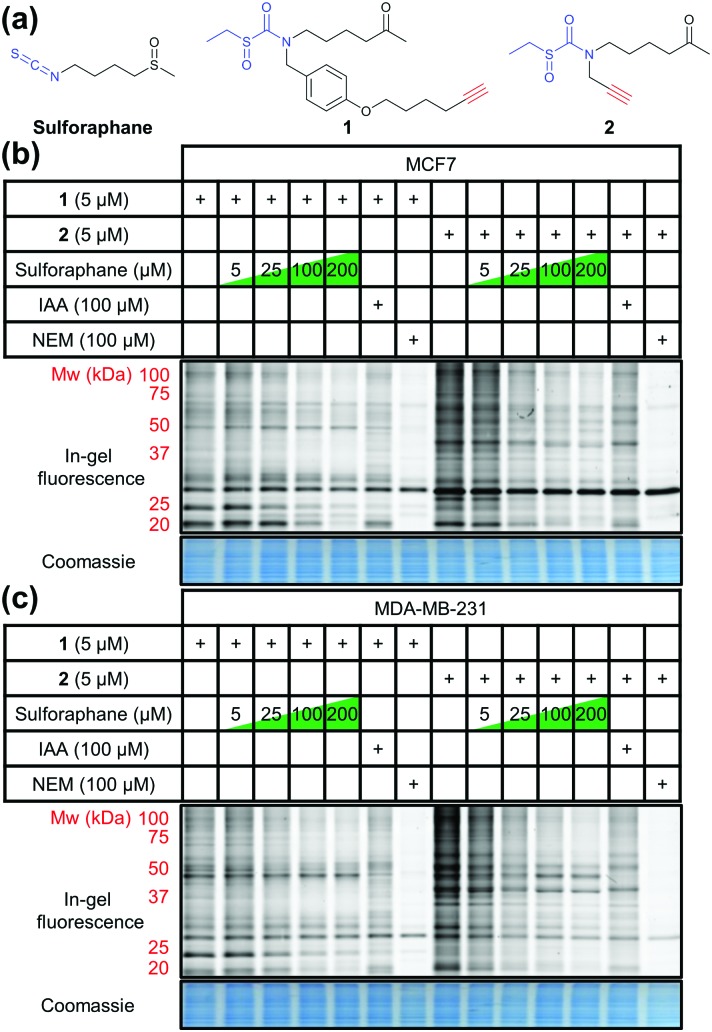
(a) Structures of sulforaphane and probes **1** and **2**. The electrophilic moiety of each molecule is highlighted in blue, and the bioorthogonal alkyne handle in red. (b) MCF7 and (c) MDA-MB-231 lysate in-gel fluorescence and coomassie staining after in-cell labelling with **1** or **2**, competed against a concentration gradient of sulforaphane, iodoacetamide (IAA), or *N*-ethylmaleimide (NEM).

To identify the protein targets of sulforaphane we employed ‘spike-in’ stable isotopic labelling of amino acids in cell culture (SILAC) methodology coupled with competition-based chemical proteomics.^14^ Sulforaphane exerts biological activity at low μM concentrations in MCF7 and MDA-MB-231 cell lines,^15^ therefore cells were cultured under normal conditions and treated with **2** (5 μM) alone, or **2** (5 μM) plus sulforaphane (5, 25 or 100 μM) for 30 min. In parallel, the cell line of interest was labelled with DMEM media containing ^15^N_4_^13^C_6_-arginine and ^15^N_2_^13^C_6_-lysine (R10K8) over multiple passages until incorporation of R10K8 was >98% (Tables S1 and S2, ESI†). R10K8-labelled cells were treated with **2** (20 μM) for 30 min, and lysed to generate a ‘spike-in’ SILAC lysate. A fixed amount of this ‘spike-in’ lysate was added to each competition lysate and the mixed lysates functionalised by CuAAC with an azido-biotin capture reagent.^16^ Labelled proteins were affinity enriched on neutravidin-sepharose, reduced, alkylated and trypsin digested, and the resulting peptides analysed by LC-MS/MS to identify protein targets and quantify enrichment levels across the sulforaphane concentration gradient, using the ‘spike-in’ SILAC peptide as an internal standard to generate heavy/light (H/L) ratios (Fig. 2a). Medium-confidence targets were defined as those giving a statistically significant (*t*-test, S0 = 1, FDR = 0.01) increase in H/L ratio compared to samples treated with **2** only (Fig. S1–S3, ESI†). High-confidence targets were defined as medium-confidence targets present at 5, 25 and 100 μM, or 25 and 100 μM sulforaphane competition (Fig. S4, ESI†). This identified 121 and 129 high-confidence targets in MDA-MB-231 and MCF7 cells, respectively (Fig. 2b), with 56 conserved targets (Fig. 2c and Tables S3 and S4, ESI†). Analysis of published target abundances indicates that the difference in target profile between cell lines may relate to differences in expression levels of target proteins (Fig. S5, ESI†).

**Fig. 2:**
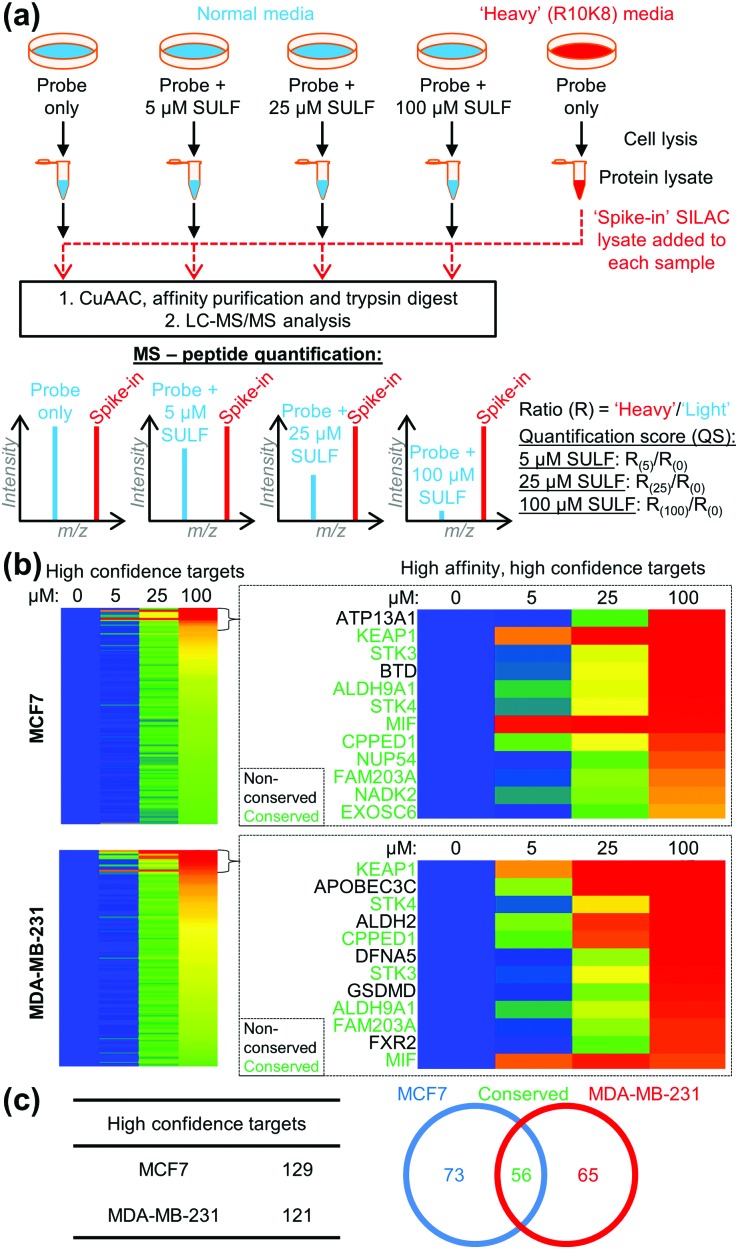
(a) ‘Spike-in’ SILAC workflow. **2** in competition with a sulforaphane (SULF) concentration gradient was incubated with cells grown in normal media, and R10K8 labelled cells were treated with **2** only. Cells were lysed, and ‘spike-in’ R10K8 lysate labelled with **2** added to light lysates. Probe-bound proteins were functionalised by CuAAC, affinity enriched and digested for LC-MS/MS. Heavy/light ratios (*R*_(0)_–*R*_(100)_) were calculated and quantification scores (QS) generated as a ratio of ratios to the probe only sample. (b) Heat maps showing log_2_(QS) over four sulforaphane concentrations (0, 5, 25 and 100 μM). Blue = no competition, red = high competition. (c) Distribution of unique and common high-confidence targets.

The most potent sulforaphane binders were identified through analysis of relative amounts of target captured by **2** over three sulforaphane concentrations (Fig. 2b). Targets exhibiting strong competition for probe labelling at the lowest sulforaphane concentration were hypothesised to be the most potent binders (Fig. 2b). Across both cell lines these included Kelch-like ECH-associated protein 1 (KEAP1) and MIF. KEAP1 is a repressor of the nuclear factor (erythroid-derived 2)-like 2 (Nrf2) transcription factor controlling antioxidant response element (ARE)-driven gene expression,^17^ whilst MIF is a pro-inflammatory cytokine, whose expression in cancer correlates with tumour aggressiveness and metastatic potential.^18^ Both proteins have been previously identified as sulforaphane targets;^17^,^18^ however, this is the first time their interaction with sulforaphane has been observed in a cellular environment at endogenous expression levels. Identification of known sulforaphane targets demonstrates the validity of our approach, and provides the first assessment of the binding of sulforaphane towards these two targets relative to other proteins in the cell, in the absence of artificial overexpression. Sulforaphane is often used as a chemical probe inhibitor of KEAP1; however, our results indicate very substantial target promiscuity at higher concentrations.

Sulforaphane's targets were globally assessed using the bioinformatic platform Ingenuity® Pathway Analysis. The major canonical pathway upregulated in a dose-dependent manner in both cell lines was apoptosis signalling (Fig. S6, ESI†), consistent with sulforaphane's ability to induce apoptosis in numerous cancer cell lines at elevated concentrations.^19^ In both cell lines a common apoptosis signalling target was NF-κB subunits (Fig. S7, ESI†). NF-κB is a ubiquitous transcription factor controlling gene expression of a variety of pro-inflammatory mediators, and activation is linked to cancer cell survival.^20^ Proposed mechanisms for sulforaphane inhibition of NF-κB include interference with an activating kinase IκBα,^21^ reduced NF-κB upstream signalling,^22^ or direct interaction with NF-κB p50 subunit.^23^ Our data shows sulforaphane binds two other NF-κB subunits p65 (RELA, high-confidence in both lines) and p52 (NFKB2, medium- and high-confidence in MCF7 and MDA-MB-231, respectively). Other conserved apoptosis signalling targets include DFFA, PLCG1, and RPS6KA1; apoptosis signalling targets such as BID and ROCK1 were only identified in one cell line (Fig. 3a). Sulforaphane mediates apoptosis in a cell line-specific manner through different apoptotic pathways;^19^ these differences in target profile between MCF7 and MDA-MB-231 may explain such effects.

**Fig. 3:**
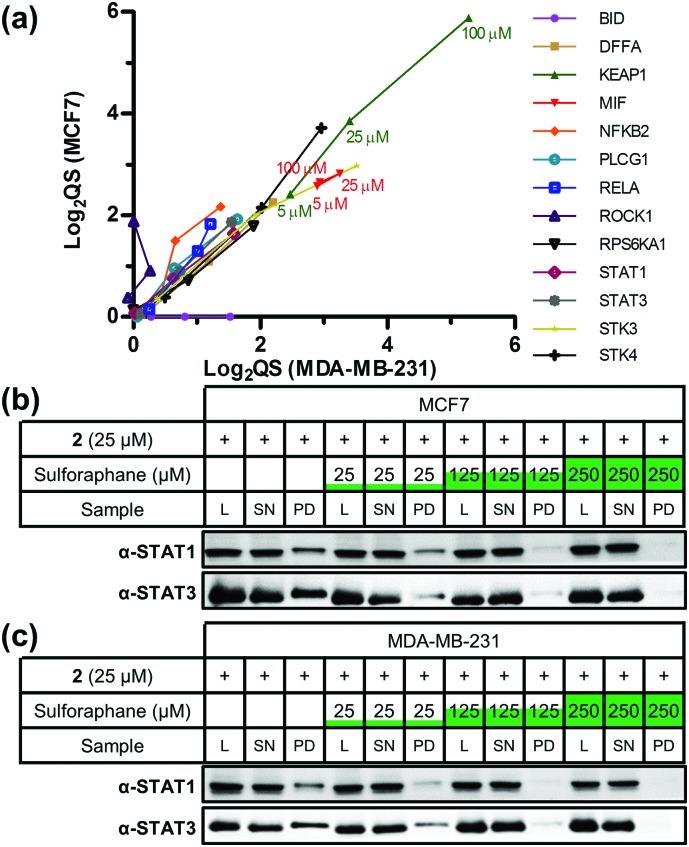
(a) Scatter plot of log_2_(QS) for selected targets across three concentrations of sulforaphane competition (5, 25, and 100 μM). Targets were given a value of 0 when absent from a cell line. (b) MCF7 and (c) MDA-MB-231 western blot analysis showing STAT1 and STAT3 are targets of **2** and sulforaphane labelling competition and pull-down. L = lysate, SN = supernatant, PD = pull-down.

Major downregulated canonical pathways included growth hormone and ERK/MAP kinase signalling pathways (Fig. S6, ESI†), which have been identified as targeted by sulforaphane.^24^,^25^ Several downregulated pathways are mediated *via* the signal transducer and activator of transcription (STAT) 1 and 3 proteins (Fig. S8, ESI†). STAT3 was a high-confidence target in both lines, and STAT1 a high-confidence and medium-confidence target in MCF7 and MDA-MB-231, respectively. STAT proteins are latent cytosolic transcription factors; STAT3 in particular is an oncogenic transcription factor constitutively expressed in a variety of cancers, resulting in expression of various genes involved in cell proliferation.^26^ STAT1 and STAT3 were therefore validated as sulforaphane targets *via* labelling with **2** in competition with sulforaphane, followed by CuAAC functionalisation, pull-down and western blot analysis (Fig. 3b, c and Fig. S9, ESI†). Sulforaphane inhibition of STAT3 signalling has been reported previously, with the proposed mechanism of action through reduction in protein level and phosphorylation status of STAT3's activator kinase, JAK2.^27^ Our data show sulforaphane also directly binds STAT1 and STAT3.

Disease and biofunction analysis of sulforaphane's targets indicated upregulation of organismal death (40 targets in MCF7 and 34 in MDA-MB-231) and downregulation of cell proliferation (58 targets in MCF7) and cell viability (24 targets in MDA-MB-231) with sulforaphane treatment (Fig. S10–S12, ESI†). Common in these functions were high-affinity, high-confidence conserved targets serine/threonine kinase (STK) 3 and 4, which function in the Hippo pathway to regulate cell proliferation and apoptosis.^28^

The conserved target profile between cell lines similarly affected apoptotic and growth signalling, with corresponding upregulation of cell death biofunctions (Fig. S13, ESI†). Analysis of the interaction network of conserved targets highlighted a high degree of connectivity around Akt protein kinases and caspases (Fig. S14, ESI†). Although neither are identified as a direct target of sulforaphane in our study, previous studies have shown sulforaphane can downregulate total and phosphorylated Akt levels leading to antiproliferative effects,^29^ and promote activation of various caspases in apoptosis.^19^ Our target profile therefore identifies several potential mediators of these effects.

The presented dataset represents the most comprehensive direct target profile of sulforaphane to date, and highlights the wide range of targets that sulforaphane covalently binds. It should, however, be noted that use of sulfoxythiocarbamate warheads in electrophilic probes may affect target reactivity or target labelling dependent on transient metabolic modification, for example by glutathionylation. Sulforaphane induces varied biological effects at different concentrations,^30^ and our quantitative data provides insight into how such effects could be mediated by demonstrating targets that engage sulforaphane at different therapeutically relevant concentrations. These target profiles are orthogonal datasets that complement general proteomic and transcriptomic studies of sulforaphane treatment,^8^ and will provide a valuable starting point for future studies.

Target profile differences between the two cell lines highlight the potential for sulforaphane to differentiate between breast cancers based on molecular mechanism. The use of cleavable capture reagents may also allow identification of sulforaphane binding sites in future. Increased understanding of sulforaphane's targets and underlying mode of action may ultimately provide better insight into how best to apply this agent or related electrophilic compounds in the clinic.

This work was supported by the UK Engineering and Physical Sciences Research Council and Pfizer (EPSRC Industrial CASE Studentship award to J. A. C.). E. M. S. acknowledges the award of a PhD studentship from the British Heart Foundation. T. L.-H. was supported by Cancer Research UK (C29637/A20781). K. A. K. was funded by the European Commission's Research Executive Agency (Marie Curie International Incoming Fellowship, FP7-PEOPLE-2011-IIF). L. H. J. is an employee and shareholder of Pfizer.

## Supplementary Material

Supplementary information

Supplementary information

Supplementary information
