# Supplementary material for: Competition-based, quantitative chemical proteomics in breast cancer cells identifies new target profiles for sulforaphane
Source: Chem Commun (Camb). 2017 Apr 25;53(37):5182–5. doi: 10.1039/c6cc08797c (PMC6034444; doi:10.1039/c6cc08797c)
Supplement: Supplementary file 1 [file CC-053-C6CC08797C-s001.pdf]

Electronic Supplementary Material (ESI) for Chemical Communications.
This journal is © The Royal Society of Chemistry 2017

Supplementary Information for

Competition-based, quantitative chemical proteomics in breast cancer cells identifies new target profiles for sulforaphane

James A. Clulow,[a,‡] Elisabeth M. Storck,[a,‡] Thomas Lanyon-Hogg,[a,‡] Karunakaran A. Kalesh,[a] Lyn Jones[b] and Edward W. Tate[a,*]

[a] Department of Chemistry, Imperial College London, London, UK, SW7 2AZ
[b] Worldwide Medicinal Chemistry, Pfizer, 200 Cambridge Park Drive, MA 02140, USA
‡ Authors share equal contribution
* corresponding author, email: e.tate@imperial.ac.uk

Table of Contents

1 Supporting Figures 3

2 Supporting Tables 17

2.1 Supporting Table S1. Incorporation validation for the R10K8 label in the 'spike-in' SILAC proteome of the MCF7 cell line 17

2.2 Supporting Table S2. Incorporation validation for the R10K8 label in the 'spike-in' SILAC proteome of the MDA-MB-231 cell line 17

2.3 Supporting Table S3. High- and medium- confidence targets of sulforaphane in the MCF7 cell line 17

2.4 Supporting Table S4. High- and medium- confidence targets of sulforaphane in the MDA-MB-231 cell line 24

3 Chemical synthesis methods 36

3.1 General Information 36

3.2 Synthesis of Probe 1 36

3.2.1 4-Hex-5-ynyloxy-benzaldehyde 37

3.2.2 2-(4-Chloro-butyl)-2-methyl-[1,3]dioxolane 37

3.2.3 2-(4-Phthalimido-butyl)-2-methyl-[1,3]dioxolane 37

3.2.4 2-(4-Amino-butyl)-2-methyl-[1,3]dioxolane 38

3.2.5 (4-hex-5-ynyloxy-benzyl)-[4-(2-methyl-[1,3]dioxolan-2-yl)-butyl]-amine 38

3.2.6 *S*-Ethyl (4-hex-5-ynyloxy-benzyl)-[4-(2-methyl-[1,3]dioxolan-2-yl)-butyl]-thiocarbamate 38

3.2.7 *S*-Ethyl (4-hex-5-ynyloxy-benzyl)-(5-oxo-hexyl)-thiocarbamate 39

3.2.8 *S*-Ethyl(4-hex-5-ynyloxy-benzyl)-(5-oxo-hexyl)-thiocarbamatesulfoxide (1) 39

3.3 Synthesis of Probe 2 40

3.3.1 *S*-Ethyl (prop-2-ynyl)-[4-(2-methyl-[1,3]dioxolan-2-yl)-butyl]-thiocarbamate 40

3.3.2 *S*-Ethyl (prop-2-ynyl)-(5-oxo-hexyl)-thiocarbamate 41

3.3.3 *S*-Ethyl (prop-2-ynyl)-(5-oxo-hexyl)-thiocarbamate sulfoxide (2) 41

3.4 ^{1}H NMR, ^{13}C NMR, HRMS and LC-MS data 42

4 Biological and biochemical methods 46

4.1 General methods 46

4.2 Cell culture 46

4.3 In-cell competition-based assays for in-gel fluorescence and Western Blot analyses 46

4.3.1 Compound treatment of cells and cell lysis 46

4.3.2 Copper catalysed azide-alkyne cycloaddition (CuAAC) 47

4.3.3 Gel electrophoresis and in-gel fluorescence 47

4.3.4 Affinity enrichment and Western Blot analysis 47
4.4 In-cell quantitative, competition-based assays for target identification by mass spectrometry 48
4.4.1 Compound treatment of cells and cell lysis 48
4.4.2 Preparation of ‘spike-in’ SILAC cell lysates as quantification standards 49
4.4.3 CuAAC, affinity enrichment and on-bead reduction, alkylation and trypsin digest 49
4.4.4 LC-MS/MS 50
4.4.5 LC-MS/MS data analysis 50
4.4.6 Abundance and bioinformatic analysis 51
5 References 52

1 Supporting Figures

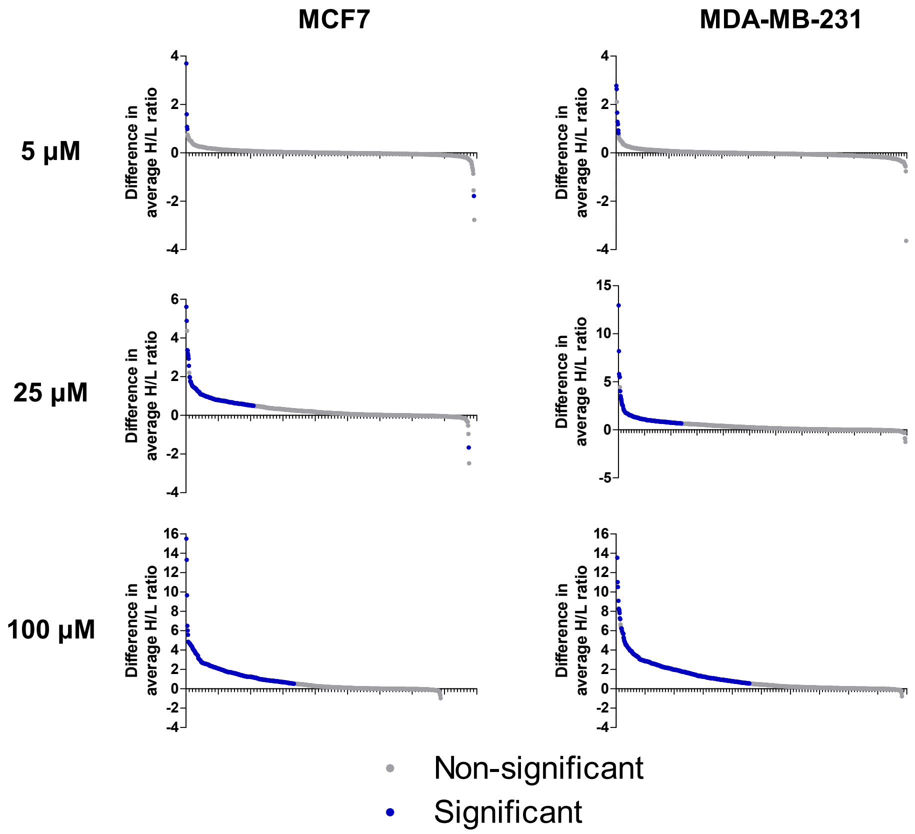

Supporting Figure S1. Target profiles of **2** in competition with three concentrations of sulforaphane in MCF7 and MDA-MB-231 cells. Potential targets were identified as giving a statistically significant change (t-test, S0 = 1, FDR = 0.01, shown in blue) in H/L ratio compared to samples treated with **2** alone.

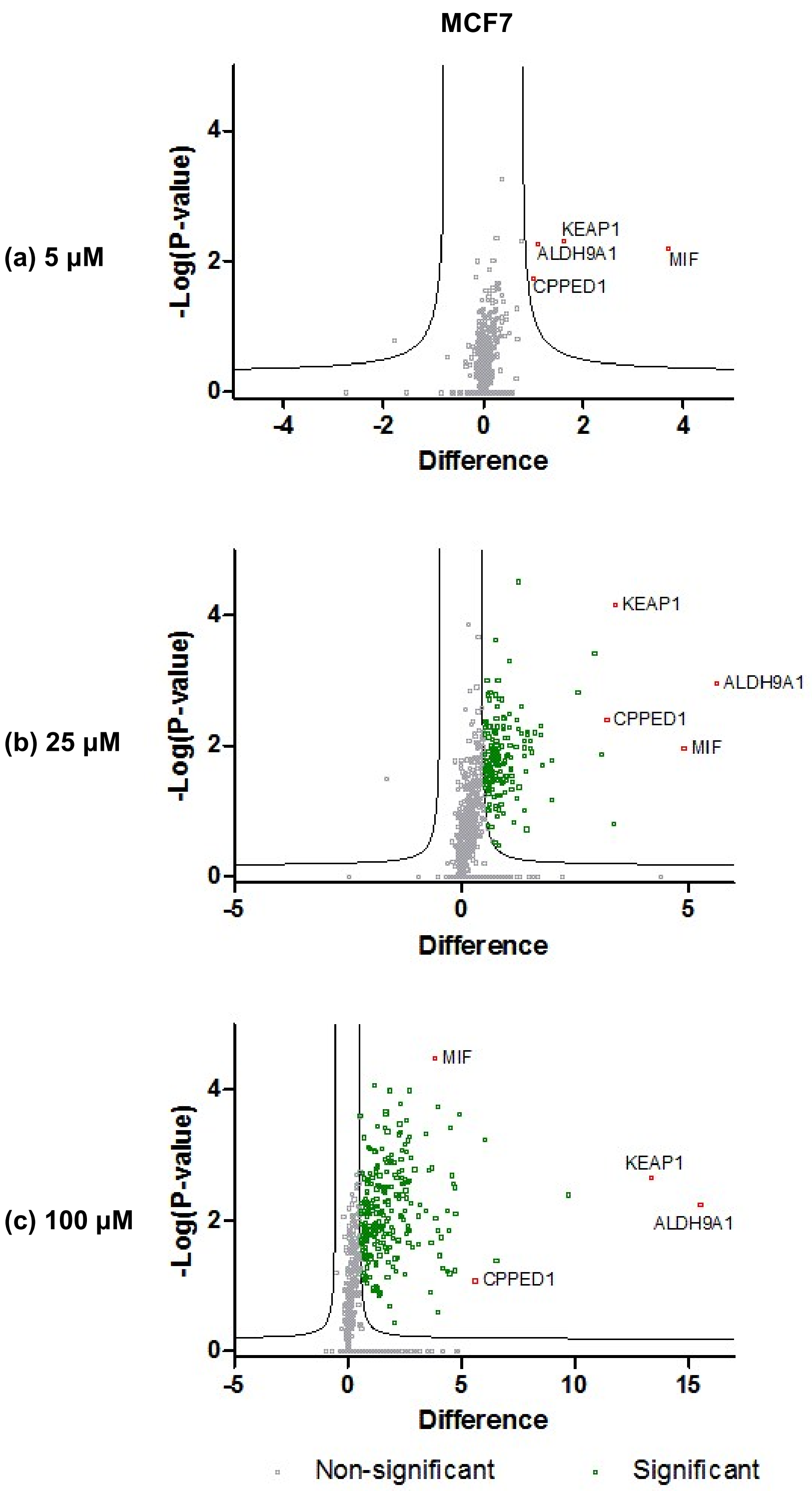

Supporting Figure S2. Volcano plots for **2** in competition with three concentrations of sulforaphane in MCF7. Medium-confidence targets were identified as giving a statistically significant change (t-test, S0 = 1, FDR = 0.01, shown in green) in H/L ratio compared to samples treated with **2** alone. The four high affinity and conserved targets MIF, KEAP1, ALDH9A1 and CPPED1 are highlighted to show distribution at each concentration.

MDA-MB-231

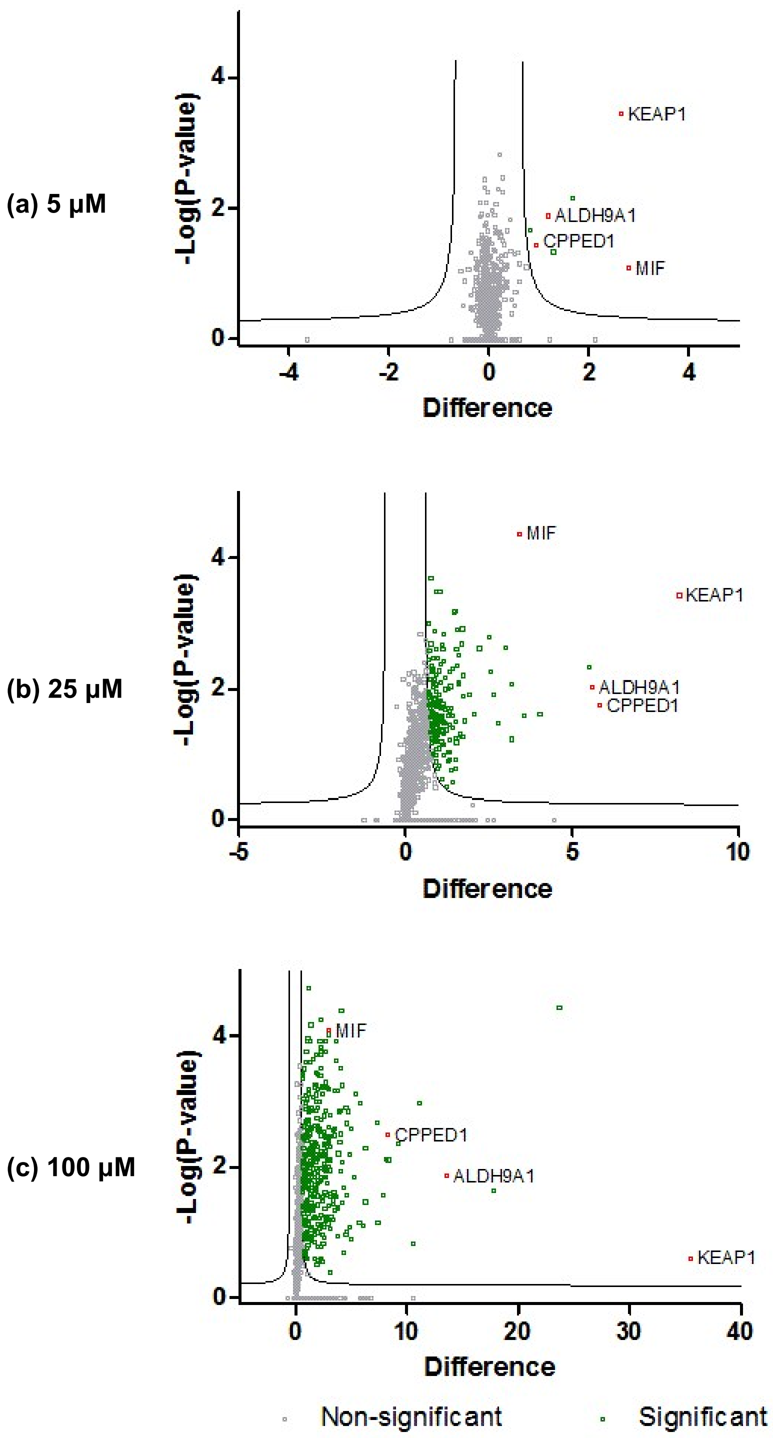

Supporting Figure S3. Volcano plots for **2** in competition with three concentrations of sulforaphane in MDA-MB-231 cells. Medium-confidence targets were identified as giving a statistically significant change (t-test, S0 = 1, FDR = 0.01, shown in green) in H/L ratio compared to samples treated with **2** alone. The four high affinity and conserved targets MIF, KEAP1, ALDH9A1 and CPPED1 are highlighted to show distribution at each concentration.

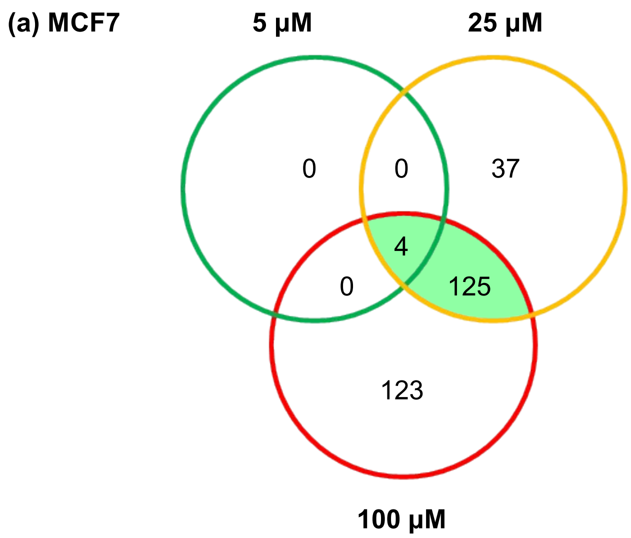

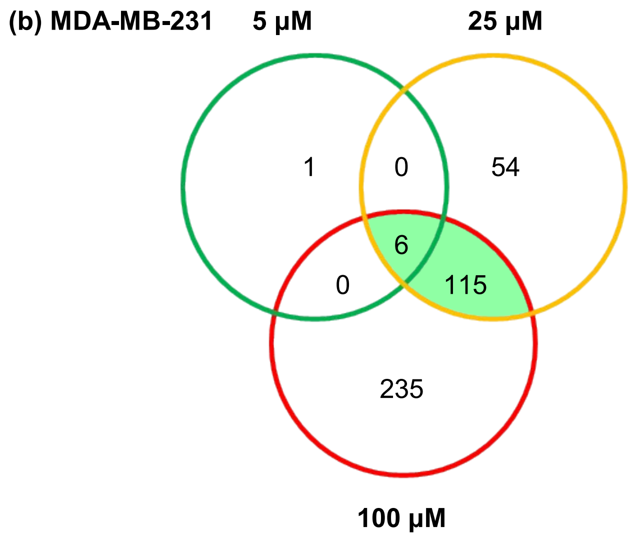

Supporting Figure S4. Medium- and high-confidence targets of sulforaphane in (a) MCF7 and (b) MDA-MB-231 cells. Medium-confidence targets were identified as giving a statistically significant (t-test, S0 = 1, FDR = 0.01) change in H/L ratio at one sulforaphane concentration. Medium-confidence targets identified at 5, 25, and 100 μM, or at 25 and 100 μM, were defined as high-confidence targets (highlighted green).

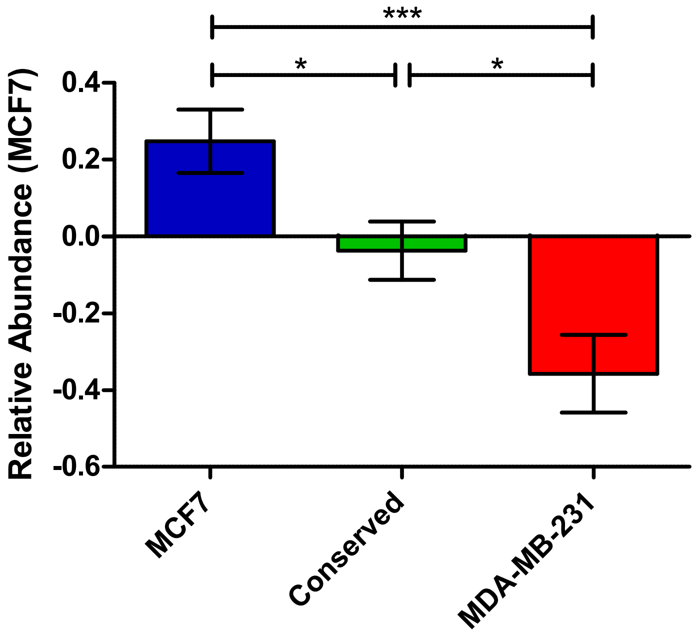

Supporting Figure S5. Comparions of difference in relative abundance levels for conserved and cell line-specific high-confidence targets of sulforaphane. Relative abundance was calculated as described in materials and methods using intensity based absolute quantification (iBAQ) values for each cell line from proteomic analysis of the NCI-60 cell line panel.[1] A relative abundance > 0 indicates a protein is present at higher levels in MCF7 cells than in MDA-MB-231 cells; conversely a relative abundance < 0 indicates a protein is present at higher levels in MDA-MB-231 cells than in MCF7 cells. Average relative abundance of targets indicates that cell line-specific targets show higher abundance within that cell line (* = $p < 0.05$, *** = $p < 0.0001$).

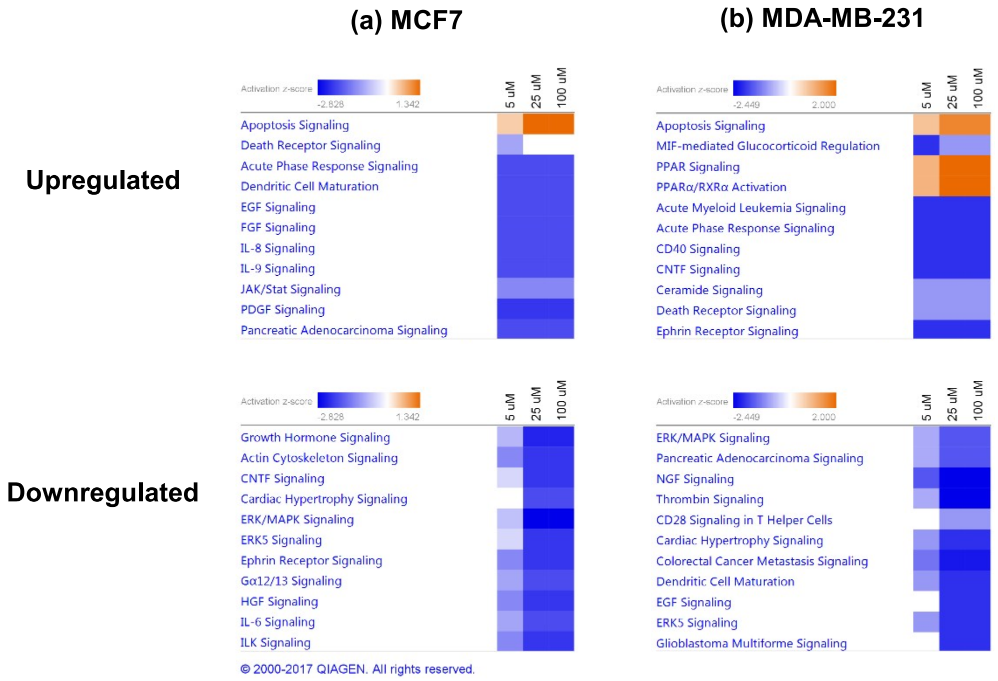

Supporting Figure S6. IPA heatmap analysis of up- and downregulated canonical pathways in (a) MCF7 and (b) MDA-MB-231 cell lines. Heatmaps are ordered by increasing or decreasing z-score trend, and coloured with orange indicating upregulation and blue indicating downregulation.

(a) Sulforaphane targets in apoptosis signalling in MCF7:

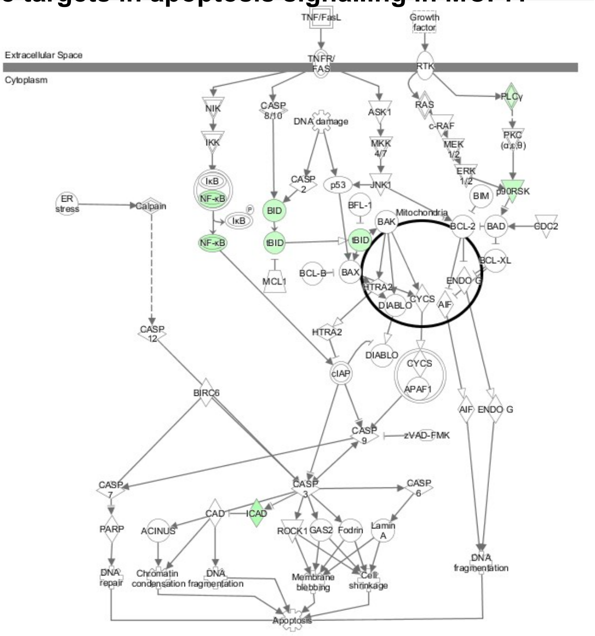

(b) Sulforaphane targets in apoptosis signalling in MDA-MB-231:

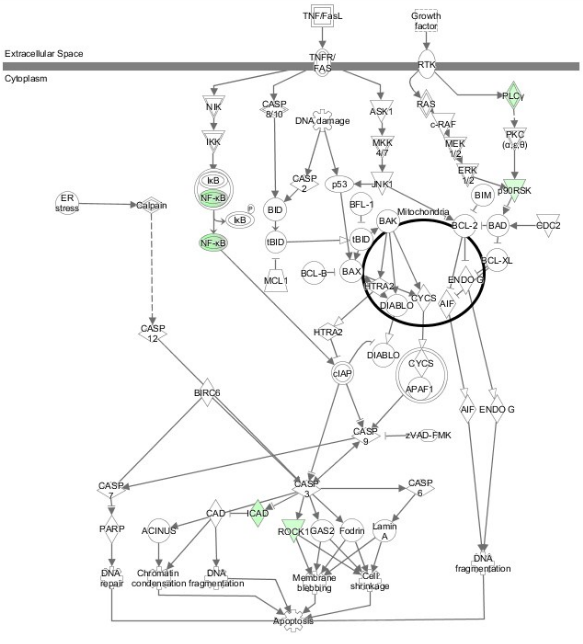

© 2000-2017 QIAGEN. All rights reserved.

Supporting Figure S7. Targets in upregulated apoptosis signalling in (a) MCF7 and (b) MDA-MB-231 cell lines. Protein targets of sulforpahane are highlighted in green with colour density representing $-\log_2$(QS) at 25 µM, with denser colour indicating lower $-\log_2$(QS). NF-κB subunits are common in both cell lines, along with PLCG1, p90RSK (RPS6KA1), and ICAD (DFFA).

(a) Sulforaphane targets in growth hormone signalling in MCF7:

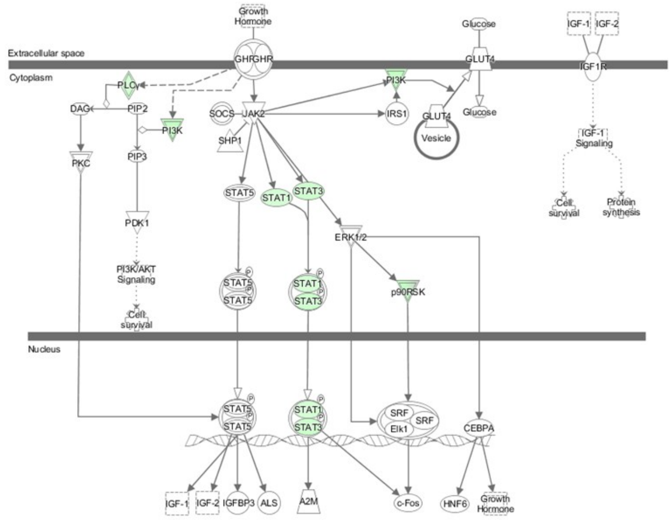

(b) Sulforaphane targets in ERK/MAPK signalling in MDA-MB-231:

Supporting Figure S8. Major downregulated signalling pathways in (a) MCF7 and (b) MDA-MB-231 cell lines. Protein targets of sulforaphane are highlighted in green with colour density representing $–log_2$(QS) at 25 μM, with denser colour indicating lower $–log_2$(QS). STAT transcription factors are common to both predominantly downregulated pathways.

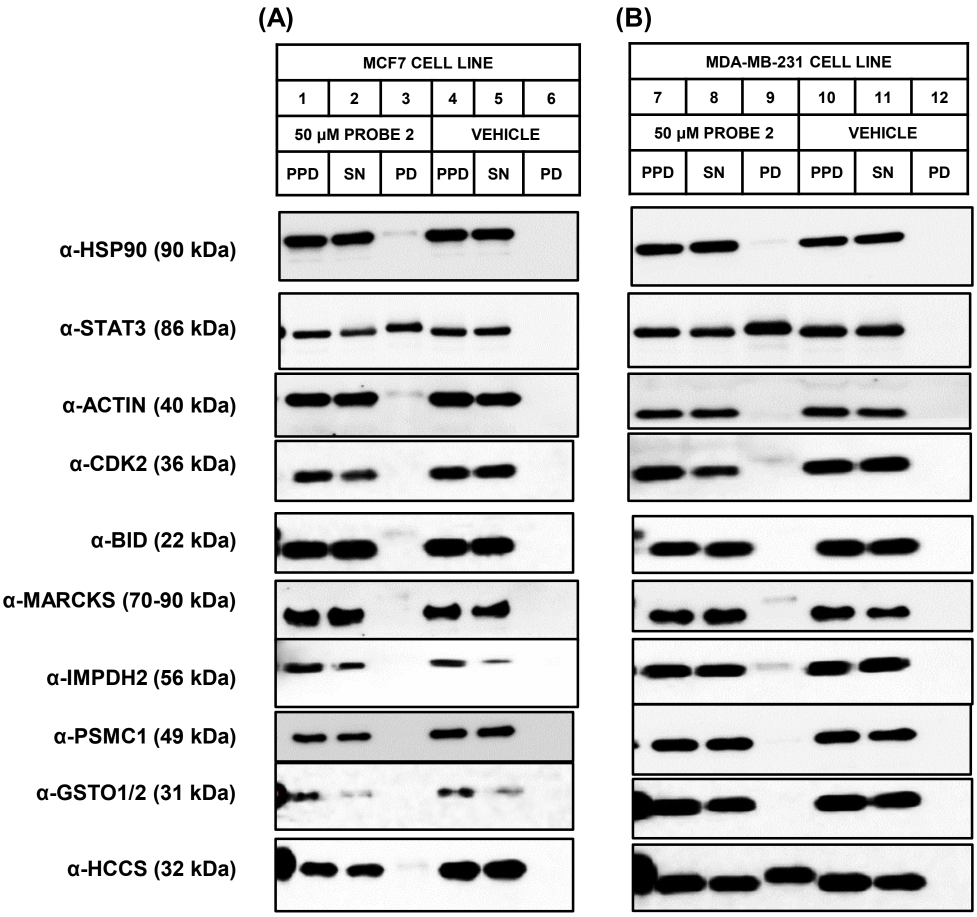

Supporting Figure S9. Western Blot analyses of potential targets (HSP90, STAT3, ACTIN, CDK2, BID, MARCKS, IMPDH2, PSMC1, GSTO1/2 and HCCS) of **2** (50 µM) in MCF7 (a) and MDA-MB-231 (b) cell lines compared to vehicle (DMSO) treatment. PPD = pre-pull down lysate, SN = supernatant lysate after pulldown and PD = pulldown.

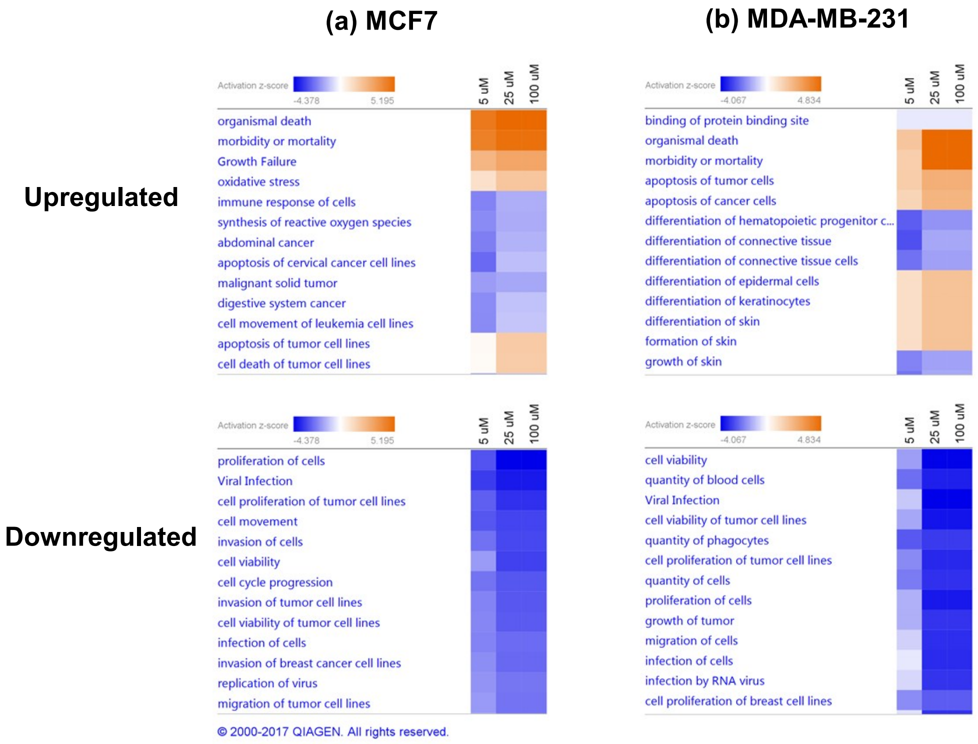

Supporting Figure S10. IPA heatmap analysis of up- and downregulated disease and biofunctions in (a) MCF7 and (b) MDA-MB-231 cell lines. Heatmaps are ordered by increasing or decreasing z-score trend and z-score, and coloured with orange indicating upregulation and blue indicating downregulation.

MCF7

(a) Organismal death | (b) Proliferation of cells

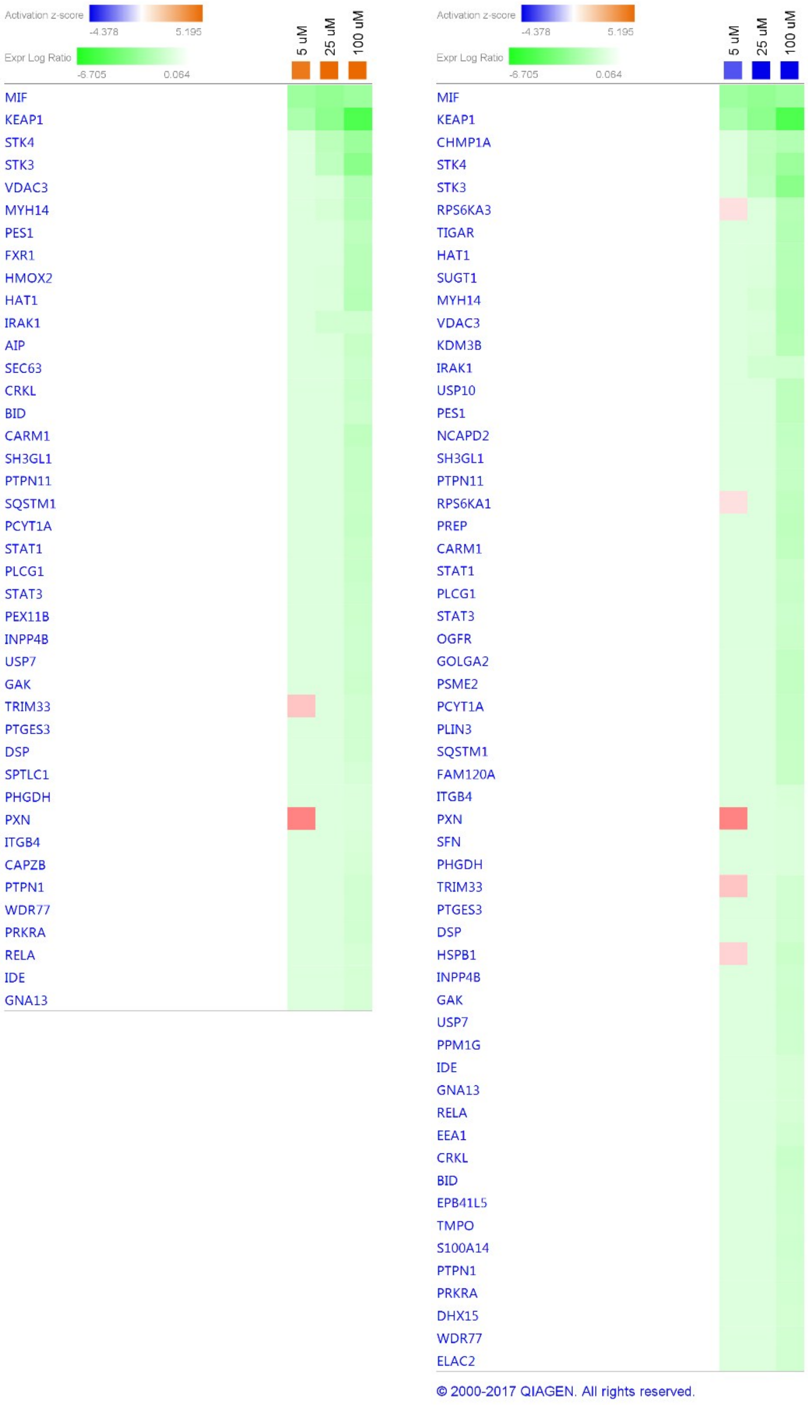

Supporting Figure S11. Heatmap of high confidence sulforaphane targets involved in disease and biofunctions in MCF7 cells. (a) Targets involved in upregulation of organismal death, and (b) targets involved in downregulation of proliferation of cells. Heatmap density represents $-\log_2(QS)$ at each concentration.

MDA-MB-231

(a) Organismal death (b) Cell viability

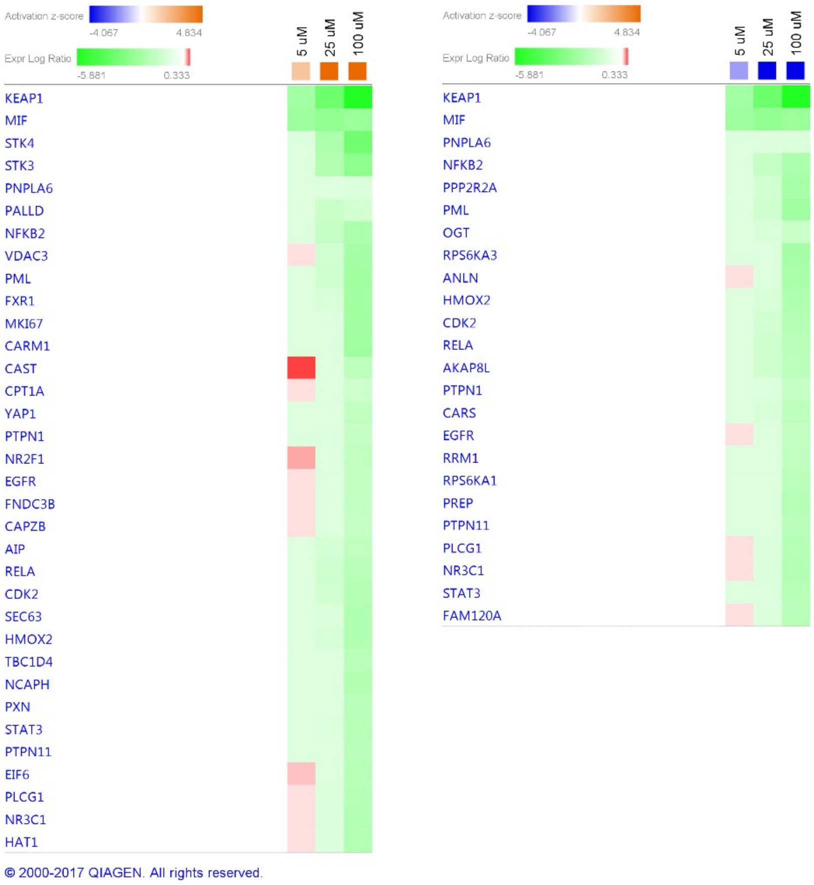

Supporting Figure S12. Heatmap of high confidence sulforaphane targets involved in disease and biofunctions in MDA-MB-231 cells. (a) Targets involved in upregulation of organismal death, and (b) targets involved in downregulation of cell viability. Heatmap density represents $-\log_2$(QS) at each concentration. Comparison with MCF7 cells (Figure S11) indicates several targets present in multiple functions, including KEAP1, MIF, STAT3, STK3, STK4, and RELA.

Conserved Targets

(a) Canonical Pathways

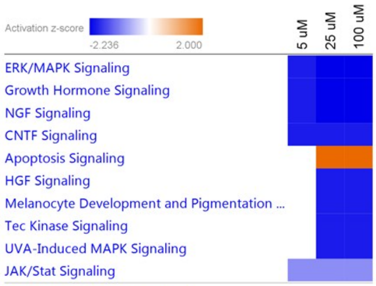

(b) Diseases and Biofunctions

Upregulated

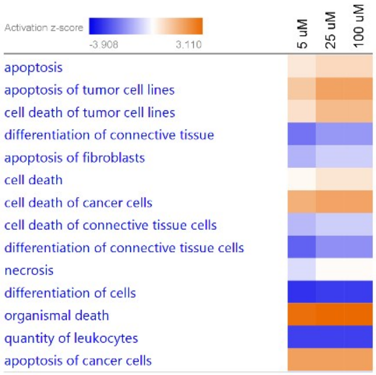

Downregulated

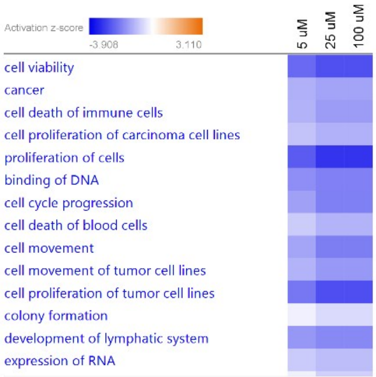

© 2000-2017 QIAGEN. All rights reserved.

Supporting Figure S13. Analysis of conserved targets of sulforaphane in MCF7 and MD-MBA-231 cells. (a) All canonical pathways modulated by sulforaphane. (b) Upregulated and down regulated diseases and biofunctions. Heatmap is ordered by increasing or decreasing z-score trend, and coloured with orange indicating upregulation and blue indicating downregulation.

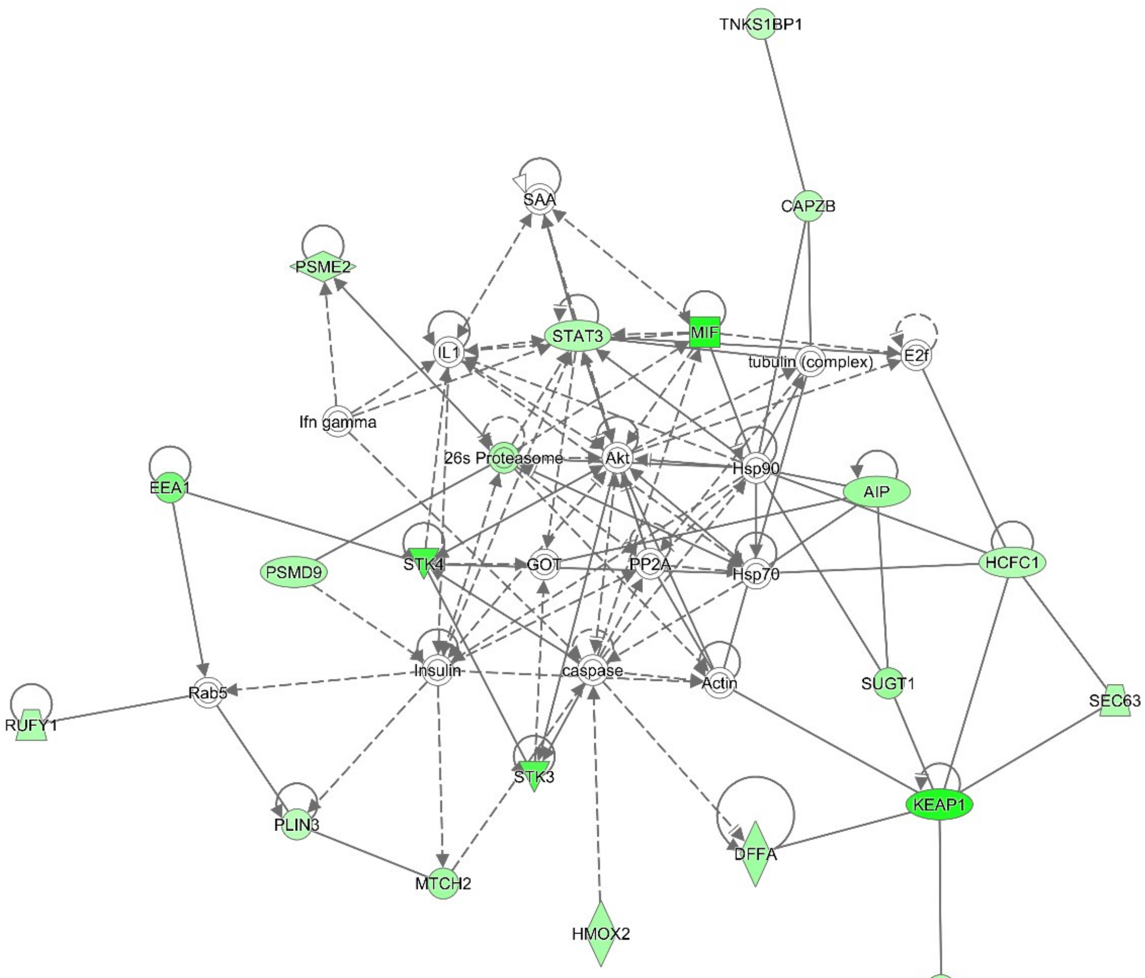

© 2000-2017 QIAGEN. All rights reserved.

Supporting Figure S14. Network analysis of conserved targets of sulforaphane in MCF7 and MD-MBA-231 cell lines at 25 μM, indicating a high-degree of connectivity around Akt kinases and caspases.

2 Supporting Tables

2.1 Supporting Table S1. Incorporation validation for the R10K8 label in the 'spike-in' SILAC proteome of the MCF7 cell line

See additional Excel file.

2.2 Supporting Table S2. Incorporation validation for the R10K8 label in the 'spike-in' SILAC proteome of the MDA-MB-231 cell line

See additional Excel file.

2.3 Supporting Table S3. High- and medium- confidence targets of sulforaphane in the MCF7 cell line

	Gene names	Protein names	Confidence	Conserved	Log_2QS		
					Sulforaphane (µM)		
					5	25	100
1	**ABHD12**	Monoacylglycerol lipase ABHD12	High	N	0.23	0.83	1.40
2	**ACAD9**	Acyl-CoA dehydrogenase family member 9, mitochondrial	High	Y	0.31	0.85	1.31
3	**AIP**	AH receptor-interacting protein;Peptidyl-prolyl cis-trans isomerase	High	Y	0.29	1.04	1.68
4	**AKAP1**	A-kinase anchor protein 1, mitochondrial	High	Y	0.08	0.74	1.75
5	**ALDH6A1**	Methylmalonate-semialdehyde dehydrogenase [acylating], mitochondrial	High	N	0.16	1.07	1.75
6	**ALDH9A1**	4-trimethylaminobutyraldehyde dehydrogenase	High	Y	0.63	1.95	3.15
7	**ASMTL**	N-acetylserotonin O-methyltransferase-like protein	High	N	0.13	0.80	1.61
8	**ATG4B**	Cysteine protease ATG4B	High	N	0.00	0.50	1.29
9	**ATP13A1**	Probable cation-transporting ATPase 13A1	High	N	0.04	0.82	6.70
10	**ATP6V1A**	V-type proton ATPase catalytic subunit A	High	Y	0.15	1.00	1.95
11	**BAG5**	BAG family molecular chaperone regulator 5	High	N	0.13	0.91	1.89
12	**BID**	BH3-interacting domain death agonist;BH3-interacting domain death agonist p15;BH3-interacting domain death agonist p13;BH3-interacting domain death agonist p11	High	N	0.28	0.81	1.52
13	**BOLA2;BOLA2B;LOC101060252**	BolA-like protein 2	High	N	0.22	1.10	2.27
14	**BTD**	Biotinidase	High	Y	0.40	2.00	3.51
15	**CAD**	CAD protein;Glutamine-dependent carbamoyl-phosphate synthase;Aspartate carbamoyltransferase;Dihydroorotase	High	Y	0.18	0.80	1.53
16	**CAPZB**	F-actin-capping protein subunit beta	High	Y	0.01	0.65	1.21
17	**CARM1**	Histone-arginine methyltransferase CARM1	High	Y	0.09	0.80	1.87
18	**CHMP1A**	Charged multivesicular body protein 1a	High	N	0.72	1.95	2.27
19	**CKAP4**	Cytoskeleton-associated protein 4	High	Y	0.06	0.65	1.27
20	**CPPED1**	Calcineurin-like phosphoesterase domain-containing protein 1	High	Y	0.97	2.04	2.69
21	**CRKL**	Crk-like protein	High	Y	0.33	0.76	1.60
22	**DFFA**	DNA fragmentation factor subunit alpha	High	Y	0.26	1.18	2.19
23	**DHX15**	Putative pre-mRNA-splicing factor ATP-dependent RNA helicase DHX15	High	Y	0.09	0.77	1.31
24	**DNPEP**	Aspartyl aminopeptidase	High	Y	0.06	0.64	1.74
25	**DSP**	Desmoplakin	High	N	0.09	0.63	1.37
26	**DUT**	Deoxyuridine 5-triphosphate nucleotidohydrolase, mitochondrial	High	Y	0.14	0.68	1.57
27	**DYNLL1;DYNLL2**	Dynein light chain 1, cytoplasmic;Dynein light chain 2, cytoplasmic	High	Y	0.11	0.62	1.94
28	**EEA1**	Early endosome antigen 1	High	Y	0.29	0.95	1.35

	Gene names	Protein names	Confidence	Conserved	Log_2QS		
					Sulforaphane (μM)		
					5	25	100
29	**ELAC2**	Zinc phosphodiesterase ELAC protein 2	High	Y	0.13	0.71	1.36
30	**EPB41L5**	Band 4.1-like protein 5	High	N	0.12	0.93	1.45
31	**ESYT1**	Extended synaptotagmin-1	High	Y	0.09	0.65	1.33
32	**EXOSC6**	Exosome complex component MTR3	High	Y	0.10	0.87	2.31
33	**FAM120A**	Constitutive coactivator of PPAR-gamma-like protein 1	High	Y	0.08	0.78	1.68
34	**FAM203A**	Protein FAM203A	High	Y	0.35	1.53	2.46
35	**FAM83H**	Protein FAM83H	High	N	0.00	0.83	1.54
36	**FXR1**	Fragile X mental retardation syndrome-related protein 1	High	Y	0.06	0.91	2.10
37	**GAK**	Cyclin-G-associated kinase	High	N	0.03	0.56	1.52
38	**GEMIN5**	Gem-associated protein 5	High	Y	0.01	0.60	1.43
39	**GLOD4**	Glyoxalase domain-containing protein 4	High	Y	0.07	0.67	1.70
40	**GNA13**	Guanine nucleotide-binding protein subunit alpha-13	High	N	0.14	0.93	1.20
41	**GOLGA2**	Golgin subfamily A member 2	High	Y	0.04	0.64	1.76
42	**GREB1**	Protein GREB1	High	N	0.09	0.75	2.27
43	**GSR**	Glutathione reductase, mitochondrial	High	N	0.17	0.84	1.56
44	**GSTM3**	Glutathione S-transferase Mu 3	High	Y	-0.01	0.48	0.94
45	**HAT1**	Histone acetyltransferase type B catalytic subunit	High	Y	0.12	1.03	2.17
46	**HCFC1**	Host cell factor 1;HCF N-terminal chain 1;HCF N-terminal chain 2;HCF N-terminal chain 3;HCF N-terminal chain 4;HCF N-terminal chain 5;HCF N-terminal chain 6;HCF C-terminal chain 1;HCF C-terminal chain 2;HCF C-terminal chain 3;HCF C-terminal chain 4;HCF C-terminal chain 5;HCF C-terminal chain 6	High	Y	0.05	0.68	1.77
47	**HDHD3**	Haloacid dehalogenase-like hydrolase domain-containing protein 3	High	N	0.37	1.44	1.58
48	**HMOX2**	Heme oxygenase 2	High	Y	0.21	1.11	2.09
49	**HSPB1**	Heat shock protein beta-1	High	Y	-0.01	0.50	1.58
50	**IDE**	Insulin-degrading enzyme	High	N	0.09	0.89	1.21
51	**INPP4B**	Type II inositol 3,4-bisphosphate 4-phosphatase	High	N	0.05	0.47	1.45
52	**IPO5**	Importin-5	High	Y	0.13	0.62	1.02
53	**IRAK1**	Interleukin-1 receptor-associated kinase 1	High	N	0.35	1.38	1.40
54	**ITGB4**	Integrin beta-4	High	N	0.08	0.60	1.17
55	**ITPK1**	Inositol-tetrakisphosphate 1-kinase	High	N	-0.02	0.76	2.11
56	**KDM3B**	Lysine-specific demethylase 3B	High	Y	0.26	1.12	2.11
57	**KEAP1**	Kelch-like ECH-associated protein 1	High	Y	2.47	3.40	5.28
58	**KHSRP**	Far upstream element-binding protein 2	High	Y	0.20	0.78	1.35
59	**LANCL2**	LanC-like protein 2	High	Y	0.24	0.88	1.92
60	**LPCAT1**	Lysophosphatidylcholine acyltransferase 1	High	N	0.12	0.60	1.44
61	**LPP**	Lipoma-preferred partner	High	N	NaN	0.57	0.94
62	**MCMBP**	Mini-chromosome maintenance complex-binding protein	High	Y	0.07	0.73	2.09
63	**MIF**	Macrophage migration inhibitory factor	High	Y	2.89	3.24	2.93
64	**MMS19**	MMS19 nucleotide excision repair protein homolog	High	Y	0.09	0.72	1.73
65	**MRPL39**	39S ribosomal protein L39, mitochondrial	High	Y	0.36	0.89	1.54
66	**MTCH2**	Mitochondrial carrier homolog 2	High	Y	0.43	1.14	1.66
67	**MTHFD1L**	Monofunctional C1-tetrahydrofolate synthase, mitochondrial	High	Y	0.03	0.71	1.53

	Gene names	Protein names	Confidence	Conserved	Log$_2$QS		
					Sulforaphane (μM)		
					5	25	100
68	**MYH14**	Myosin-14	High	N	0.22	1.22	2.28
69	**NADK2**	NAD kinase 2, mitochondrial	High	Y	0.52	1.43	2.39
70	**NCAPD2**	Condensin complex subunit 1	High	Y	0.11	1.01	1.80
71	**NPEPL1**	Probable aminopeptidase NPEPL1	High	N	0.27	1.24	2.29
72	**NT5DC1**	5-nucleotidase domain-containing protein 1	High	N	0.23	0.96	1.67
73	**NUDCD1**	NudC domain-containing protein 1	High	Y	0.07	0.71	1.59
74	**NUP155**	Nuclear pore complex protein Nup155	High	Y	0.06	0.86	1.57
75	**NUP54**	Nucleoporin p54	High	Y	0.03	0.96	2.58
76	**OGFR**	Opioid growth factor receptor	High	Y	0.04	0.74	1.58
77	**PCYT1A; PCYT1B**	Choline-phosphate cytidylyltransferase A;Choline-phosphate cytidylyltransferase B	High	Y	0.02	0.79	1.74
78	**PES1**	Pescadillo homolog	High	N	0.25	0.99	1.95
79	**PEX11B**	Peroxisomal membrane protein 11B	High	N	0.07	0.78	1.50
80	**PFAS**	Phosphoribosylformylglycinamidine synthase	High	Y	-0.01	0.63	1.06
81	**PGLS**	6-phosphogluconolactonase	High	Y	0.07	1.04	1.78
82	**PHGDH**	D-3-phosphoglycerate dehydrogenase	High	N	0.08	0.48	1.08
83	**PLCG1**	1-phosphatidylinositol 4,5-bisphosphate phosphodiesterase gamma-1	High	Y	0.05	0.64	1.63
84	**PLIN3**	Perilipin-3	High	Y	0.06	0.75	1.72
85	**PPM1G**	Protein phosphatase 1G	High	Y	0.03	0.57	1.43
86	**PREP**	Prolyl endopeptidase	High	Y	0.11	0.76	1.94
87	**PRKRA**	Interferon-inducible double stranded RNA-dependent protein kinase activator A	High	N	0.20	0.78	1.33
88	**PRMT3**	Protein arginine N-methyltransferase 3	High	Y	0.11	0.84	1.56
89	**PSMD9**	26S proteasome non-ATPase regulatory subunit 9	High	Y	0.04	0.80	2.21
90	**PSME2**	Proteasome activator complex subunit 2	High	Y	0.10	0.81	1.75
91	**PTGES3**	Prostaglandin E synthase 3	High	Y	0.09	0.62	1.36
92	**PTPN1**	Tyrosine-protein phosphatase non-receptor type 1;Tyrosine-protein phosphatase non-receptor type	High	Y	0.16	0.85	1.36
93	**PTPN11**	Tyrosine-protein phosphatase non-receptor type 11	High	Y	0.16	0.87	1.72
94	**PUS1**	tRNA pseudouridine synthase;tRNA pseudouridine synthase A, mitochondrial	High	N	0.11	0.80	1.30
95	**PXN**	Paxillin	High	Y	-0.04	0.59	1.09
96	**RANBP6**	Ran-binding protein 6	High	Y	0.39	1.24	2.11
97	**RELA**	Transcription factor p65	High	Y	0.25	1.01	1.21
98	**RNF114**	RING finger protein 114	High	N	0.21	0.84	1.50
99	**RNPEP**	Aminopeptidase B	High	Y	0.14	0.74	1.25
100	**RPS6KA1**	Ribosomal protein S6 kinase alpha-1;Ribosomal protein S6 kinase	High	Y	-0.01	0.86	1.88
101	**RPS6KA3**	Ribosomal protein S6 kinase alpha-3	High	Y	0.00	0.57	2.14
102	**RTFDC1**	Protein RTF2 homolog	High	N	-0.02	0.57	1.57
103	**RTN3**	Reticulon-3	High	Y	0.17	0.99	1.50
104	**RUFY1**	RUN and FYVE domain-containing protein 1	High	Y	0.02	0.84	1.37
105	**S100A14**	Protein S100-A14	High	N	0.20	0.65	1.47
106	**SEC63**	Translocation protein SEC63 homolog	High	Y	0.20	0.89	1.55
107	**SFN**	14-3-3 protein sigma	High	Y	0.04	0.54	1.09
108	**SH3GL1**	Endophilin-A2	High	N	0.18	0.93	1.72

	Gene names	Protein names	Confidence	Conserved	Log$_2$QS		
					Sulforaphane (μM)		
					5	25	100
109	**SPTLC1**	Serine palmitoyltransferase 1	High	N	0.21	0.64	1.18
110	**SQSTM1**	Sequestosome-1	High	Y	0.06	0.80	1.67
111	**STAT1**	Signal transducer and activator of transcription 1-alpha/beta	High	Y	0.02	0.63	1.58
112	**STAT3**	Signal transducer and activator of transcription 3	High	Y	0.12	0.74	1.54
113	**STK3**	Serine/threonine-protein kinase 3;Serine/threonine-protein kinase 3 36kDa subunit;Serine/threonine-protein kinase 3 20kDa subunit	High	Y	0.37	1.91	3.52
114	**STK4**	Serine/threonine-protein kinase 4;Serine/threonine-protein kinase 4 37kDa subunit;Serine/threonine-protein kinase 4 18kDa subunit	High	Y	0.49	2.02	2.96
115	**SUGT1**	Suppressor of G2 allele of SKP1 homolog	High	Y	0.44	1.07	2.15
116	**TBC1D15**	TBC1 domain family member 15	High	Y	0.02	0.70	1.41
117	**TCF25**	Transcription factor 25	High	N	0.19	0.74	1.54
118	**THNSL1**	Threonine synthase-like 1	High	Y	0.14	0.98	1.92
119	**TIGAR**	Fructose-2,6-bisphosphatase TIGAR	High	Y	0.10	0.88	2.23
120	**TMPO**	Lamina-associated polypeptide 2, isoform alpha;Thymopoietin;Thymopentin	High	Y	0.27	0.74	1.40
121	**TNKS1BP1**	182 kDa tankyrase-1-binding protein	High	Y	-0.01	0.56	1.40
122	**TRIM33**	E3 ubiquitin-protein ligase TRIM33	High	N	-0.02	0.62	1.32
123	**TXLNG**	Gamma-taxilin	High	Y	-0.06	0.70	1.56
124	**USP10**	Ubiquitin carboxyl-terminal hydrolase 10	High	Y	0.25	0.97	1.95
125	**USP7**	Ubiquitin carboxyl-terminal hydrolase 7;Ubiquitin carboxyl-terminal hydrolase	High	Y	0.04	0.57	1.45
126	**VDAC2**	Voltage-dependent anion-selective channel protein 2	High	Y	0.20	0.87	1.63
127	**VDAC3**	Voltage-dependent anion-selective channel protein 3	High	Y	0.27	1.09	2.24
128	**WDR77**	Methylosome protein 50	High	Y	0.14	0.75	1.33
129	**XPO5**	Exportin-5	High	Y	0.10	0.61	1.42
130	**ADAR**	Double-stranded RNA-specific adenosine deaminase	Medium	Y	0.18	0.53	1.14
131	**ADRM1**	Proteasomal ubiquitin receptor ADRM1	Medium	Y	0.08	0.71	1.50
132	**AGAP3**	Arf-GAP with GTPase, ANK repeat and PH domain-containing protein 3	Medium	N	-0.20	0.58	NaN
133	**AHCY**	Adenosylhomocysteinase	Medium	Y	-0.01	0.41	1.03
134	**AHNAK**	Neuroblast differentiation-associated protein AHNAK	Medium	N	-0.08	0.46	0.87
135	**AIM1**	Absent in melanoma 1 protein	Medium	N	0.30	1.16	2.26
136	**AIMP2**	Aminoacyl tRNA synthase complex-interacting multifunctional protein 2	Medium	Y	0.04	0.54	1.33
137	**ANKHD1; ANKRD17**	Ankyrin repeat and KH domain-containing protein 1;Ankyrin repeat domain-containing protein 17	Medium	N	-0.21	0.45	1.14
138	**AP4E1**	AP-4 complex subunit epsilon-1	Medium	N	0.18	1.01	-0.44
139	**ARAF**	Serine/threonine-protein kinase A-Raf	Medium	N	0.11	0.74	1.30
140	**ASNS**	Asparagine synthetase [glutamine-hydrolyzing]	Medium	Y	0.03	0.39	1.17
141	**ASPSCR1**	Tether containing UBX domain for GLUT4	Medium	N	0.22	1.16	NaN
142	**ATXN2L**	Ataxin-2-like protein	Medium	Y	0.04	0.50	1.29
143	**BAG3**	BAG family molecular chaperone regulator 3	Medium	Y	0.00	0.48	1.37
144	**BAG6**	Large proline-rich protein BAG6	Medium	Y	0.08	0.35	0.97
145	**BAX**	Apoptosis regulator BAX	Medium	N	-0.01	0.57	1.51
146	**BRAT1**	BRCA1-associated ATM activator 1	Medium	N	0.02	0.29	1.25

	Gene names	Protein names	Confidence	Conserved	Log$_2$QS		
					Sulforaphane (μM)		
					5	25	100
147	**CARS**	Cysteine--tRNA ligase, cytoplasmic	Medium	Y	0.15	0.87	1.88
148	**CASP2**	Caspase-2;Caspase-2 subunit p18;Caspase-2 subunit p13;Caspase-2 subunit p12	Medium	N	-0.11	0.72	2.10
149	**CCDC6**	Coiled-coil domain-containing protein 6	Medium	N	0.12	0.74	1.46
150	**CDK2**	Cyclin-dependent kinase 2	Medium	N	0.22	0.77	1.22
151	**CLN3**	Battenin	Medium	N	0.29	0.88	1.79
152	**CPT1A**	Carnitine O-palmitoyltransferase 1, liver isoform	Medium	Y	0.08	0.52	1.43
153	**CTSB**	Cathepsin B;Cathepsin B light chain;Cathepsin B heavy chain	Medium	Y	0.29	0.74	1.13
154	**CTSC**	Dipeptidyl peptidase 1;Dipeptidyl peptidase 1 exclusion domain chain;Dipeptidyl peptidase 1 heavy chain;Dipeptidyl peptidase 1 light chain	Medium	Y	0.10	0.88	NaN
155	**CTTN**	Src substrate cortactin	Medium	Y	0.02	0.28	0.99
156	**CYFIP1**	Cytoplasmic FMR1-interacting protein 1	Medium	N	0.14	0.60	1.24
157	**DCTPP1**	dCTP pyrophosphatase 1	Medium	N	0.21	0.13	0.81
158	**DDX3X;DDX3Y**	ATP-dependent RNA helicase DDX3X;ATP-dependent RNA helicase DDX3Y	Medium	N	-0.02	0.33	0.81
159	**DIAPH1**	Protein diaphanous homolog 1	Medium	Y	0.08	0.45	0.96
160	**DNM1L**	Dynamin-1-like protein	Medium	Y	-0.15	0.21	0.83
161	**DSTN**	Destrin	Medium	Y	0.00	0.31	0.95
162	**EDC4**	Enhancer of mRNA-decapping protein 4	Medium	Y	0.00	0.42	0.92
163	**EIF3G**	Eukaryotic translation initiation factor 3 subunit G	Medium	Y	-0.19	0.30	0.74
164	**EPRS**	Bifunctional glutamate/proline--tRNA ligase;Glutamate--tRNA ligase;Proline--tRNA ligase	Medium	Y	-0.03	0.30	0.91
165	**EPS8L2**	Epidermal growth factor receptor kinase substrate 8-like protein 2	Medium	Y	-0.05	0.49	1.26
166	**FASN**	Fatty acid synthase;[Acyl-carrier-protein] S-acetyltransferase;[Acyl-carrier-protein] S-malonyltransferase;3-oxoacyl-[acyl-carrier-protein] synthase;3-oxoacyl-[acyl-carrier-protein] reductase;3-hydroxyacyl-[acyl-carrier-protein] dehydratase;Enoyl-[acyl-carrier-protein] reductase;Oleoyl-[acyl-carrier-protein] hydrolase	Medium	Y	0.06	0.50	0.82
167	**FBXO22**	F-box only protein 22	Medium	N	0.05	0.95	NaN
168	**FLII**	Protein flightless-1 homolog	Medium	Y	-0.04	0.30	1.00
169	**FMR1**	Fragile X mental retardation protein 1	Medium	Y	0.00	1.28	2.04
170	**FXR2**	Fragile X mental retardation syndrome-related protein 2	Medium	Y	0.35	1.04	2.07
171	**GARS**	Glycine--tRNA ligase	Medium	Y	0.01	0.45	1.03
172	**GART**	Trifunctional purine biosynthetic protein adenosine-3;Phosphoribosylamine--glycine ligase;Phosphoribosylformylglycinamidine cyclo-ligase;Phosphoribosylglycinamide formyltransferase	Medium	Y	-0.02	0.27	0.67
173	**GCLC**	Glutamate--cysteine ligase catalytic subunit	Medium	N	0.67	1.91	2.10
174	**GFPT1**	Glutamine--fructose-6-phosphate aminotransferase [isomerizing] 1	Medium	Y	0.08	0.66	1.34
175	**GMPPB**	Mannose-1-phosphate guanyltransferase beta	Medium	N	0.14	0.44	1.09
176	**GMPS**	GMP synthase [glutamine-hydrolyzing]	Medium	Y	-0.07	0.39	0.93
177	**GNB2**	Guanine nucleotide-binding protein G(I)/G(S)/G(T) subunit beta-2	Medium	N	0.06	0.43	1.12
178	**GRPEL1**	GrpE protein homolog 1, mitochondrial	Medium	N	-0.03	0.30	0.66
179	**GSPT1**	Eukaryotic peptide chain release factor GTP-binding subunit ERF3A	Medium	N	0.07	0.46	1.17
180	**HCCS**	Cytochrome c-type heme lyase	Medium	Y	0.12	1.00	2.07
181	**HDGF**	Hepatoma-derived growth factor	Medium	Y	0.07	0.36	1.58

	Gene names	Protein names	Confidence	Conserved	Log$_2$QS		
					Sulforaphane (μM)		
					5	25	100
182	**HDLBP**	Vigilin	Medium	N	0.01	0.30	0.71
183	**HEATR6**	HEAT repeat-containing protein 6	Medium	N	-0.02	0.31	0.79
184	**HECTD1**	E3 ubiquitin-protein ligase HECTD1	Medium	Y	-0.11	0.35	1.22
185	**HNRNPF**	Heterogeneous nuclear ribonucleoprotein F;Heterogeneous nuclear ribonucleoprotein F, N-terminally processed	Medium	Y	0.04	0.65	1.34
186	**HNRNPK**	Heterogeneous nuclear ribonucleoprotein K	Medium	N	-0.03	0.37	0.78
187	**HNRNPUL2;hCG_2044799**	Heterogeneous nuclear ribonucleoprotein U-like protein 2	Medium	N	0.15	0.48	1.08
188	**HPRT1**	Hypoxanthine-guanine phosphoribosyltransferase	Medium	Y	-0.05	0.32	1.04
189	**HSD17B10**	3-hydroxyacyl-CoA dehydrogenase type-2	Medium	Y	0.05	0.47	1.15
190	**HSDL1**	Inactive hydroxysteroid dehydrogenase-like protein 1	Medium	N	0.04	0.48	1.02
191	**HSPBP1**	Hsp70-binding protein 1	Medium	Y	0.01	0.26	2.03
192	**HSPH1**	Heat shock protein 105 kDa	Medium	Y	-0.02	0.20	0.70
193	**HUWE1**	E3 ubiquitin-protein ligase HUWE1	Medium	Y	0.01	0.43	1.17
194	**IMPDH2**	Inosine-5-monophosphate dehydrogenase 2	Medium	Y	0.08	0.36	0.76
195	**INF2**	Inverted formin-2	Medium	Y	0.07	0.39	0.83
196	**INPPL1**	Phosphatidylinositol 3,4,5-trisphosphate 5-phosphatase 2	Medium	N	0.17	0.70	1.27
197	**ISOC2**	Isochorismatase domain-containing protein 2, mitochondrial	Medium	Y	0.07	0.56	1.77
198	**KIAA1598**	Shootin-1	Medium	N	-0.06	0.45	1.37
199	**KLC1**	Kinesin light chain 1	Medium	Y	0.03	0.52	1.37
200	**KTN1**	Kinectin	Medium	N	0.05	0.37	0.93
201	**LACTB**	Serine beta-lactamase-like protein LACTB, mitochondrial	Medium	Y	0.35	1.04	1.66
202	**LARP1**	La-related protein 1	Medium	Y	0.05	0.52	1.03
203	**LARP4**	La-related protein 4	Medium	Y	-0.16	0.14	1.01
204	**LARP4B**	La-related protein 4B	Medium	Y	0.09	0.41	1.00
205	**LCMT1**	Leucine carboxyl methyltransferase 1	Medium	Y	0.09	1.09	NaN
206	**LGALS1**	Galectin-1	Medium	Y	0.02	0.40	1.00
207	**LRBA**	Lipopolysaccharide-responsive and beige-like anchor protein	Medium	N	-0.09	0.25	0.90
208	**MACROD1**	O-acetyl-ADP-ribose deacetylase MACROD1	Medium	N	-0.06	0.70	1.29
209	**MAVS**	Mitochondrial antiviral-signaling protein	Medium	Y	0.06	0.54	1.33
210	**MCCC2**	Methylcrotonoyl-CoA carboxylase beta chain, mitochondrial	Medium	N	-0.01	0.34	0.83
211	**MCM3**	DNA replication licensing factor MCM3	Medium	Y	0.01	0.39	1.14
212	**MEPCE**	7SK snRNA methylphosphate capping enzyme	Medium	N	-0.11	0.49	1.68
213	**MTA2**	Metastasis-associated protein MTA2	Medium	Y	0.21	0.83	1.14
214	**MVD**	Diphosphomevalonate decarboxylase	Medium	N	0.12	0.74	1.90
215	**MYH9**	Myosin-9	Medium	Y	-0.05	0.24	0.90
216	**MYL6**	Myosin light polypeptide 6	Medium	Y	-0.09	0.31	1.18
217	**NCAPH**	Condensin complex subunit 2	Medium	Y	0.34	0.63	1.81
218	**NFKB2**	Nuclear factor NF-kappa-B p100 subunit;Nuclear factor NF-kappa-B p52 subunit	Medium	Y	0.44	0.66	1.37
219	**NONO**	Non-POU domain-containing octamer-binding protein	Medium	Y	0.03	0.22	0.87
220	**NR2F2;NR2F1**	COUP transcription factor 2;COUP transcription factor 1	Medium	Y	0.03	0.77	1.34
221	**NUDC**	Nuclear migration protein nudC	Medium	Y	0.00	0.39	1.16

	Gene names	Protein names	Confidence	Conserved	Log_2QS		
					Sulforaphane (μM)		
					5	25	100
222	**OTUB1**	Ubiquitin thioesterase OTUB1	Medium	N	-0.03	0.39	1.37
223	**OTUD6B**	OTU domain-containing protein 6B	Medium	Y	-0.11	0.43	1.47
224	**PAFAH1 B2**	Platelet-activating factor acetylhydrolase IB subunit beta	Medium	N	0.02	0.40	0.78
225	**PCBP1**	Poly(rC)-binding protein 1	Medium	Y	-0.02	0.53	1.57
226	**PCBP2**	Poly(rC)-binding protein 2	Medium	Y	0.11	0.56	1.11
227	**PCMT1**	Protein-L-isoaspartate(D-aspartate) O-methyltransferase;Protein-L-isoaspartate O-methyltransferase	Medium	Y	0.02	0.54	1.54
228	**PDCD6IP**	Programmed cell death 6-interacting protein	Medium	Y	-0.09	0.23	0.86
229	**PDE12**	2,5-phosphodiesterase 12	Medium	Y	0.49	0.74	1.48
230	**PGAM5**	Serine/threonine-protein phosphatase PGAM5, mitochondrial	Medium	N	0.21	0.69	1.10
231	**PGP**	Phosphoglycolate phosphatase	Medium	N	-0.06	0.17	0.74
232	**PGPEP1**	Pyroglutamyl-peptidase 1	Medium	Y	-0.09	0.42	1.48
233	**PML**	Protein PML	Medium	Y	0.24	0.67	0.75
234	**PMPCB**	Mitochondrial-processing peptidase subunit beta	Medium	N	0.20	0.65	1.07
235	**POP1**	Ribonucleases P/MRP protein subunit POP1	Medium	N	0.30	-0.03	1.40
236	**PPME1**	Protein phosphatase methylesterase 1	Medium	Y	-0.07	0.22	0.63
237	**PPP1R3D**	Protein phosphatase 1 regulatory subunit 3D	Medium	N	0.15	0.79	NaN
238	**PPP2R2A**	Serine/threonine-protein phosphatase 2A 55 kDa regulatory subunit B alpha isoform	Medium	Y	-0.01	0.75	1.63
239	**PPP6R3**	Serine/threonine-protein phosphatase 6 regulatory subunit 3	Medium	Y	-0.11	0.22	0.76
240	**PREX1**	Phosphatidylinositol 3,4,5-trisphosphate-dependent Rac exchanger 1 protein	Medium	N	0.04	0.63	1.45
241	**PRKDC**	DNA-dependent protein kinase catalytic subunit	Medium	Y	-0.13	0.56	1.11
242	**PRMT1**	Protein arginine N-methyltransferase 1	Medium	Y	0.01	0.47	1.22
243	**PROSC**	Proline synthase co-transcribed bacterial homolog protein	Medium	N	0.26	1.26	NaN
244	**PRRC2C**	Protein PRRC2C	Medium	Y	-0.06	0.31	0.91
245	**PSME1**	Proteasome activator complex subunit 1	Medium	N	0.04	0.36	0.73
246	**PSMG1**	Proteasome assembly chaperone 1	Medium	N	0.03	0.38	1.02
247	**PYGB**	Glycogen phosphorylase, brain form	Medium	Y	-0.07	0.36	0.73
248	**QARS**	Glutamine--tRNA ligase	Medium	Y	-0.10	0.23	0.90
249	**RABL6**	Rab-like protein 6	Medium	N	-0.07	0.54	1.09
250	**RARS**	Arginine--tRNA ligase, cytoplasmic	Medium	Y	-0.10	0.33	0.96
251	**RBBP7;R BBP4**	Histone-binding protein RBBP7;Histone-binding protein RBBP4	Medium	N	0.04	0.34	0.81
252	**RCC2**	Protein RCC2	Medium	Y	0.09	0.47	0.96
253	**RNF14**	E3 ubiquitin-protein ligase RNF14	Medium	N	0.04	0.67	NaN
254	**RPN1**	Dolichyl-diphosphooligosaccharide--protein glycosyltransferase subunit 1	Medium	N	0.02	0.52	1.02
255	**RPS6KB1**	Ribosomal protein S6 kinase beta-1	Medium	N	0.00	0.14	0.70
256	**RRBP1**	Ribosome-binding protein 1	Medium	Y	0.05	0.46	1.00
257	**RRM1**	Ribonucleoside-diphosphate reductase large subunit	Medium	Y	-0.09	0.36	1.26
258	**SCCPDH**	Saccharopine dehydrogenase-like oxidoreductase	Medium	Y	0.10	1.28	NaN
259	**SEC16A**	Protein transport protein Sec16A	Medium	Y	0.00	0.36	1.03
260	**SEC24C**	Protein transport protein Sec24C	Medium	Y	0.00	0.49	1.33
261	**SLC5A6**	Sodium-dependent multivitamin transporter	Medium	N	0.05	0.57	0.91

	Gene names	Protein names	Confidence	Conserved	Log$_2$QS		
					Sulforaphane (μM)		
					5	25	100
262	**SMC2**	Structural maintenance of chromosomes protein 2	Medium	Y	-0.14	0.64	1.62
263	**SPCS2**	Signal peptidase complex subunit 2	Medium	N	0.18	0.91	1.47
264	**SPTLC2**	Serine palmitoyltransferase 2	Medium	Y	-0.02	0.46	1.21
265	**SYNCRIP**	Heterogeneous nuclear ribonucleoprotein Q	Medium	N	-0.09	0.38	0.95
266	**TACC3**	Transforming acidic coiled-coil-containing protein 3	Medium	Y	-0.23	0.19	1.00
267	**TAGLN2**	Transgelin-2	Medium	Y	0.03	0.40	0.89
268	**TBC1D30**	TBC1 domain family member 30	Medium	N	0.46	0.79	1.59
269	**TBC1D9B**	TBC1 domain family member 9B	Medium	N	0.01	0.94	NaN
270	**TBCD**	Tubulin-specific chaperone D	Medium	Y	0.09	0.38	0.97
271	**TBL2**	Transducin beta-like protein 2	Medium	N	0.15	0.54	1.24
272	**THUMPD1**	THUMP domain-containing protein 1	Medium	N	0.11	0.85	1.38
273	**TPM3;DKFZp686J1372**	Tropomyosin alpha-3 chain	Medium	Y	-0.03	0.29	1.11
274	**TRIM25**	E3 ubiquitin/ISG15 ligase TRIM25	Medium	Y	-0.03	0.46	1.07
275	**TRIP6**	Thyroid receptor-interacting protein 6	Medium	N	-0.05	0.34	1.46
276	**TTLL12**	Tubulin--tyrosine ligase-like protein 12	Medium	N	-0.01	0.24	0.82
277	**TXN**	Thioredoxin	Medium	N	-0.02	0.05	0.74
278	**TXNRD1**	Thioredoxin reductase 1, cytoplasmic	Medium	Y	-0.93	0.05	0.43
279	**UBA6**	Ubiquitin-like modifier-activating enzyme 6	Medium	Y	-0.14	0.25	0.85
280	**UBE2O**	Ubiquitin-conjugating enzyme E2 O	Medium	Y	-0.17	0.28	0.78
281	**UBR4**	E3 ubiquitin-protein ligase UBR4	Medium	N	0.37	0.66	NaN
282	**USP15**	Ubiquitin carboxyl-terminal hydrolase 15	Medium	N	-0.30	0.16	0.89
283	**USP32**	Ubiquitin carboxyl-terminal hydrolase 32;Ubiquitin carboxyl-terminal hydrolase	Medium	N	0.13	0.63	1.29
284	**USP47**	Ubiquitin carboxyl-terminal hydrolase 47	Medium	N	0.23	0.73	1.26
285	**USP5**	Ubiquitin carboxyl-terminal hydrolase 5	Medium	Y	-0.03	0.30	0.87
286	**USP9X**	Probable ubiquitin carboxyl-terminal hydrolase FAF-X	Medium	N	0.05	0.26	0.93
287	**VCPIP1**	Deubiquitinating protein VCIP135	Medium	Y	-0.10	0.29	0.85
288	**VDAC1**	Voltage-dependent anion-selective channel protein 1	Medium	N	0.17	1.06	NaN
289	**ZNF217**	Zinc finger protein 217	Medium	N	0.00	0.58	1.46

2.4 Supporting Table S4. High- and medium- confidence targets of sulforaphane in the MDA-MB-231 cell line

	Gene names	Protein names	Confidence	Conserved	Log$_2$QS		
					Sulforaphane (μM)		
					5	25	100
1	**ACO1**	Cytoplasmic aconitate hydratase	High	N	0.04	0.68	1.73
2	**AIMP2**	Aminoacyl tRNA synthase complex-interacting multifunctional protein 2	High	Y	0.06	0.91	1.40
3	**AIP**	AH receptor-interacting protein;Peptidyl-prolyl cis-trans isomerase	High	Y	0.18	1.10	1.67
4	**AKAP1**	A-kinase anchor protein 1, mitochondrial	High	Y	-0.03	0.71	1.64
5	**AKAP8L**	A-kinase anchor protein 8-like	High	N	0.17	1.24	1.75
6	**ALDH2**	Aldehyde dehydrogenase, mitochondrial	High	N	1.43	2.72	3.62
7	**ALDH9A1**	4-trimethylaminobutyraldehyde dehydrogenase	High	Y	0.62	1.83	2.84

	Gene names	Protein names	Confidence	Conserved	Log$_2$QS		
					Sulforaphane (μM)		
					5	25	100
8	**ANLN**	Actin-binding protein anillin	High	N	-0.02	0.90	2.22
9	**APOBEC3C**	DNA dC->dU-editing enzyme APOBEC-3C	High	N	1.51	4.30	5.13
10	**ARHGAP29**	Rho GTPase-activating protein 29	High	N	0.05	0.81	2.13
11	**ARL1**	ADP-ribosylation factor-like protein 1	High	N	0.11	0.74	1.44
12	**ATP6V1A**	V-type proton ATPase catalytic subunit A	High	Y	0.23	1.22	2.21
13	**BOD1L1**	Biorientation of chromosomes in cell division protein 1-like 1	High	N	-0.13	0.88	2.00
14	**CAPZB**	F-actin-capping protein subunit beta	High	Y	0.00	0.83	1.50
15	**CARM1**	Histone-arginine methyltransferase CARM1	High	Y	0.14	0.79	2.52
16	**CARS**	Cysteine--tRNA ligase, cytoplasmic	High	Y	0.14	1.01	1.72
17	**CAST**	Calpastatin	High	N	-0.31	0.73	1.74
18	**CDK3;CDK2**	Cyclin-dependent kinase 3	High	N	0.22	1.21	1.95
19	**CDKN2AIP**	CDKN2A-interacting protein	High	N	-0.07	0.88	1.50
20	**CPPED1**	Calcineurin-like phosphoesterase domain-containing protein 1	High	Y	0.88	2.65	3.08
21	**CPSF3**	Cleavage and polyadenylation specificity factor subunit 3	High	N	-0.04	0.96	1.38
22	**CPT1A**	Carnitine O-palmitoyltransferase 1, liver isoform	High	Y	-0.02	0.65	1.30
23	**CTSZ**	Cathepsin Z	High	N	-0.32	0.78	2.18
24	**DEGS1**	Sphingolipid delta(4)-desaturase DES1	High	N	0.43	1.72	2.61
25	**DFFA**	DNA fragmentation factor subunit alpha	High	Y	0.14	1.09	2.25
26	**DFNA5**	Non-syndromic hearing impairment protein 5	High	N	0.23	1.59	3.05
27	**DLAT**	Dihydrolipoyllysine-residue acetyltransferase component of pyruvate dehydrogenase complex, mitochondrial	High	N	0.21	0.93	1.73
28	**DLGAP5**	Disks large-associated protein 5	High	N	0.04	0.99	2.08
29	**DNMBP**	Dynamin-binding protein	High	N	-0.07	0.84	1.87
30	**DYNC1LI1**	Cytoplasmic dynein 1 light intermediate chain 1	High	N	-0.13	0.98	1.26
31	**DYNLL1;DYNLL2**	Dynein light chain 1, cytoplasmic;Dynein light chain 2, cytoplasmic	High	Y	-0.01	0.67	1.66
32	**EEA1**	Early endosome antigen 1	High	Y	0.45	1.51	1.95
33	**EGFR**	Epidermal growth factor receptor	High	N	-0.01	0.68	1.56
34	**EIF6**	Eukaryotic translation initiation factor 6	High	N	-0.10	0.81	1.87
35	**ESYT1**	Extended synaptotagmin-1	High	Y	-0.01	0.67	1.36
36	**EXOSC6**	Exosome complex component MTR3	High	Y	0.11	0.92	2.36
37	**FAM120A**	Constitutive coactivator of PPAR-gamma-like protein 1	High	Y	-0.01	0.91	1.82
38	**FAM203A**	Protein FAM203A	High	Y	0.31	1.55	2.74
39	**FNDC3B**	Fibronectin type III domain-containing protein 3B	High	N	-0.04	0.80	1.60
40	**FXR1**	Fragile X mental retardation syndrome-related protein 1	High	Y	0.05	1.00	2.45
41	**FXR2**	Fragile X mental retardation syndrome-related protein 2	High	Y	0.07	0.94	2.74
42	**GOLGA2**	Golgin subfamily A member 2	High	Y	0.09	0.82	1.96
43	**GSDMD**	Gasdermin-D	High	N	0.26	1.37	2.90
44	**GSPT1;GSPT2**	Eukaryotic peptide chain release factor GTP-binding subunit ERF3A;Eukaryotic peptide chain release factor GTP-binding subunit ERF3B	High	N	0.06	0.75	1.47
45	**HAT1**	Histone acetyltransferase type B catalytic subunit	High	Y	-0.02	0.96	1.99
46	**HCCS**	Cytochrome c-type heme lyase	High	Y	0.15	1.05	2.00
47	**HCFC1**	Host cell factor 1;HCF N-terminal chain 1;HCF N-terminal	High	Y	-0.15	0.87	1.98

	Gene names	Protein names	Confidence	Conserved	Log$_2$QS		
					Sulforaphane (µM)		
					5	25	100
		chain 2;HCF N-terminal chain 3;HCF N-terminal chain 4;HCF N-terminal chain 5;HCF N-terminal chain 6;HCF C-terminal chain 1;HCF C-terminal chain 2;HCF C-terminal chain 3;HCF C-terminal chain 4;HCF C-terminal chain 5;HCF C-terminal chain 6					
48	**HMOX2**	Heme oxygenase 2	High	Y	0.36	1.02	2.04
49	**KEAP1**	Kelch-like ECH-associated protein 1	High	Y	2.41	3.85	5.88
50	**KIAA1524**	Protein CIP2A	High	N	0.13	0.89	1.85
51	**KIF2C**	Kinesin-like protein KIF2C	High	N	0.08	1.09	2.01
52	**KLC1**	Kinesin light chain 1	High	Y	0.14	0.77	1.63
53	**LUZP1**	Leucine zipper protein 1	High	N	0.12	1.10	2.40
54	**MAP7D3**	MAP7 domain-containing protein 3	High	N	0.14	0.63	1.61
55	**MCMBP**	Mini-chromosome maintenance complex-binding protein	High	Y	-0.01	0.87	2.33
56	**MIF**	Macrophage migration inhibitory factor	High	Y	2.57	2.82	2.64
57	**MKI67**	Antigen KI-67	High	N	0.02	0.79	2.47
58	**MMS19**	MMS19 nucleotide excision repair protein homolog	High	Y	0.07	0.79	1.85
59	**MRPL39**	39S ribosomal protein L39, mitochondrial	High	Y	0.17	1.05	2.22
60	**MTCH2**	Mitochondrial carrier homolog 2	High	Y	0.17	1.04	1.63
61	**MTHFD1L**	Monofunctional C1-tetrahydrofolate synthase, mitochondrial	High	Y	0.13	1.00	1.62
62	**NADK2**	NAD kinase 2, mitochondrial	High	Y	0.49	1.16	1.57
63	**NCAPD2**	Condensin complex subunit 1	High	Y	0.18	1.14	2.20
64	**NCAPH**	Condensin complex subunit 2	High	Y	0.12	0.81	2.02
65	**NDUFS3**	NADH dehydrogenase [ubiquinone] iron-sulfur protein 3, mitochondrial	High	N	0.23	0.92	2.03
66	**NFKB2**	Nuclear factor NF-kappa-B p100 subunit;Nuclear factor NF-kappa-B p52 subunit	High	Y	0.47	1.49	2.17
67	**NR2F2;NR2F1**	COUP transcription factor 2;COUP transcription factor 1	High	Y	-0.14	0.84	1.66
68	**NR3C1**	Glucocorticoid receptor	High	N	-0.05	0.94	1.97
69	**NUP54**	Nucleoporin p54	High	Y	0.20	0.99	2.20
70	**OGFR**	Opioid growth factor receptor	High	Y	0.01	1.01	2.19
71	**OGT**	UDP-N-acetylglucosamine--peptide N-acetylglucosaminyltransferase 110 kDa subunit	High	N	0.42	0.99	1.39
72	**OSBPL3**	Oxysterol-binding protein-related protein 3	High	N	-0.33	0.64	1.05
73	**PALLD**	Palladin	High	N	0.44	1.39	1.14
74	**PGLS**	6-phosphogluconolactonase	High	Y	0.11	0.98	1.90
75	**PHF3**	PHD finger protein 3	High	N	-0.12	0.82	2.11
76	**PLCG1**	1-phosphatidylinositol 4,5-bisphosphate phosphodiesterase gamma-1	High	Y	0.00	0.96	1.92
77	**PLIN3**	Perilipin-3	High	Y	-0.04	0.78	2.05
78	**PML**	Protein PML	High	Y	0.07	1.26	2.48
79	**PNPLA6**	Neuropathy target esterase	High	N	0.01	0.68	0.94
80	**POLRMT**	DNA-directed RNA polymerase, mitochondrial	High	N	0.44	1.55	2.60
81	**PPP2R2A**	Serine/threonine-protein phosphatase 2A 55 kDa regulatory subunit B alpha isoform	High	Y	0.29	1.21	2.32
82	**PREP**	Prolyl endopeptidase	High	Y	0.04	0.75	1.93
83	**PSMD9**	26S proteasome non-ATPase regulatory subunit 9	High	Y	0.11	0.97	2.08
84	**PSME2**	Proteasome activator complex subunit 2	High	Y	0.13	0.96	1.80
85	**PTPN1**	Tyrosine-protein phosphatase non-receptor type 1;Tyrosine-protein phosphatase non-receptor type	High	Y	0.14	0.90	1.51

	Gene names	Protein names	Confidence	Conserved	Log$_2$QS		
					Sulforaphane (µM)		
					5	25	100
86	**PTPN11**	Tyrosine-protein phosphatase non-receptor type 11	High	Y	0.15	0.87	1.83
87	**PXN**	Paxillin	High	Y	0.08	0.75	1.83
88	**RELA**	Transcription factor p65	High	Y	0.15	1.29	1.82
89	**RFTN1**	Raftlin	High	N	0.08	0.70	2.01
90	**RNPEP**	Aminopeptidase B	High	Y	0.33	1.15	1.69
91	**ROCK1**	Rho-associated protein kinase 1	High	N	0.37	0.90	1.88
92	**RPS6KA1**	Ribosomal protein S6 kinase alpha-1;Ribosomal protein S6 kinase	High	Y	0.13	0.72	1.78
93	**RPS6KA3**	Ribosomal protein S6 kinase alpha-3	High	Y	0.03	0.80	2.33
94	**RRM1**	Ribonucleoside-diphosphate reductase large subunit	High	Y	0.17	0.65	1.69
95	**RTN1**	Reticulon-1	High	N	0.19	0.96	1.58
96	**RTN3**	Reticulon-3	High	Y	-0.07	1.13	1.86
97	**RUFY1**	RUN and FYVE domain-containing protein 1	High	Y	-0.01	0.93	1.95
98	**SCCPDH**	Saccharopine dehydrogenase-like oxidoreductase	High	Y	0.08	1.38	2.26
99	**SEC16A**	Protein transport protein Sec16A	High	Y	-0.23	0.74	1.94
100	**SEC63**	Translocation protein SEC63 homolog	High	Y	0.23	0.93	2.04
101	**SERPINB1**	Leukocyte elastase inhibitor	High	N	-0.13	0.72	1.71
102	**SOGA2**	Protein SOGA2	High	N	-0.12	0.72	1.97
103	**SPATS2L**	SPATS2-like protein	High	N	0.02	0.90	1.98
104	**SPG20**	Spartin	High	N	-0.18	0.70	1.89
105	**STAT3**	Signal transducer and activator of transcription 3	High	Y	0.05	0.90	1.86
106	**STK3**	Serine/threonine-protein kinase 3;Serine/threonine-protein kinase 3 36kDa subunit;Serine/threonine-protein kinase 3 20kDa subunit	High	Y	0.35	2.01	2.97
107	**STK4**	Serine/threonine-protein kinase 4;Serine/threonine-protein kinase 4 37kDa subunit;Serine/threonine-protein kinase 4 18kDa subunit	High	Y	0.38	2.15	3.71
108	**SUGT1**	Suppressor of G2 allele of SKP1 homolog	High	Y	0.29	1.16	2.50
109	**TACC1**	Transforming acidic coiled-coil-containing protein 1	High	N	-0.11	0.66	1.75
110	**TBC1D13**	TBC1 domain family member 13	High	N	0.10	0.95	1.77
111	**TBC1D4**	TBC1 domain family member 4	High	N	0.00	0.61	1.84
112	**THNSL1**	Threonine synthase-like 1	High	Y	0.19	1.01	1.75
113	**TMPO**	Lamina-associated polypeptide 2, isoform alpha;Thymopoietin;Thymopentin	High	Y	0.00	0.96	1.87
114	**TNKS1BP1**	182 kDa tankyrase-1-binding protein	High	Y	-0.11	0.75	1.81
115	**TNS3**	Tensin-3	High	N	0.18	1.50	2.35
116	**TPX2**	Targeting protein for Xklp2	High	N	-0.08	0.70	2.17
117	**USP10**	Ubiquitin carboxyl-terminal hydrolase 10	High	Y	0.33	1.21	2.14
118	**VDAC2**	Voltage-dependent anion-selective channel protein 2	High	Y	-0.14	0.85	1.76
119	**VDAC3**	Voltage-dependent anion-selective channel protein 3	High	Y	0.00	1.17	2.34
120	**YAP1**	Yorkie homolog	High	N	0.19	0.84	1.64
121	**ZNF185**	Zinc finger protein 185	High	N	-0.18	0.75	2.27
122	**AARS**	Alanine--tRNA ligase, cytoplasmic	Medium	N	0.08	0.46	1.14
123	**ABCB7**	ATP-binding cassette sub-family B member 7, mitochondrial	Medium	N	-0.17	0.43	0.86
124	**ABCF1**	ATP-binding cassette sub-family F member 1	Medium	N	NaN	1.14	NaN
125	**ACAD9**	Acyl-CoA dehydrogenase family member 9, mitochondrial	Medium	Y	0.19	0.56	1.45

	Gene names	Protein names	Confidence	Conserved	Log$_2$QS		
					Sulforaphane (μM)		
					5	25	100
126	**ACIN1**	Apoptotic chromatin condensation inducer in the nucleus	Medium	N	0.21	1.36	1.37
127	**ACLY**	ATP-citrate synthase	Medium	N	-0.20	0.40	1.26
128	**ACOT9**	Acyl-coenzyme A thioesterase 9, mitochondrial	Medium	N	0.00	0.28	0.71
129	**ACSL3**	Long-chain-fatty-acid--CoA ligase 3	Medium	N	-0.03	0.63	1.43
130	**ADAR**	Double-stranded RNA-specific adenosine deaminase	Medium	Y	0.11	0.43	1.10
131	**ADRM1**	Proteasomal ubiquitin receptor ADRM1	Medium	Y	-0.17	0.73	1.27
132	**ADSS**	Adenylosuccinate synthetase isozyme 2	Medium	N	0.15	0.59	1.32
133	**AFAP1**	Actin filament-associated protein 1	Medium	N	0.21	0.73	1.60
134	**AGPAT9**	Glycerol-3-phosphate acyltransferase 3	Medium	N	0.05	1.11	NaN
135	**AHCY**	Adenosylhomocysteinase	Medium	Y	0.44	0.90	1.58
136	**AJUBA**	LIM domain-containing protein ajuba	Medium	N	0.02	0.38	1.11
137	**AKAP11**	A-kinase anchor protein 11	Medium	N	0.05	1.15	NaN
138	**AKAP13**	A-kinase anchor protein 13	Medium	N	0.01	0.87	NaN
139	**AKAP2**	A-kinase anchor protein 2	Medium	N	-0.16	0.85	1.07
140	**AKAP8**	A-kinase anchor protein 8	Medium	N	-0.22	0.61	1.82
141	**AMPD2**	AMP deaminase 2	Medium	N	-0.32	0.69	1.37
142	**ANAPC7**	Anaphase-promoting complex subunit 7	Medium	N	0.18	1.11	2.38
143	**AP2A1**	AP-2 complex subunit alpha-1	Medium	N	0.03	1.11	0.70
144	**ARAP1**	Arf-GAP with Rho-GAP domain, ANK repeat and PH domain-containing protein 1	Medium	N	-0.55	0.30	1.19
145	**ARHGEF 28**	Rho guanine nucleotide exchange factor 28	Medium	N	0.16	0.66	0.78
146	**ARMCX2**	Armadillo repeat-containing X-linked protein 2	Medium	N	0.32	1.88	2.54
147	**ASNS**	Asparagine synthetase [glutamine-hydrolyzing]	Medium	Y	0.24	0.44	1.40
148	**ATAD3B; ATAD3A**	ATPase family AAA domain-containing protein 3B;ATPase family AAA domain-containing protein 3A	Medium	N	0.12	1.26	NaN
149	**ATP2A2**	Sarcoplasmic/endoplasmic reticulum calcium ATPase 2	Medium	N	0.03	0.43	0.83
150	**ATXN2**	Ataxin-2	Medium	N	0.04	0.71	1.71
151	**ATXN2L**	Ataxin-2-like protein	Medium	Y	0.02	0.63	1.59
152	**AXL**	Tyrosine-protein kinase receptor UFO	Medium	N	0.36	0.52	1.57
153	**BABAM1**	BRISC and BRCA1-A complex member 1	Medium	N	0.01	0.52	1.28
154	**BAG3**	BAG family molecular chaperone regulator 3	Medium	Y	-0.08	0.59	1.72
155	**BAG6**	Large proline-rich protein BAG6	Medium	Y	-0.44	0.03	0.62
156	**BCAR3**	Breast cancer anti-estrogen resistance protein 3	Medium	N	-0.20	0.65	1.85
157	**BLMH**	Bleomycin hydrolase	Medium	N	0.02	0.54	1.28
158	**BOLA2**	BolA-like protein 2	Medium	N	0.31	1.53	NaN
159	**BTD**	Biotinidase	Medium	Y	0.08	2.25	NaN
160	**BUB1B**	Mitotic checkpoint serine/threonine-protein kinase BUB1 beta	Medium	N	-0.15	0.72	NaN
161	**CACYBP**	Calcyclin-binding protein	Medium	N	0.27	0.14	0.74
162	**CAD**	CAD protein;Glutamine-dependent carbamoyl-phosphate synthase;Aspartate carbamoyltransferase;Dihydroorotase	Medium	Y	0.22	0.71	1.47
163	**CAPRIN1**	Caprin-1	Medium	N	-0.11	0.29	0.74
164	**CBL**	E3 ubiquitin-protein ligase CBL	Medium	N	-0.14	0.55	1.26
165	**CDC27**	Cell division cycle protein 27 homolog	Medium	N	-0.01	0.59	1.06
166	**CEP170**	Centrosomal protein of 170 kDa	Medium	N	-0.14	0.32	1.63

	Gene names	Protein names	Confidence	Conserved	Log$_2$QS		
					Sulforaphane (µM)		
					5	25	100
167	**CHORDC1**	Cysteine and histidine-rich domain-containing protein 1	Medium	N	-0.48	0.37	1.35
168	**CIAPIN1**	Anamorsin	Medium	N	-0.03	0.28	1.17
169	**CKAP4**	Cytoskeleton-associated protein 4	Medium	Y	-0.03	0.54	1.15
170	**CKAP5**	Cytoskeleton-associated protein 5	Medium	N	-0.17	0.64	1.19
171	**CNN2**	Calponin-2	Medium	N	-0.18	0.06	1.05
172	**COPB1**	Coatomer subunit beta	Medium	N	0.07	0.43	0.85
173	**CORO1C**	Coronin-1C;Coronin	Medium	N	-0.10	0.47	0.92
174	**CPNE1**	Copine-1	Medium	N	-0.01	0.28	0.82
175	**CPOX**	Coproporphyrinogen-III oxidase, mitochondrial	Medium	N	-0.04	0.60	1.00
176	**CREM;ATF1;CREB1**	Cyclic AMP-dependent transcription factor ATF-1;Cyclic AMP-responsive element-binding protein 1	Medium	N	0.01	0.95	1.92
177	**CRKL**	Crk-like protein	Medium	Y	0.37	1.14	1.83
178	**CSTB**	Cystatin-B	Medium	N	0.18	1.45	2.80
179	**CTH**	Cystathionine gamma-lyase	Medium	N	0.70	0.23	1.01
180	**CTPS1**	CTP synthase 1	Medium	N	0.15	0.57	0.89
181	**CTSB**	Cathepsin B;Cathepsin B light chain;Cathepsin B heavy chain	Medium	Y	-0.27	0.36	0.84
182	**CTSC**	Dipeptidyl peptidase 1;Dipeptidyl peptidase 1 exclusion domain chain;Dipeptidyl peptidase 1 heavy chain;Dipeptidyl peptidase 1 light chain	Medium	Y	0.09	0.70	2.71
183	**CTTN**	Src substrate cortactin	Medium	Y	-0.29	0.36	1.20
184	**CYFIP1;CYFIP2**	Cytoplasmic FMR1-interacting protein 1;Cytoplasmic FMR1-interacting protein 2	Medium	N	-0.06	0.50	0.97
185	**DBN1**	Drebrin	Medium	N	-0.11	0.41	1.26
186	**DBNL**	Drebrin-like protein	Medium	N	-0.19	0.61	1.31
187	**DDX24**	ATP-dependent RNA helicase DDX24	Medium	N	0.07	0.55	1.61
188	**DDX47**	Probable ATP-dependent RNA helicase DDX47	Medium	N	-0.15	0.36	1.43
189	**DECR1**	2,4-dienoyl-CoA reductase, mitochondrial	Medium	N	0.03	0.34	0.59
190	**DEK**	Protein DEK	Medium	N	-0.22	0.35	1.13
191	**DENR**	Density-regulated protein	Medium	N	-0.19	0.19	1.03
192	**DHX15**	Putative pre-mRNA-splicing factor ATP-dependent RNA helicase DHX15	Medium	Y	0.09	0.67	0.96
193	**DHX30**	Putative ATP-dependent RNA helicase DHX30	Medium	N	0.01	0.44	1.26
194	**DIAPH1**	Protein diaphanous homolog 1	Medium	Y	-0.28	0.32	1.08
195	**DIAPH3**	Protein diaphanous homolog 3	Medium	N	-0.07	0.84	1.26
196	**DIDO1**	Death-inducer obliterator 1	Medium	N	-0.18	0.66	1.85
197	**DLGAP4**	Disks large-associated protein 4	Medium	N	-0.05	1.13	NaN
198	**DNM1L**	Dynamin-1-like protein	Medium	Y	-0.13	0.27	1.12
199	**DNPEP**	Aspartyl aminopeptidase	Medium	Y	-0.13	0.66	1.79
200	**DPYSL2**	Dihydropyrimidinase-related protein 2	Medium	N	-0.09	0.56	1.09
201	**DSTN**	Destrin	Medium	Y	-0.10	0.41	1.16
202	**DUT**	Deoxyuridine 5-triphosphate nucleotidohydrolase, mitochondrial	Medium	Y	0.29	0.76	1.42
203	**EDC4**	Enhancer of mRNA-decapping protein 4	Medium	Y	0.00	0.68	1.44
204	**EFHD2**	EF-hand domain-containing protein D2	Medium	N	-0.12	0.43	1.65
205	**EIF3G**	Eukaryotic translation initiation factor 3 subunit G	Medium	Y	-0.20	0.31	1.06

	Gene names	Protein names	Confidence	Conserved	Log$_2$QS		
					Sulforaphane (µM)		
					5	25	100
206	**EIF5B**	Eukaryotic translation initiation factor 5B	Medium	N	0.10	0.01	0.94
207	**ELAC2**	Zinc phosphodiesterase ELAC protein 2	Medium	Y	0.07	0.78	1.59
208	**EPRS**	Bifunctional glutamate/proline--tRNA ligase;Glutamate--tRNA ligase;Proline--tRNA ligase	Medium	Y	0.02	0.42	1.07
209	**EPS8L2**	Epidermal growth factor receptor kinase substrate 8-like protein 2	Medium	Y	0.09	0.68	1.59
210	**ERCC6L**	DNA excision repair protein ERCC-6-like	Medium	N	-0.07	0.54	1.81
211	**EXOSC10**	Exosome component 10	Medium	N	0.15	0.63	1.61
212	**FAM129A**	Protein Niban	Medium	N	-0.01	0.59	1.66
213	**FASN**	Fatty acid synthase;[Acyl-carrier-protein] S-acetyltransferase;[Acyl-carrier-protein] S-malonyltransferase;3-oxoacyl-[acyl-carrier-protein] synthase;3-oxoacyl-[acyl-carrier-protein] reductase;3-hydroxyacyl-[acyl-carrier-protein] dehydratase;Enoyl-[acyl-carrier-protein] reductase;Oleoyl-[acyl-carrier-protein] hydrolase	Medium	Y	0.14	0.63	0.98
214	**FKBP8**	Peptidyl-prolyl cis-trans isomerase FKBP8;Peptidyl-prolyl cis-trans isomerase	Medium	N	-0.02	0.73	1.50
215	**FLII**	Protein flightless-1 homolog	Medium	Y	-0.01	0.41	1.35
216	**FLNB**	Filamin-B	Medium	N	-0.05	0.26	0.68
217	**FMR1**	Fragile X mental retardation protein 1	Medium	Y	0.09	1.07	NaN
218	**FNBP1**	Formin-binding protein 1	Medium	N	0.26	0.81	NaN
219	**FRMD6**	FERM domain-containing protein 6	Medium	N	-0.04	0.28	1.11
220	**FTO**	Alpha-ketoglutarate-dependent dioxygenase FTO	Medium	N	-0.10	0.41	1.64
221	**GARS**	Glycine--tRNA ligase	Medium	Y	0.13	0.48	1.12
222	**GART**	Trifunctional purine biosynthetic protein adenosine-3;Phosphoribosylamine--glycine ligase;Phosphoribosylformylglycinamidine cyclo-ligase;Phosphoribosylglycinamide formyltransferase	Medium	Y	0.05	0.28	0.80
223	**GBE1**	1,4-alpha-glucan-branching enzyme	Medium	N	0.01	0.54	1.40
224	**GEMIN5**	Gem-associated protein 5	Medium	Y	0.06	0.54	1.32
225	**GFPT1**	Glutamine--fructose-6-phosphate aminotransferase [isomerizing] 1	Medium	Y	0.10	0.67	1.61
226	**GLOD4**	Glyoxalase domain-containing protein 4	Medium	Y	-0.02	0.70	1.78
227	**GMPS**	GMP synthase [glutamine-hydrolyzing]	Medium	Y	0.12	0.37	0.97
228	**GNE**	Bifunctional UDP-N-acetylglucosamine 2-epimerase/N-acetylmannosamine kinase;UDP-N-acetylglucosamine 2-epimerase (hydrolyzing);N-acetylmannosamine kinase	Medium	N	-0.15	0.37	1.09
229	**GPD2**	Glycerol-3-phosphate dehydrogenase, mitochondrial	Medium	N	-0.13	0.24	0.79
230	**GSTM3**	Glutathione S-transferase Mu 3	Medium	Y	0.00	0.55	1.02
231	**GTF2I**	General transcription factor II-I	Medium	N	-0.32	0.35	0.96
232	**HDAC1**	Histone deacetylase 1	Medium	N	-0.13	0.43	0.87
233	**HDGF**	Hepatoma-derived growth factor	Medium	Y	-0.01	0.48	1.28
234	**HEATR3**	HEAT repeat-containing protein 3	Medium	N	0.21	1.33	2.85
235	**HECTD1**	E3 ubiquitin-protein ligase HECTD1	Medium	Y	0.14	0.50	0.96
236	**HEXIM1**	Protein HEXIM1	Medium	N	-0.30	0.51	1.48
237	**HLA-A**	HLA class I histocompatibility antigen, A-2 alpha chain;HLA class I histocompatibility antigen, A-69 alpha chain;HLA class I histocompatibility antigen, A-74 alpha chain;HLA class I histocompatibility antigen, A-68 alpha chain;HLA class I histocompatibility antigen, A-31 alpha chain;HLA class I histocompatibility antigen, A-32 alpha chain;HLA class I histocompatibility antigen, A-33 alpha chain;HLA class I histocompatibility antigen, A-29 alpha chain	Medium	N	-0.31	0.48	1.12

	Gene names	Protein names	Confidence	Conserved	Log$_2$QS		
					Sulforaphane (µM)		
					5	25	100
238	**HLA-C**	HLA class I histocompatibility antigen, Cw-12 alpha chain;HLA class I histocompatibility antigen, Cw-15 alpha chain;HLA class I histocompatibility antigen, Cw-16 alpha chain;HLA class I histocompatibility antigen, Cw-14 alpha chain;HLA class I histocompatibility antigen, Cw-8 alpha chain;HLA class I histocompatibility antigen, Cw-6 alpha chain;HLA class I histocompatibility antigen, Cw-4 alpha chain;HLA class I histocompatibility antigen, Cw-5 alpha chain;HLA class I histocompatibility antigen, Cw-18 alpha chain	Medium	N	-0.22	0.75	1.37
239	**HNRNPF**	Heterogeneous nuclear ribonucleoprotein F;Heterogeneous nuclear ribonucleoprotein F, N-terminally processed	Medium	Y	0.00	0.56	1.13
240	**HPCAL1; HPCA**	Hippocalcin-like protein 1;Neuron-specific calcium-binding protein hippocalcin	Medium	N	0.01	0.47	1.32
241	**HPRT1**	Hypoxanthine-guanine phosphoribosyltransferase	Medium	Y	0.21	0.47	1.08
242	**HSD17B10**	3-hydroxyacyl-CoA dehydrogenase type-2	Medium	Y	0.08	0.71	1.49
243	**HSPA4L**	Heat shock 70 kDa protein 4L	Medium	N	0.10	0.34	0.85
244	**HSPB1**	Heat shock protein beta-1	Medium	Y	0.06	0.64	2.03
245	**HSPBP1**	Hsp70-binding protein 1	Medium	Y	0.16	0.57	1.62
246	**HSPH1**	Heat shock protein 105 kDa	Medium	Y	-0.01	0.26	0.81
247	**HUWE1**	E3 ubiquitin-protein ligase HUWE1	Medium	Y	0.10	0.54	1.20
248	**IFI16**	Gamma-interferon-inducible protein 16	Medium	N	-0.29	0.65	1.26
249	**IFIT3**	Interferon-induced protein with tetratricopeptide repeats 3	Medium	N	-0.01	0.59	1.31
250	**IMPDH2**	Inosine-5-monophosphate dehydrogenase 2	Medium	Y	0.09	0.40	1.01
251	**INF2**	Inverted formin-2	Medium	Y	-0.26	0.46	1.39
252	**IPO4**	Importin-4	Medium	N	0.31	0.34	0.82
253	**IPO5**	Importin-5	Medium	Y	0.16	0.63	1.21
254	**ISOC2**	Isochorismatase domain-containing protein 2, mitochondrial	Medium	Y	0.05	0.55	1.68
255	**KDM3B**	Lysine-specific demethylase 3B	Medium	Y	0.09	1.09	NaN
256	**KHSRP**	Far upstream element-binding protein 2	Medium	Y	-0.11	0.57	1.18
257	**KIF20A**	Kinesin-like protein KIF20A	Medium	N	-0.24	0.56	0.33
258	**KIF4A**	Chromosome-associated kinesin KIF4A	Medium	N	-0.25	0.66	1.42
259	**KIFC1**	Kinesin-like protein KIFC1	Medium	N	-0.28	-0.15	1.09
260	**LACTB**	Serine beta-lactamase-like protein LACTB, mitochondrial	Medium	Y	0.09	1.00	NaN
261	**LANCL2**	LanC-like protein 2	Medium	Y	0.18	1.09	NaN
262	**LARP1**	La-related protein 1	Medium	Y	0.02	0.71	1.78
263	**LARP4**	La-related protein 4	Medium	Y	0.08	0.45	1.71
264	**LARP4B**	La-related protein 4B	Medium	Y	-0.10	0.50	1.44
265	**LCMT1**	Leucine carboxyl methyltransferase 1	Medium	Y	0.16	1.26	3.20
266	**LGALS1**	Galectin-1	Medium	Y	-0.06	0.39	1.02
267	**LIG1**	DNA ligase 1;DNA ligase	Medium	N	-0.22	0.26	0.96
268	**LPCAT2**	Lysophosphatidylcholine acyltransferase 2	Medium	N	-0.02	0.60	1.07
269	**LRRFIP1**	Leucine-rich repeat flightless-interacting protein 1	Medium	N	-0.14	0.41	1.57
270	**MAGED2**	Melanoma-associated antigen D2	Medium	N	0.05	0.70	1.48
271	**MALT1**	Mucosa-associated lymphoid tissue lymphoma translocation protein 1	Medium	N	0.10	0.98	2.08
272	**MAP1B**	Microtubule-associated protein 1B;MAP1B heavy chain;MAP1 light chain LC1	Medium	N	-0.11	0.33	1.13
273	**MAP1S**	Microtubule-associated protein 1S;MAP1S heavy chain;MAP1S light chain	Medium	N	-0.13	0.80	1.09

	Gene names	Protein names	Confidence	Conserved	Log_2QS		
					Sulforaphane (µM)		
					5	25	100
274	**MAP4**	Microtubule-associated protein;Microtubule-associated protein 4	Medium	N	-0.18	0.47	1.50
275	**MARCKS**	Myristoylated alanine-rich C-kinase substrate	Medium	N	-0.01	0.29	1.05
276	**MASTL**	Serine/threonine-protein kinase greatwall	Medium	N	-0.01	0.70	NaN
277	**MAVS**	Mitochondrial antiviral-signaling protein	Medium	Y	-0.35	0.59	1.46
278	**MCM3**	DNA replication licensing factor MCM3	Medium	Y	-0.37	0.18	0.88
279	**MICAL2**	Protein-methionine sulfoxide oxidase MICAL2	Medium	N	-0.22	0.54	1.28
280	**MPRIP**	Myosin phosphatase Rho-interacting protein	Medium	N	-0.17	0.68	1.34
281	**MSH2**	DNA mismatch repair protein Msh2	Medium	N	-0.05	0.74	1.00
282	**MTA2**	Metastasis-associated protein MTA2	Medium	Y	-0.16	0.63	1.52
283	**MYH9**	Myosin-9	Medium	Y	-0.09	0.29	0.82
284	**MYL6**	Myosin light polypeptide 6	Medium	Y	-0.04	0.37	1.31
285	**NASP**	Nuclear autoantigenic sperm protein	Medium	N	-0.05	0.21	0.81
286	**NBN**	Nibrin	Medium	N	-0.20	0.71	1.87
287	**NCAPG**	Condensin complex subunit 3	Medium	N	-0.20	0.64	1.13
288	**NCEH1**	Neutral cholesterol ester hydrolase 1	Medium	N	0.09	0.46	1.06
289	**NEDD4L**	E3 ubiquitin-protein ligase NEDD4-like	Medium	N	0.24	0.68	1.53
290	**NHLRC2**	NHL repeat-containing protein 2	Medium	N	0.13	0.55	0.99
291	**NKRF**	NF-kappa-B-repressing factor	Medium	N	-0.21	0.11	1.02
292	**NONO**	Non-POU domain-containing octamer-binding protein	Medium	Y	-0.29	0.40	1.29
293	**NOP58**	Nucleolar protein 58	Medium	N	0.01	0.49	1.38
294	**NPEPPS**	Puromycin-sensitive aminopeptidase	Medium	N	-0.05	0.27	0.94
295	**NRD1**	Nardilysin	Medium	N	-0.04	0.51	1.15
296	**NSUN2**	tRNA (cytosine(34)-C(5))-methyltransferase	Medium	N	0.05	0.42	0.94
297	**NUDC**	Nuclear migration protein nudC	Medium	Y	0.03	0.40	1.26
298	**NUDCD1**	NudC domain-containing protein 1	Medium	Y	0.18	0.68	1.64
299	**NUP153**	Nuclear pore complex protein Nup153	Medium	N	-0.05	0.28	1.32
300	**NUP155**	Nuclear pore complex protein Nup155	Medium	Y	0.22	0.81	1.67
301	**NUP93**	Nuclear pore complex protein Nup93	Medium	N	0.45	0.71	0.44
302	**OSBP**	Oxysterol-binding protein 1	Medium	N	0.02	0.33	1.09
303	**OTUD6B**	OTU domain-containing protein 6B	Medium	Y	0.08	0.66	1.35
304	**PABPC4**	Polyadenylate-binding protein 4	Medium	N	0.00	0.66	0.93
305	**PBK**	Lymphokine-activated killer T-cell-originated protein kinase	Medium	N	-0.07	0.69	2.10
306	**PCBP1**	Poly(rC)-binding protein 1	Medium	Y	-0.05	0.69	1.81
307	**PCBP2**	Poly(rC)-binding protein 2	Medium	Y	-0.25	0.38	0.98
308	**PCMT1**	Protein-L-isoaspartate(D-aspartate) O-methyltransferase;Protein-L-isoaspartate O-methyltransferase	Medium	Y	0.07	0.67	1.90
309	**PCYT1A; PCYT1B**	Choline-phosphate cytidylyltransferase A;Choline-phosphate cytidylyltransferase B	Medium	Y	-0.09	0.94	2.15
310	**PDCD6IP**	Programmed cell death 6-interacting protein	Medium	Y	-0.04	0.35	1.01
311	**PDE12**	2,5-phosphodiesterase 12	Medium	Y	-0.03	0.54	1.07
312	**PDS5A**	Sister chromatid cohesion protein PDS5 homolog A	Medium	N	-0.26	0.55	0.91
313	**PEPD**	Xaa-Pro dipeptidase	Medium	N	-0.16	0.67	0.97
314	**PFAS**	Phosphoribosylformylglycinamidine synthase	Medium	Y	0.11	0.78	1.65

	Gene names	Protein names	Confidence	Conserved	Log$_2$QS		
					Sulforaphane (μM)		
					5	25	100
315	**PGPEP1**	Pyroglutamyl-peptidase 1	Medium	Y	-0.11	0.40	1.62
316	**PLEC**	Plectin	Medium	N	-0.02	0.35	0.99
317	**PLS3**	Plastin-3	Medium	N	0.05	0.57	1.12
318	**POLR2B**	DNA-directed RNA polymerase;DNA-directed RNA polymerase II subunit RPB2	Medium	N	0.05	0.38	1.37
319	**PPM1G**	Protein phosphatase 1G	Medium	Y	0.11	0.65	1.70
320	**PPME1**	Protein phosphatase methylesterase 1	Medium	Y	-0.02	0.18	0.73
321	**PPP1R12A**	Protein phosphatase 1 regulatory subunit 12A	Medium	N	-0.19	0.57	0.86
322	**PPP1R18**	Phostensin	Medium	N	-0.30	0.64	1.17
323	**PPP2R5D**	Serine/threonine-protein phosphatase 2A 56 kDa regulatory subunit delta isoform	Medium	N	0.12	0.63	1.28
324	**PPP4R1**	Serine/threonine-protein phosphatase 4 regulatory subunit 1	Medium	N	0.04	0.79	1.77
325	**PPP6R3**	Serine/threonine-protein phosphatase 6 regulatory subunit 3	Medium	Y	-0.09	0.36	1.27
326	**PRKDC**	DNA-dependent protein kinase catalytic subunit	Medium	Y	-0.16	0.72	1.17
327	**PRMT1**	Protein arginine N-methyltransferase 1	Medium	Y	-0.06	0.43	1.34
328	**PRMT3**	Protein arginine N-methyltransferase 3	Medium	Y	NaN	1.00	NaN
329	**PRPF40A**	Pre-mRNA-processing factor 40 homolog A	Medium	N	-0.29	0.29	0.76
330	**PRRC2C**	Protein PRRC2C	Medium	Y	-0.07	0.69	1.54
331	**PTGES3**	Prostaglandin E synthase 3	Medium	Y	0.11	0.72	1.97
332	**PTPN12**	Tyrosine-protein phosphatase non-receptor type 12	Medium	N	-0.31	0.64	1.61
333	**PUM1**	Pumilio homolog 1	Medium	N	-0.01	0.75	1.08
334	**PYCR2**	Pyrroline-5-carboxylate reductase 2	Medium	N	0.00	0.93	NaN
335	**PYGB**	Glycogen phosphorylase, brain form	Medium	Y	-0.16	0.50	0.86
336	**QARS**	Glutamine--tRNA ligase	Medium	Y	-0.06	0.48	1.06
337	**RANBP3**	Ran-binding protein 3	Medium	N	-0.19	0.38	1.38
338	**RANBP6**	Ran-binding protein 6	Medium	Y	NaN	1.09	NaN
339	**RARS**	Arginine--tRNA ligase, cytoplasmic	Medium	Y	0.01	0.51	1.04
340	**RASA3**	Ras GTPase-activating protein 3	Medium	N	0.04	0.96	NaN
341	**RB1**	Retinoblastoma-associated protein	Medium	N	-0.17	0.39	0.92
342	**RBM25**	RNA-binding protein 25	Medium	N	-0.15	0.43	1.11
343	**RBPJ**	Recombining binding protein suppressor of hairless	Medium	N	-0.05	0.79	1.37
344	**RCC2**	Protein RCC2	Medium	Y	-0.01	0.71	1.15
345	**REEP5**	Receptor expression-enhancing protein 5	Medium	N	0.00	0.30	0.74
346	**RIN1**	Ras and Rab interactor 1	Medium	N	0.12	1.14	0.74
347	**RNMT**	mRNA cap guanine-N7 methyltransferase	Medium	N	0.06	0.63	1.58
348	**RPS6KA4**	Ribosomal protein S6 kinase;Ribosomal protein S6 kinase alpha-4	Medium	N	0.53	1.40	NaN
349	**RRBP1**	Ribosome-binding protein 1	Medium	Y	-0.01	0.63	1.14
350	**S100A4**	Protein S100-A4	Medium	N	-0.66	0.75	0.17
351	**SAFB**	Scaffold attachment factor B1	Medium	N	-0.20	0.56	1.26
352	**SAFB2**	Scaffold attachment factor B2	Medium	N	-0.27	0.45	1.26
353	**SCAF11**	Protein SCAF11	Medium	N	-0.15	0.70	1.65
354	**SEC24C**	Protein transport protein Sec24C	Medium	Y	-0.05	0.69	1.20
355	**SEC61B**	Protein transport protein Sec61 subunit beta	Medium	N	0.04	0.11	1.77

	Gene names	Protein names	Confidence	Conserved	Log$_2$QS		
					Sulforaphane (μM)		
					5	25	100
356	**SEPT9**	Septin-9	Medium	N	-0.31	0.37	1.14
357	**SERPINB6**	Serpin B6	Medium	N	0.00	0.52	1.37
358	**SERPINB9**	Serpin B9	Medium	N	-0.29	0.66	0.94
359	**SF1**	Splicing factor 1	Medium	N	0.09	0.55	1.63
360	**SFN**	14-3-3 protein sigma	Medium	Y	-0.13	0.67	1.32
361	**SLC9A3R2**	Na(+)/H(+) exchange regulatory cofactor NHE-RF2	Medium	N	-0.29	0.23	0.72
362	**SLK**	STE20-like serine/threonine-protein kinase	Medium	N	0.10	0.53	1.45
363	**SMC2**	Structural maintenance of chromosomes protein 2	Medium	Y	-0.38	0.26	0.98
364	**SMCHD1**	Structural maintenance of chromosomes flexible hinge domain-containing protein 1	Medium	N	-0.06	0.79	1.77
365	**SMURF2**	E3 ubiquitin-protein ligase SMURF2	Medium	N	-0.02	0.52	1.28
366	**SOAT1**	Sterol O-acyltransferase 1	Medium	N	-0.12	0.83	NaN
367	**SP100**	Nuclear autoantigen Sp-100	Medium	N	-0.14	0.52	1.65
368	**SPTLC2**	Serine palmitoyltransferase 2	Medium	Y	0.05	0.82	1.97
369	**SQSTM1**	Sequestosome-1	Medium	Y	-0.05	0.41	1.43
370	**SRPK1**	SRSF protein kinase 1	Medium	N	0.21	0.79	0.91
371	**SRPR**	Signal recognition particle receptor subunit alpha	Medium	N	0.07	0.74	1.63
372	**STAT1**	Signal transducer and activator of transcription 1-alpha/beta	Medium	Y	0.13	0.75	0.98
373	**STAU1**	Double-stranded RNA-binding protein Staufen homolog 1	Medium	N	-0.10	0.28	0.87
374	**STK10**	Serine/threonine-protein kinase 10	Medium	N	-0.02	0.38	1.39
375	**TACC3**	Transforming acidic coiled-coil-containing protein 3	Medium	Y	-0.17	0.43	1.07
376	**TAGLN2**	Transgelin-2	Medium	Y	0.44	0.91	1.79
377	**TBC1D15**	TBC1 domain family member 15	Medium	Y	-0.09	0.39	0.97
378	**TBCD**	Tubulin-specific chaperone D	Medium	Y	0.06	0.23	1.06
379	**TCOF1**	Treacle protein	Medium	N	-0.12	0.50	1.02
380	**THYN1**	Thymocyte nuclear protein 1	Medium	N	0.21	1.28	NaN
381	**TIGAR**	Fructose-2,6-bisphosphatase TIGAR	Medium	Y	0.04	1.32	NaN
382	**TK1**	Thymidine kinase, cytosolic;Thymidine kinase	Medium	N	-0.42	0.16	1.16
383	**TP53BP1**	Tumor suppressor p53-binding protein 1	Medium	N	0.07	0.48	1.53
384	**TPM3;DKFZp686J1372**	Tropomyosin alpha-3 chain	Medium	Y	-0.09	0.78	1.06
385	**TRIM25**	E3 ubiquitin/ISG15 ligase TRIM25	Medium	Y	0.02	0.79	2.75
386	**TSC22D2**	TSC22 domain family protein 2	Medium	N	-0.19	0.41	1.31
387	**TTC1**	Tetratricopeptide repeat protein 1	Medium	N	-0.05	0.55	1.15
388	**TXLNA**	Alpha-taxilin	Medium	N	-0.19	0.78	1.27
389	**TXLNG**	Gamma-taxilin	Medium	Y	-0.04	0.21	1.02
390	**TXNRD1**	Thioredoxin reductase 1, cytoplasmic	Medium	Y	-0.18	0.41	0.97
391	**U2AF1**	Splicing factor U2AF 35 kDa subunit	Medium	N	-0.04	0.29	0.87
392	**UBA6**	Ubiquitin-like modifier-activating enzyme 6	Medium	Y	-0.13	0.42	1.65
393	**UBAP2**	Ubiquitin-associated protein 2	Medium	N	0.00	0.57	1.38
394	**UBE2O**	Ubiquitin-conjugating enzyme E2 O	Medium	Y	0.53	1.28	1.68
395	**USP24**	Ubiquitin carboxyl-terminal hydrolase 24	Medium	N	0.04	1.14	NaN

	Gene names	Protein names	Confidence	Conserved	Log$_2$QS		
					Sulforaphane (μM)		
					5	25	100
396	**USP28**	Ubiquitin carboxyl-terminal hydrolase 28;Ubiquitin carboxyl-terminal hydrolase	Medium	N	-0.20	0.35	0.93
397	**USP5**	Ubiquitin carboxyl-terminal hydrolase 5	Medium	Y	-0.07	0.56	1.38
398	**USP7**	Ubiquitin carboxyl-terminal hydrolase 7;Ubiquitin carboxyl-terminal hydrolase	Medium	Y	0.00	0.32	1.01
399	**USP9X;USP9Y**	Probable ubiquitin carboxyl-terminal hydrolase FAF-X;Probable ubiquitin carboxyl-terminal hydrolase FAF-Y	Medium	N	0.04	0.56	1.07
400	**VASP**	Vasodilator-stimulated phosphoprotein	Medium	N	-0.11	0.24	0.98
401	**VCPIP1**	Deubiquitinating protein VCIP135	Medium	Y	0.21	0.87	NaN
402	**VEZT**	Vezatin	Medium	N	-0.15	0.75	1.40
403	**WAPAL**	Wings apart-like protein homolog	Medium	N	-0.12	0.24	1.04
404	**WDHD1**	WD repeat and HMG-box DNA-binding protein 1	Medium	N	0.14	0.74	NaN
405	**WDR62**	WD repeat-containing protein 62	Medium	N	0.29	0.88	NaN
406	**WDR77**	Methylosome protein 50	Medium	Y	-0.05	0.52	1.38
407	**XPO5**	Exportin-5	Medium	Y	0.43	1.43	NaN
408	**YRDC**	YrdC domain-containing protein, mitochondrial	Medium	N	-0.05	0.92	1.68
409	**ZC3H11A**	Zinc finger CCCH domain-containing protein 11A	Medium	N	-0.21	0.63	1.45
410	**ZNF638**	Zinc finger protein 638	Medium	N	0.42	1.18	1.76
411	**ZW10**	Centromere/kinetochore protein zw10 homolog	Medium	N	1.40	-0.06	0.62

3 Chemical synthesis methods

3.1 General Information

The reagents used during all synthesis processes were obtained from commercial sources (Sigma-Aldrich, VWR and Fisher Scientific) and used without further purification. Reactions were followed by TLC using aluminium-backed silica plate (Merck, TLC Silica Gel 60, F254) and visualised under UV irradiation at 254 nm or using a variety of stains. Flash column chromatography was carried out either by hand-made columns with Merck Silica 60Ǻ or using a Biotage Isolera™ One flash purification system using a wet-loading Biotage SNAP cartridge, collecting fractions using by UV detection at 254 nm when appropriate. Lab distilled water was used unless otherwise stated.

The purity of the compound was determined using NMR spectroscopy, accurate mass spectrometry and LC-MS analysis. All NMR chemical shifts are quoted using tetramethylsilane (TMS) as a reference of ($\delta_{C/H}$ = 0). ^{1}H and ^{13}C NMR were both performed on a Bruker AV-400 spectrometer. For ^{1}H NMR, the residual solvent peak used as an internal reference was $CDCl_3$ (δ_H = 7.26 ppm), CD_3OD (δ_H = 3.31 ppm) or DMSO-d_6 (δ_H = 2.50 ppm) and chemical shifts are reported as: (multiplicity, coupling constant *J* (Hz), number of protons). For ^{13}C NMR, the residual solvent peak used as an internal reference was $CDCl_3$ (δ_C = 77.2 ppm), CD_3OD (δ_C = 49.0 ppm) or DMSO-d_6 (δ_C = 39.5 ppm).

Mass spectrometry was performed using chemical ionisation (CI), electron ionisation (EI) or electrospray ionisation (ESI) on an AUTOSPEC P673 spectrometer by the Chemistry Department Mass Spectrometry Service at Imperial College London. LC-MS analysis and purification were carried out on a Waters HPLC system equipped with a 2767 autosampler, a 515 pump, a 3100 mass spectrometer with ESI, and a 2998 Photodiode Array Detector (detection at 200-600 nm). The system was fitted with Waters XBridge C18 columns (4.6 mm × 100 mm for analytical and 19 mm × 100 mm for preparative LC/MS). The flow rate was 1.2 mL/min for analytical and 20 mL/min for preparative LC-MS, with an 18 min runtime. A gradient of methanol and water, both containing 0.1% of formic acid, was used as the mobile phase.

Syntheses of the AzTB, AzT and AzRB capture reagents were as previously reported.[2–4]

3.2 Synthesis of Probe 1

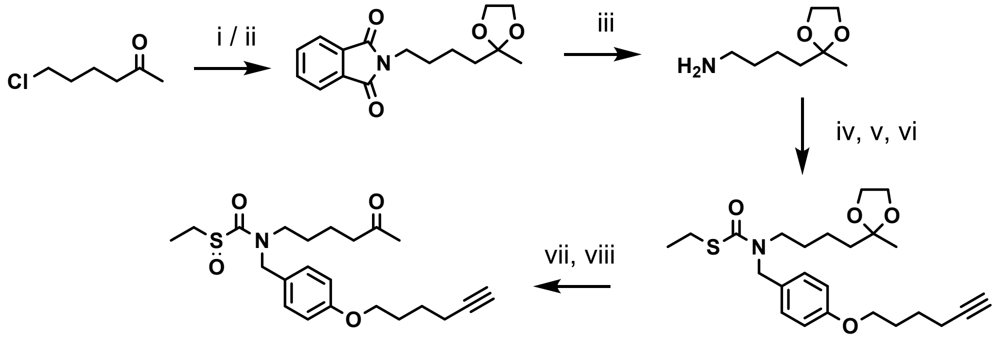

Sulforaphane Probe 1

Scheme S1. Synthesis of Probe **1**. Reagents and conditions: i) *p*-toluenesulfonic acid, ethylene glycol, toluene, 135 °C; ii) phthalimide, K_2CO_3, KI, DMF; iii) NH_2NH_2, EtOH; iv) 4-hex-5-ynyloxy-benzaldehyde, MeOH; v) $NaBH_4$, MeOH; vi) *S*-ethyl chlorothiolformate, DIPEA, DCM, 0 °C; vii) 2 M HCl, THF; viii) *m*-CPBA, DCM, -78 °C.

3.2.1 4-Hex-5-ynyloxy-benzaldehyde

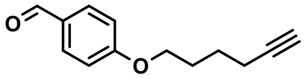

4-hydroxybenzaldehyde (2.0 g, 16.5 mmol) was dissolved in DMF (30 mL). K_2CO_3 (4.5 g, 32.8 mmol) and potassium iodide (1.4 g, 8.2 mmol) were added to form a white suspension. 6-chloro-hex-1-yne (2.7 mL, 22.3 mmol) was added and the resultant light brown suspension stirred at 80 °C for 5 h, and then at room temperature for 2 h. The suspension was diluted with EtOAc and the organic layer washed with water and with brine, dried over sodium sulphate, filtered and concentrated under vacuum to yield a brown oil. The crude product was recrystallized from hexane, filtered under vacuum and washed with ice cold hexane to yield a pale yellow solid (2.7 g, 81%). ^{1}H NMR: δ_H/ppm (400 MHz, $CDCl_3$) 9.91 (s, 1H), 7.86 (m, 2H), 7.02 (m, 2H), 4.10 (t, J = 6.3 Hz, 2H), 2.32 (td, J = 2.6, 7.0 Hz, 2H), 1.98 (m, 3H), 1.77 (m, 2H). ^{13}C NMR: δ_C/ppm (101 MHz, $CDCl_3$) 190.9, 164.1, 132.02, 129.9, 114.7, 83.9, 68.8, 67.7, 28.1, 24.9, 18.1. ES+ HRMS: found 203.1068 ($C_{13}H_{15}O_2$, $[M+H]^+$, requires 203.1072).

3.2.2 2-(4-Chloro-butyl)-2-methyl-[1,3]dioxolane

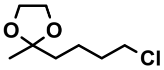

To 6-chloro-2-hexanone (1.0 mL, 7.6 mmol) in toluene (50 mL) was added *p*-toluenesulfonic acid (77 mg, 0.40 mmol) and ethylene glycol (0.85 mL, 15.2 mmol). The suspension was fitted with Dean Stark apparatus and stirred at 160 °C for 4 h. The clear solution was diluted with EtOAc and washed with saturated sodium bicarbonate and brine. The organic layer was dried over sodium sulphate, filtered and concentrated under vacuum to yield a yellow/brown liquid. The crude product was purified by automated silica chromatography eluting in a gradient of hexane/EtOAc to yield a colourless liquid (1.0 g, 75%). ^{1}H NMR: δ_H/ppm (400 MHz, $CDCl_3$) 3.93 (m, 4H), 3.53 (t, J = 6.7 Hz, 2H), 1.79 (m, 2H), 1.66 (m, 2H), 1.55 (m, 2H), 1.31 (s, 3H). ^{13}C NMR: δ_C/ppm (101 MHz, $CDCl_3$) 110.0, 64.8, 45.1, 38.5, 32.8, 23.9, 21.6. ES+ HRMS: found 179.0843 ($C_8H_{16}O_2Cl$, $[M+H]^+$, requires 179.0839).

3.2.3 2-(4-Phthalimido-butyl)-2-methyl-[1,3]dioxolane

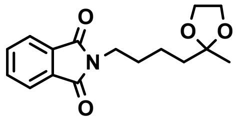

To a suspension of 2-(4-chloro-butyl)-2-methyl-[1,3]dioxolane (320 mg, 1.8 mmol), K_2CO_3 (620 mg, 4.5 mmol) and potassium iodide (16 mg, 0.10 mmol) in DMF (4 mL) was added a solution of phthalimide (310 mg, 2.2 mmol) in DMF (4 mL). The pale yellow suspension was stirred at 80 °C for 17 h, during which time the colour of the suspension deepened to a strong yellow and a pale yellow residue formed. The suspension was cooled to room temperature and diluted with water. The organic layer was extracted with diethyl ether. The organic layers were combined, dried over sodium sulphate, filtered and concentrated under vacuum to yield a pale yellow oil which solidified upon standing. The crude product was purified by flash chromatography, eluting with a hexane/EtOAc gradient to yield a white solid (472 mg, 91%).^{1}H NMR: δ_H/ppm (400 MHz, $CDCl_3$) 7.86 (dd, J = 3.0, 5.4 Hz, 2H), 7.73 (dd, J = 3.0, 5.4 Hz, 2H), 3.95 (m, 4H), 3.71 (m, 2H), 1.70 (m, 4H), 1.47 (m, 2H), 1.33 (s, 3H). ^{13}C NMR: δ_C/ppm (101 MHz, $CDCl_3$) 168.5, 134.3, 133.9, 132.2, 123.6, 123.2, 110.0, 109.9,

64.7, 38.6, 37.9, 28.7, 23.8, 21.4. ES+ HRMS: found 290.1376 ($C_{16}H_{20}NO_4$, $[M+H]^+$, requires 290.1392).

3.2.4 2-(4-Amino-butyl)-2-methyl-[1,3]dioxolane

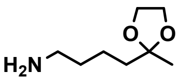

To a solution of 2-(4-phthalimido-butyl)-2-methyl-[1,3]dioxolane (471 mg, 1.4 mmol) in ethanol (20 mL) was added hydrazine monohydrate (250 µL, 5.0 mmol). The solution was refluxed at 80 °C for 3 h, during which time a white precipitate was formed. The suspension was cooled to room temperature and the precipitate isolated by filtration. The filtrate was concentrated under vacuum to yield a white residue which was re-dissolved in ethanol, filtered and concentrated under vacuum. The residue was dissolved in DCM, filtered and concentrated to yield a yellow solid (252 mg) which was used without further purification. ^1H NMR: δ_H/ppm (400 MHz, $CDCl_3$) 3.83 (m, 4H), 2.56 (t, *J* = 6.5 Hz, 2H), 1.53 (m, 2H), 1.34 (m, 4H), 1.21 (s, 3H). ES+ HRMS: found 160.1332 ($C_8H_{18}NO_2$, $[M+H]^+$, requires 160.1338).

3.2.5 (4-hex-5-ynyloxy-benzyl)-[4-(2-methyl-[1,3]dioxolan-2-yl)-butyl]-amine

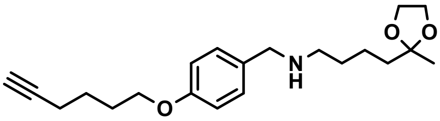

To a suspension of 4-hex-5-ynyloxy-benzaldehyde (204 mg, 1.0 mmol) and sodium sulphate (2.5 g) in dry methanol (4 mL) was added a solution of 2-(4-amino-butyl)-2-methyl-[1,3]dioxolane (108 mg, 0.68 mmol) in dry methanol (2 mL). The suspension was stirred under nitrogen at room temperature for 20 h. The suspension was subsequently cooled to 0 °C and sodium borohydride (51 mg, 1.3 mmol) added. The suspension was stirred at 0 °C for 30 min. Water (40 mL) was added, the methanol removed under vacuum, and the remaining aqueous suspension was extracted with DCM. The organic layers were combined, dried over sodium sulphate and concentrated under vacuum to yield a yellow oil. The crude product was purified by automated silica chromatography with a gradient of 0-20% methanol/1% triethylamine in DCM/1% triethylamine to yield a yellow solid (107 mg, 46%).^1H NMR: δ_H/ppm (400 MHz, $CDCl_3$) 7.28 (d, *J* = 8.2 Hz, 2H), 6.87 (d, *J* = 8.6 Hz, 2H), 3.98 (t, *J* = 6.3 Hz, 2H), 3.93 (m, 4H), 3.77 (s, 2H), 2.65 (m, 2H), 2.29 (td, *J* = 2.6, 7.0 Hz, 2H), 1.99 (t, *J* = 2.6 Hz, 1H), 1.92 (m, 2H), 1.74 (m, 2H), 1.61 (m, 4H), 1.44 (m, 2H), 1.30 (d, *J* = 13.9 Hz, 3H) ^{13}C NMR: δ_C/ppm (101 MHz, $CDCl_3$) 158.3, 130.7, 129.7, 114.4, 110.0, 84.1, 68.7, 67.3, 64.6, 52.9, 48.6, 39.0, 29.5, 28.3, 25.0, 23.8, 21.8, 18.2. ES+ HRMS: found 346.2369 ($C_{21}H_{32}NO_3$, $[M+H]^+$, requires 346.2382).

3.2.6 *S*-Ethyl (4-hex-5-ynyloxy-benzyl)-[4-(2-methyl-[1,3]dioxolan-2-yl)-butyl]-thiocarbamate

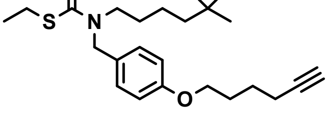

A solution of (4-hex-5-ynyloxy-benzyl)-[4-(2-methyl-[1,3]dioxolan-2-yl)-butyl]-amine (103 mg, 0.30 mmol) in dry DCM (10 mL) was cooled to 0 °C. DIPEA (160 µL, 0.90 mmol) was added dropwise, followed by *S*-ethyl chlorothiolformate (80 µL, 0.75 mmol). The solution was stirred

at 0 °C under nitrogen for 45 min. The reaction mixture was diluted with EtOAc and the organic layer washed with 1 M HCl, saturated sodium bicarbonate, and brine. The organic layer was dried over sodium sulphate, filtered and concentrated under vacuum. The crude residue was purified by automated silica chromatograph with a hexane/EtOAc gradient to yield a colourless oil (87 mg, 67%).^{1}H NMR: δ_H/ppm (400 MHz, $CDCl_3$) 7.18 (d, *J* = 8.5 Hz, 2H), 6.86 (d, *J* = 8.1 Hz, 2H), 4.52 (br d, *J* = 23.5 Hz, 2H), 3.99 (t, *J* = 6.2 Hz, 2H), 3.91 (m, 4H), 3.27 (br d, *J* = 32.7 Hz, 2H), 2.96 (q, *J* = 7.4 Hz, 2H), 2.30 (td, *J* = 2.6, 7.0 Hz, 2H), 1.99 (t, *J* = 2.6 Hz, 1H), 1.92 (m, 2H), 1.64 (m, 2H), 1.65 (m, 2H), 1.55 (m, 2H) 1.38 (m, 2H), 1.34 (t, J = 7.4 Hz, 3H), 1.31 (s, 3H). ^{13}C NMR: δ_C/ppm (101 MHz, $CDCl_3$) 168.5, 158.5, 129.3, 128.6, 114.5, 110.0, 84.1, 68.7, 67.3, 64.7, 50.7, 50.6, 49.3, 49.2, 47.0, 46.8, 46.7, 38.8, 28.3, 27.89, 27.5, 25.0, 24.9, 23.8, 21.4, 18.2, 15.4. ES+ HRMS: found 434.2357 ($C_{24}H_{36}NO_4S$, $[M+H]^+$, requires 434.2365).

3.2.7 *S*-Ethyl (4-hex-5-ynyloxy-benzyl)-(5-oxo-hexyl)-thiocarbamate

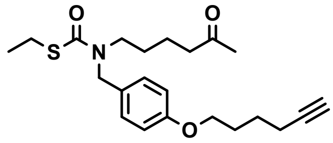

To a solution of *S*-Ethyl (4-hex-5-ynyloxy-benzyl)-[4-(2-methyl-[1,3]dioxolan-2-yl)-butyl]-thiocarbamate (86 mg, 0.20 mmol) in THF (5.0 mL) was added 2 M HCl (1.0 mL) dropwise. The solution was stirred at room temperature for 3 h, then diluted with EtOAc and washed with distilled water, saturated sodium bicarbonate and brine. The organic layer was dried over sodium sulfate, filtered and concentrated under vacuum. The crude residue was purified by automated silica chromatography, eluting in a gradient of hexane/EtOAc to yield a colourless oil (67 mg, 85%). ^{1}H NMR: δ_H/ppm (400 MHz, $CDCl_3$) 7.17 (d, *J* = 8.5 Hz, 2H), 6.86 (d, *J* = 8.2 Hz, 2H), 4.52 (br d, *J* = 18.8 Hz, 2H), 3.99 (t, *J* = 6.2 Hz, 2H), 3.27 (br d, *J* = 30.8 Hz, 2H), 2.96 (q, *J* = 7.4 Hz, 2H), 2.45 (t, *J* = 6.7 Hz, 2H), 2.29 (td, *J* = 2.6, 7.0 Hz, 2H), 2.14 (s, 3H), 1.99 (t, *J* = 2.6 Hz, 1H), 1.92 (m, 2H), 1.74 (m, 2H), 1.54 (br s, 4H), 1.32 (t, *J* = 7.4 Hz, 3H). ^{13}C NMR: δ_C/ppm (101 MHz, $CDCl_3$) 168.4, 158.5, 129.3, 129.0, 128.6, 114.6, 84.1, 68.7, 67.3, 50.7, 49.3, 49.2, 46.6, 46.5, 46.4, 46.2, 46.2, 43.0, 30.0, 28.3, 27.2, 26.9, 26.8, 26.7, 25.0, 24.9, 20.8, 18.2, 15.4. ES+ HRMS: found 390.2094 ($C_{22}H_{32}NO_3S$, $[M+H]^+$, requires 390.2103).

3.2.8 *S*-Ethyl(4-hex-5-ynyloxy-benzyl)-(5-oxo-hexyl)-thiocarbamatesulfoxide (1)

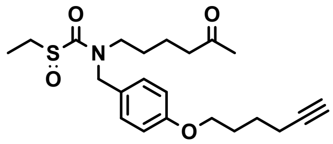

A solution of *S*-ethyl(4-hex-5-ynyloxy-benzyl)-(5-oxo-hexyl)-thiocarbamate (66 mg, 0.17 mmol) in DCM (2.0 mL) was cooled to -78 °C and 3-chloroperbenzoic acid (37 mg, 0.16 mmol) in DCM (1.0 mL) was added dropwise. The reaction stirred at -78 °C for 50 min. The solvent was removed under vacuum and the solid residue taken up in EtOAc, and washed with saturated sodium bicarbonate and brine. The organic layer was dried over sodium sulphate, filtered and concentrated under vacuum. The crude residue was purified by automated column chromatography, eluting in a gradient of hexane/EtOAc to yield **1** as a colourless oil (48 mg, 69%). ^{1}H NMR: δ_H/ppm (400 MHz, $CDCl_3$) 7.5 – 7.25 (m, 2H), 6.83 – 6.95 (m, 2H), 4.48 – 4.86 (m, 2H), 4.00 (td, J = 6.2, 2.2 Hz, 2H), 3.29 – 3.62 (m, 2H), 2.90 –

3.14 (m, 2H), 2.2 – 2.52 (m, 2H), 2.30 (td, J = 7.0, 2.6 Hz, 2H), 2.15 (d, J = 3.7 Hz, 3H), 2.00 (t, J = 2.7 Hz, 1H), 1.88 – 1.97 (m, 2H), 1.70 – 1.81 (m, 2H), 1.48 – 1.70 (m, 4H), 1.40 (td, J = 7.5, 3.2 Hz, 3H). ^{13}C NMR: δ_C/ppm (101 MHz, $CDCl_3$) 208.3, 208.1, 168.5, 168.4, 159.1, 158.9, 129.9, 128.9, 127.5, 126.8, 114.9, 114.8, 84.0, 77.4, 77.0, 76.7, 68.7, 67.4, 67.4, 50.3, 48.7, 47.2, 45.5, 45.4, 44.7, 42.7, 42.6, 30.0, 28.2, 28.1, 26.1, 25.0, 20.6, 20.4, 18.2, 7.0, 7.0. ES+ HRMS: found 428.1856 ($C_{22}H_{31}NO_4SNa$, $[M+Na]^+$, requires 428.1872).

3.3 Synthesis of Probe 2

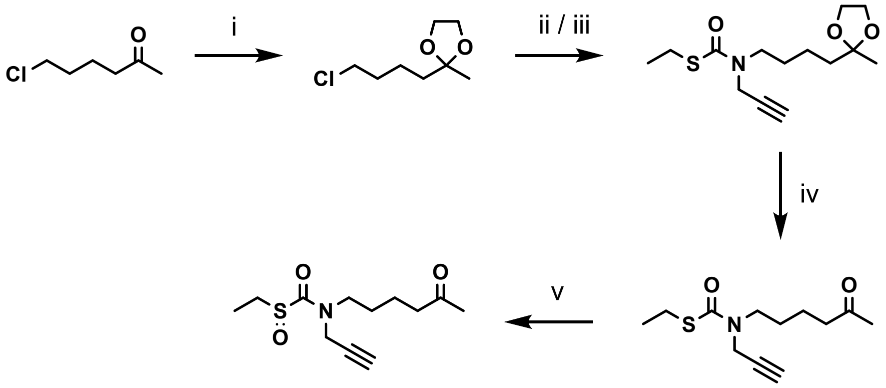

Sulforaphane Probe 2

Scheme S2. Synthesis of Probe **2**. Reagents and conditions: i) *p*-toluenesulfonic acid, ethylene glycol, toluene, 135 °C; ii) propargyl amine, K_2CO_3, KI, DMF; iii) DIPEA, *S*-ethyl chlorothiolformate, DCM, 0 °C; iv) 2 M HCl, THF; v) *m*-CPBA, DCM, -78 °C.

3.3.1 *S*-Ethyl (prop-2-ynyl)-[4-(2-methyl-[1,3]dioxolan-2-yl)-butyl]-thiocarbamate

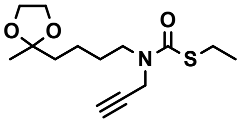

To a suspension of propargyl amine (170 µL, 2.6 mmol), K_2CO_3 (304 mg, 2.2 mmol) and potassium iodide (308 mg, 1.9 mmol) in DMF (6 mL) was added a solution of 2-(4-chloro-butyl)-2-methyl-[1,3]dioxolane (303 mg, 1.7 mmol) in DMF (1 mL). The orange suspension was stirred at 70 °C overnight and at room temperature for a further 48 h then diluted with EtOAc and washed with brine. The organic layer was dried over sodium sulfate, filtered and concentrated under vacuum to yield a brown oil which was dissolved in DCM (10 mL) and cooled to 0 °C. DIPEA (890 µL, 5.1 mmol) was added dropwise, followed by *S*-ethyl chlorothiolformate (420 µL, 4.0 mmol) and the solution stirred at 0 °C for 2 h. The solution was diluted with DCM and washed with 1 M HCl, saturated sodium bicarbonate, and brine. The organic layer was dried over magnesium sulphate, filtered and concentrated under vacuum to yield an orange liquid which was purified by automated silica chromatography eluting in a gradient of hexane/EtOAc to yield a yellow oil (184 mg, 38%). ^1H NMR: δ_H/ppm (400 MHz, $CDCl_3$) 4.19 (s, 2H), 3.93 (m, 4H), 3.43 (t, *J* = 7.3 Hz, 2H), 2.92 (q, *J* = 7.3 Hz, 2H), 2.23 (s, 1H), 1.65 (m, 4H), 1.42 (m, 2H), 1.28 (m, 6H). ^{13}C NMR: δ_C/ppm (101 MHz, $CDCl_3$) 110.0, 64.8, 47.4, 38.9, 28.0, 25.0, 23.9, 21.4, 15.4. ES+ HRMS: found 286.1478 ($C_{14}H_{24}NO_3S$, $[M+H]^+$, requires 286.1477).

3.3.2 *S*-Ethyl (prop-2-ynyl)-(5-oxo-hexyl)-thiocarbamate

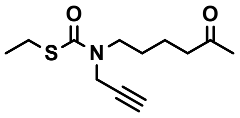

To a solution of *S*-ethyl-(prop-2-ynyl)-[4-(2-methyl-[1,3]dioxolan-2-yl)-butyl]-thiocarbamate (180 mg, 0.63 mmol) in THF (10.4 mL) was added 2 M HCl (2.6 mL) dropwise. The colourless solution was stirred at room temperature for 3 h, then diluted with EtOAc and washed with distilled water, saturated sodium bicarbonate and brine. The organic layer was dried over sodium sulphate, filtered and concentrated under vacuum. The crude residue was purified by automated silica chromatography eluting in a gradient of hexane/EtOAc to yield a colourless oil (87 mg, 68%). ^{1}H NMR: δ_H/ppm (400 MHz, $CDCl_3$) 4.18 (s, 2H), 3.44 (s, 2H), 2.92 (q, *J* = 7.4 Hz, 2H), 2.48 (t, *J* = 6.7 Hz, 2H), 2.25 (s, 1H), 2.14 (s, 3H), 1.60 (m, 4H), 1.28 (t, *J* = 7.4 Hz, 3H). ^{13}C NMR: δ_C/ppm (101 MHz, $CDCl_3$) 208.4, 208.4, 168.4, 78.5, 72.2, 47.0, 43.0, 30.0, 27.2, 25.0, 20.7, 15.2. ES+ HRMS**:** found 242.1203 ($C_{12}H_{20}NO_2S$, $[M+H]^+$, requires 242.1215).

3.3.3 *S*-Ethyl (prop-2-ynyl)-(5-oxo-hexyl)-thiocarbamate sulfoxide (2)

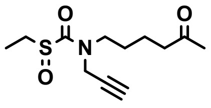

A solution of *S*-ethyl-(prop-2-ynyl)-(5-oxo-hexyl)-thiocarbamate (74 mg, 0.31 mmol) in DCM (2.5 mL) was cooled to -78 °C and 3-chloroperbenzoic acid (68 mg, 0.30 mmol) in DCM (1.5 mL) was added dropwise. The reaction was stirred at -78 °C for 50 min. The solvent was removed under vacuum and the solid residue taken up in EtOAc, washed with saturated sodium bicarbonate and brine. The organic layer was dried over sodium sulphate, filtered and concentrated under vacuum. The crude residue was purified by automated silica chromatography eluting in a gradient of hexane/EtOAc, to yield **2** as a colourless oil (12 mg, 15%). ^{1}H NMR: δ_H/ppm (400 MHz, $CDCl_3$) 4.53 (qd, *J* = 2.5, 18.3 Hz, 1H), 4.24 (m, 1H), 3.82 (m, 0.5H), 3.67 (dd, *J* = 7.2, 14.6 Hz, 0.5H), 3.54 (td, *J* = 3.1, 7.0 Hz, 1H), 3.07 (m, 2H), 2.50 (q, *J* = 6.5 Hz, 2H), 2.34 (dt, *J* = 2.5, 24.1 Hz, 1H), 2.14 (d, *J* = 2.3 Hz, 3H), 1.63 (m, 5H), 1.39 (dt, *J* = 2.5, 8.9 Hz, 3H). ^{13}C NMR: δ_C/ppm (101 MHz, $CDCl_3$) 208.3, 168.2, 73.9, 73.5, 48.2, 45.8, 45.7, 45.4, 42.7, 42.6, 36.7, 35.2, 30.0, 27.9, 26.1, 20.5, 20.3, 7.0, 6.9.. ES+ HRMS: found 280.0977 ($C_{12}H_{20}NO_3S$, $[M+Na]^+$, requires 280.0983).

3.4 ^{1}H NMR, ^{13}C NMR, HRMS and LC-MS data

Data for **1** are shown below:

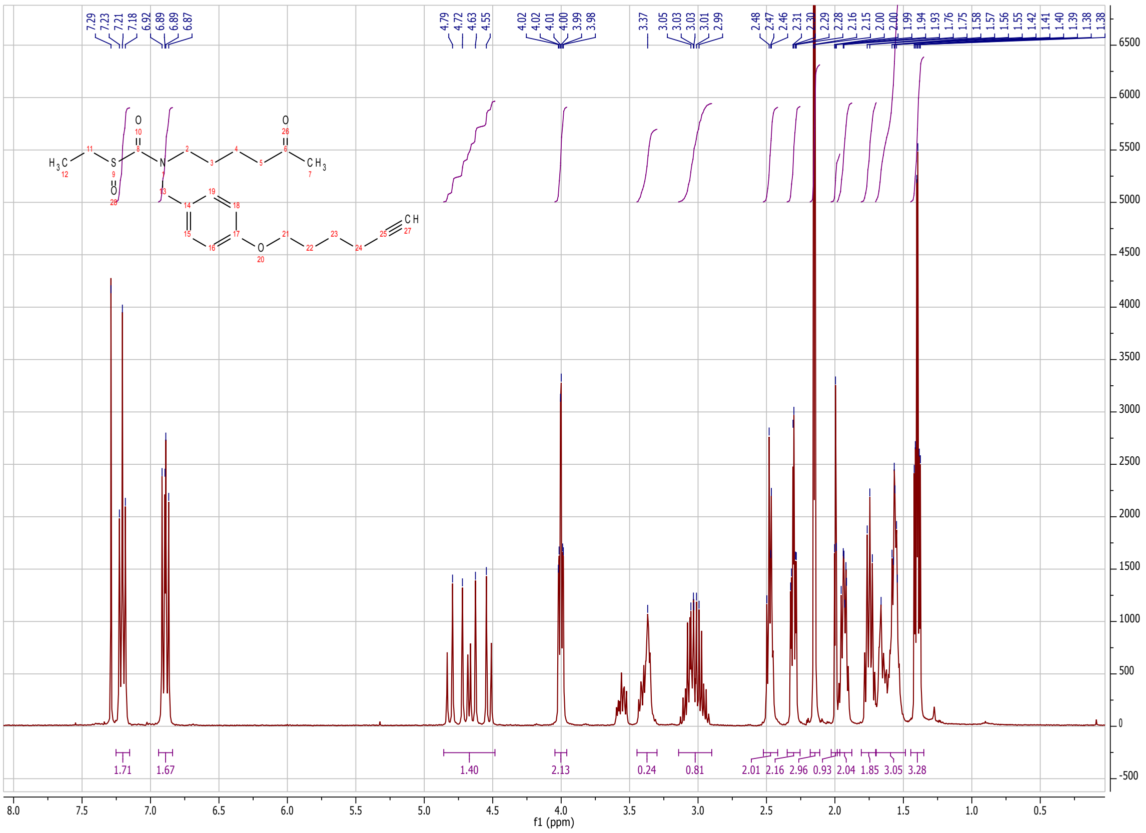

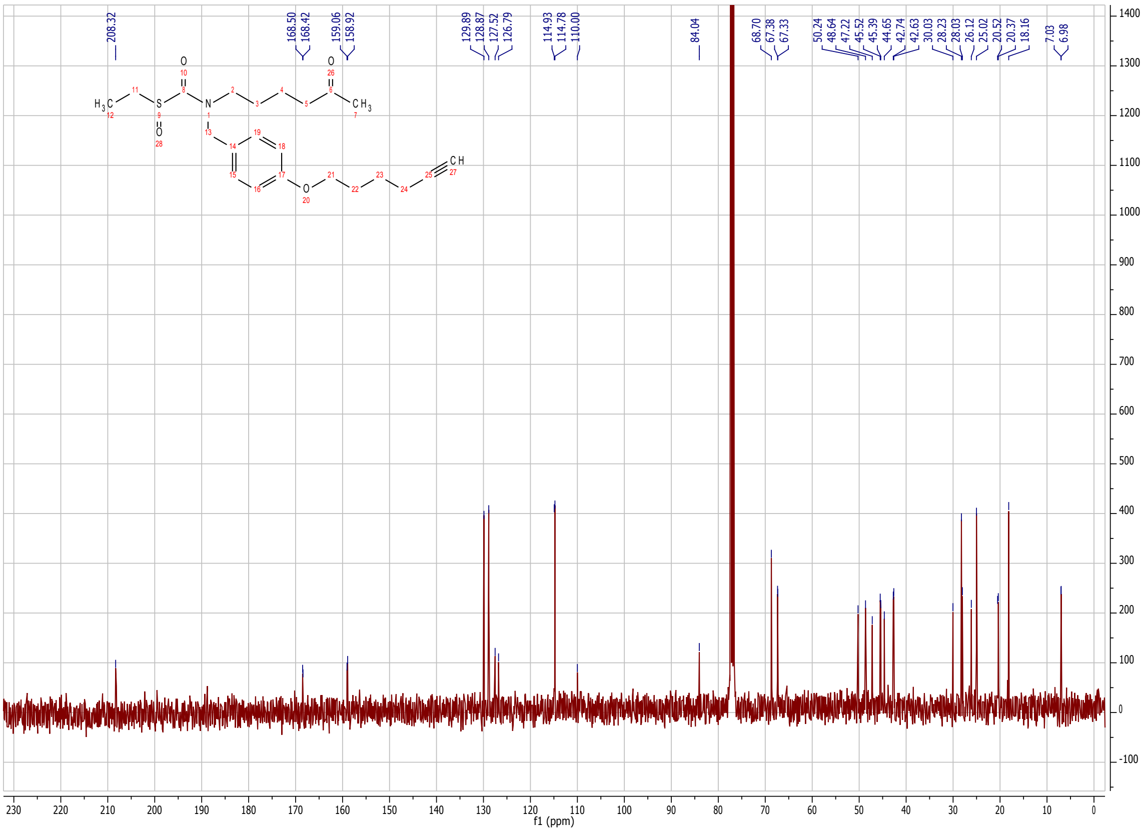

(A)

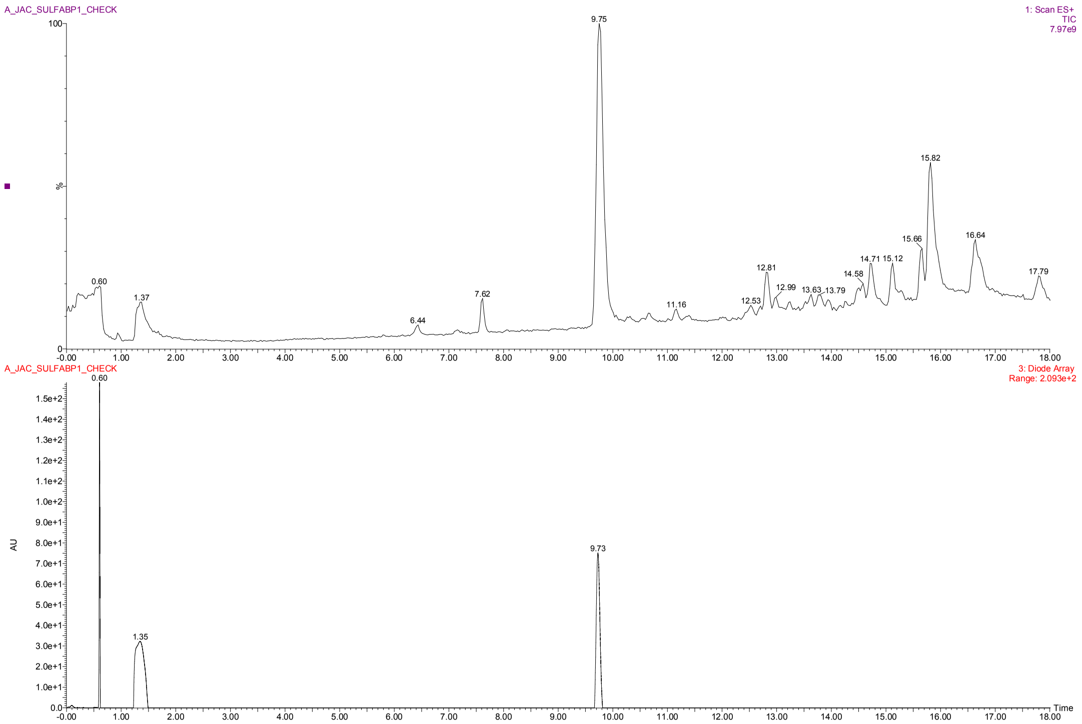
A_JAC_SULFABP1_CHECK
1: Scan ES+
TIC
7.97e9
9.75
15.82
16.64
15.66
14.71 15.12
14.58
12.81
12.99
13.63 13.79
12.53
11.16
17.79
7.62
6.44
0.60
1.37
A_JAC_SULFABP1_CHECK
3: Diode Array
Range: 2.093e+2
0.60
1.35
9.73
AU
Time

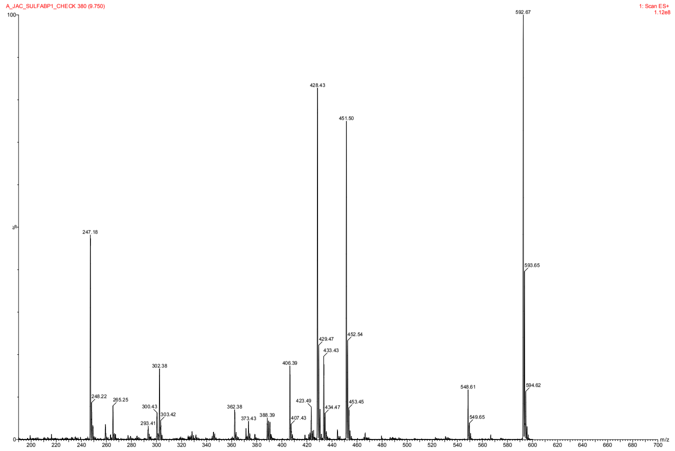
A_JAC_SULFABP1_CHECK 380 (9.750)
1: Scan ES+
1.12e8
592.67
428.43
451.50
247.18
593.65
452.54
429.47
433.43
302.38
406.39
594.62
548.61
m/z

Data for **2** are shown below

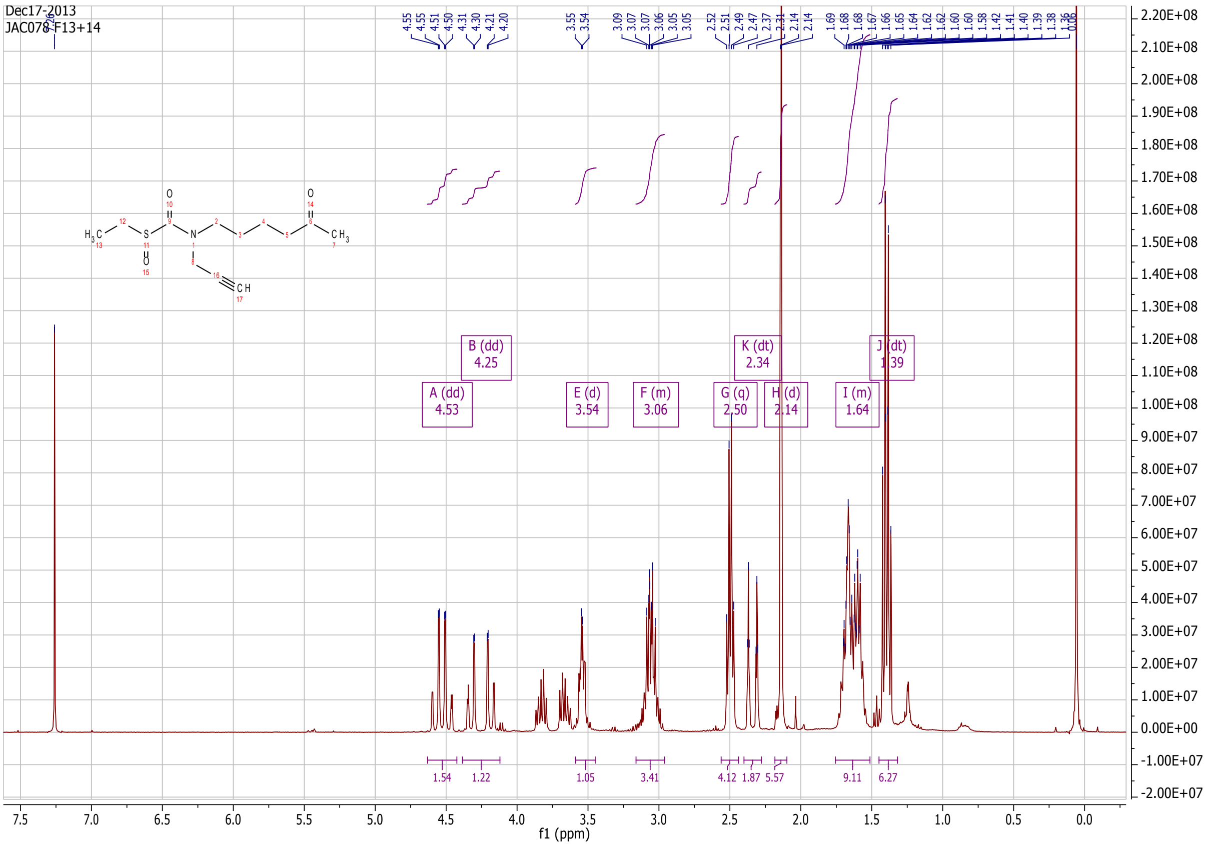

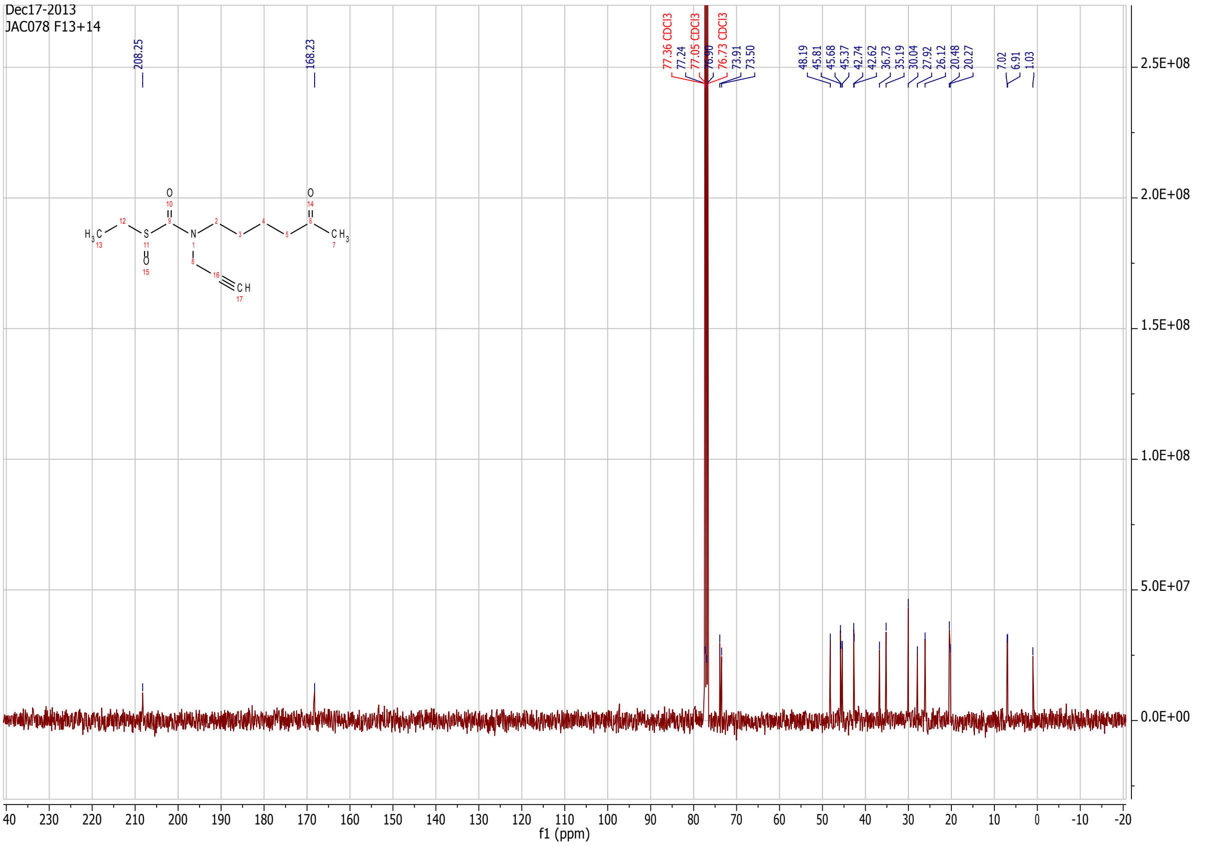

(B)

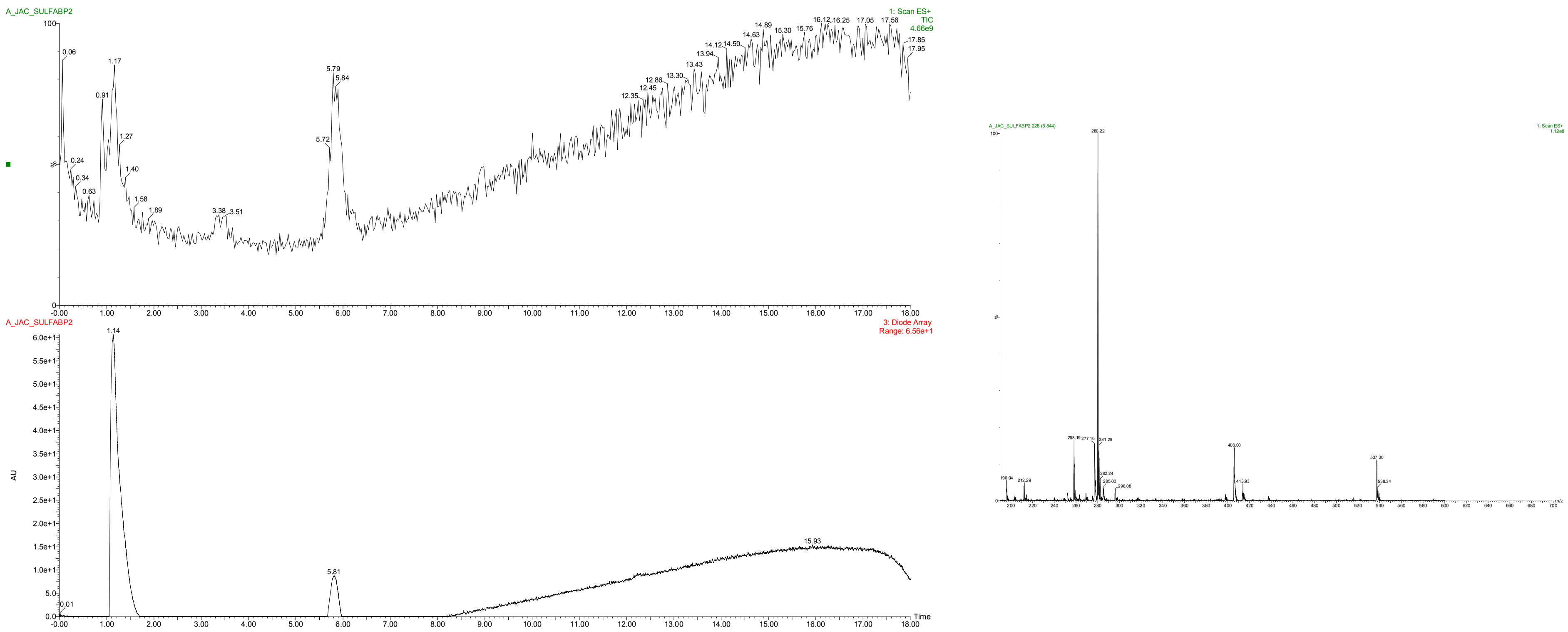
A_JAC_SULFABP2
1: Scan ES+
TIC
4.66e9
A_JAC_SULFABP2
3: Diode Array
Range: 6.56e+1
AU
Time
A_JAC_SULFABP2 228 (5.844)
1: Scan ES+
1.12e8
280.22
406.00
537.30
m/z

4 Biological and biochemical methods

4.1 General methods

Ultrapure water was obtained using a MilliQ® Millipore purification system. In-gel fluorescence was recorded using an ETTAN DIGE Imager (GE Healthcare) and chemiluminescence was recorded using a LAS-4000 Imaging System (GE Healthcare). Absorbance in 96-well plates was measured using a SpectraMax M2e Microplate Reader (Molecular Devices). Protein concentration was determined using the BioRad DC Protein Assay following the manufacturer's instructions, measuring absorbance at 750 nm, using BSA as a protein standard.

All biological and chemical reagents were purchased from Sigma-Aldrich unless otherwise specified. *N*-ethylmaleimide (NEM), iodoacetamide (IA), and D,L-sulforaphane (Toronto Research Chemicals, Canada) were purchased and used without further purification. All compounds were prepared as DMSO stocks for biological experiments unless otherwise stated, stored at -20 ˚C and thawed on the day of use, except for IA that was prepared fresh on the day of use.

4.2 Cell culture

MDA-MB-231 and MCF7 cell lines were obtained from CRUK cell services core facility and were cultured in DMEM supplemented with 10% (v/v) FBS, incubated at 37 °C in a 10% CO_2 humidified incubator. Cells were grown in 10 cm or 6 cm cell culture plates (Falcon or Corning). Cells were detached with trypsin (0.2%) during passaging. Quantitative proteomics 'spike-in' samples were grown in R10K8 DMEM media (Dundee Cell Products) supplemented with 10% (v/v) dialysed FBS for > 7 passages to ensure incorporation of the R10K8 label before proceeding to further experiments. Incorporation of R10K8 labelling was determined to be > 97% by mass spectrometry in accordance with guidelines for SILAC-based applications.[5] For R10K8 labelled cells, enzyme-free, PBS-based cell dissociation buffer (Gibco Life technologies) was used instead of trypsin for cell detachment during passaging. All described experiments were carried out with cells at low passage number (< 25) and which were generally plated 24 or 48 h prior to treatment. All cells were grown to 70-90% confluence prior to commencing an experiment.

4.3 In-cell competition-based assays for in-gel fluorescence and Western Blot analyses

4.3.1 Compound treatment of cells and cell lysis

Cells were incubated with D,L-sulforaphane or competition compounds at a fixed concentration in the cell media for 30 min (0.2% DMSO final). Media was then aspirated and replaced with fresh media containing fixed concentrations of D,L-sulforaphane or competition compounds and **1** or **2** (0.2% DMSO final). Cells were incubated for a further 30 min, after which the media was aspirated, cells washed three times with PBS, and lysed on the plate with whole cell lysis buffer (1% NP-40, 1% sodium deoxycholate, 0.1% SDS, 150 mM NaCl, PBS pH 7.6, 1 x Complete EDTA-free protease inhibitor (Roche Diagnostics)). The lysates were transferred to microcentrifuge tubes and incubated on ice for 15 min, followed by centrifugation (17,000 × g, 20 min, 4 ˚C) to remove insoluble cellular debris. Lysates were stored at -20 ˚C until further use.

4.3.2 Copper catalysed azide-alkyne cycloaddition (CuAAC)

Protein lysates were thawed on ice and made up to 100 µL at a concentration of 1 mg/mL with lysis buffer. A click reaction master mix was prepared freshly as follows: capture reagent (either AzT or AzTB, 1 µL, 10 mM DMSO stock concentration; 0.1 mM final concentration), $CuSO_4$ (2 µL, 50 mM stock concentration; 1 mM final concentration), TCEP (2 µL, 50 mM stock concentration; 1 mM final concentration) and TBTA (1 µL, 10 mM DMSO stock concentration; 0.1 mM final concentration). 6 µL of this master mix was added to each sample and the sample vortexed for 1 h at room temperature. The reaction was quenched by addition of EDTA (final concentration 10 mM) and proteins precipitated by addition of methanol (4 vol.), chloroform (1 vol.) and water (3 vol.), followed by centrifugation (17,000 × g, 2 min). The upper liquid phase was discarded before addition of methanol (4 vol.) and centrifugation (17,000 × g, 2 min). The resulting protein pellet was washed twice with methanol (8 vol.) and air dried for 10 min.

4.3.3 Gel electrophoresis and in-gel fluorescence

For in-gel fluorescence analysis, the protein pellet was re-suspended in 2% SDS in PBS (10 µL), 100 mM EDTA (10 µL), PBS (40 µL) and 4 × NuPAGE® LDS sample loading buffer containing 5% (v/v) 2-mercaptoethanol (20 µL) to give a final concentration of protein of 1.25 mg/mL. Samples were then heated at 90 °C for 5 min. SDS-PAGE analysis was performed with 12% acrylamide Bis-Tris gels, using a BioRad Mini-PROTEAN® Tetra Cell system with MOPS running buffer (50 mM MOPS pH 7.7, 50 mM Tris Base, 0.1% SDS, 1 mM EDTA), with Precision Plus Protein All Blue Standard (BioRad) used as molecular weight marker. Gels were fixed in gel soaking solution (50% water, 40% MeOH, 10% acetic acid) for 30 min and washed twice with MilliQ water before in-gel fluorescence visualisation (excitation wavelength 552 nm, emission wavelength 570 nm). Further data analysis was performed with ImageQuant™ TL software. In-gel protein levels were determined by Coomassie staining (9.2% phosphoric acid, 10% ammonium sulfate, 0.12% Coomassie brilliant blue G-250 dye, 20% methanol in water).

4.3.4 Affinity enrichment and Western Blot analysis

Protein lysates (0.6 mg) were prepared at a concentration of 2 mg/mL and functionalised with AzRB click reaction master mix (18 µL) for 1 h before being quenched and protein precipitated as described previously. The protein pellet was re-suspended in 2% SDS in PBS (60 µL), 100 mM EDTA (60 µL), 100 mM DTT (6 µL), 1 × Complete EDTA-free protease inhibitor, and PBS (324 µL) to give a final volume 450 µL. 75 µL of the re-suspended protein pellet was then added to 4 × NuPAGE® LDS sample loading buffer containing 5% (v/v) 2-mercaptoethanol (25 µL). Samples were heated at 90 ˚C for 5 min. These samples were designated as the pre-pull down lysate (L). To the remaining 375 µL of lysate, PBS was added (75 µL). Neutravidin sepharose resin (Thermo Scientific) was washed 3 × with 0.2% SDS in PBS before resin slurry (75 µL) was added to each sample to give a final volume of 525 µL at 1 mg/mL protein concentration (0.2% SDS final). Samples were then shaken at room temperature for 2 h. The supernatant was removed and a 75 µL aliquot taken and added to 4 × NuPAGE® LDS sample loading buffer containing 5% (v/v) 2-mercaptoethanol (25 µL). Samples were heated at 90 ˚C for 5 min. These samples were designated as the supernatant samples (SN). The Neutravidin sepharose resin from each sample was then washed with 4 × 400 µL 0.2% SDS in PBS. 2% SDS in PBS (75 µL) was then added to each sample which were heated at 90 ˚C for 15 min. 4 × NuPAGE® LDS sample loading buffer containing 5% (v/v) 2-mercaptoethanol (25 µL) was added and the samples heated at 90 ˚C

for a further 5 min. These samples were designated as the pull-down samples (PD). Samples were then loaded onto SDS-PAGE gels for analysis; L 12 µL (10 µg protein), SN 15 µL (10 µg protein) and PD 15 µL.

After electrophoresis, proteins were transferred from non-fixed gels to PVDF membranes using an iBlot® Gel Transfer Device (Invitrogen, Life Technologies) according to the manufacturer's guidelines. After transfer, membranes were blocked in 5% (w/v) dried skimmed milk in TBS plus 0.1% (v/v) Tween-20 (TBST) for 2 h at room temperature and incubated with the appropriate primary antibody in blocking solution with gentle agitation overnight at 4 ˚C (anti-STAT3 (Santa Cruz Biotechnology, sc-482, rabbit polyclonal, dilution 1:100), anti-STAT1 p84/p91 (Santa Cruz Biotechnology, sc-346, rabbit polyclonal, dilution 1:100), anti-α-tubulin (Santa Cruz Biotechnology, sc-53646, mouse monoclonal, dilution 1:500) anti-β-actin (Santa Cruz Biotechnology, sc-130656, rabbit polyclonal, dilution 1:500), HSP90 (Santa Cruz Biotechnology, sc-69703, mouse monoclonal, dilution 1:200), anti-CDK2 (Santa Cruz Biotechnology, sc-163, rabbit polyclonal, dilution 1:1,000), anti-BID (Cell Signaling Technology, 2002S, rabbit polyclonal, dilution 1:250), anti-MARCKS (Cell Signaling Technology, D88D11, rabbit monoclonal, dilution 1:500), anti-IMPDH2 (Abcam, Ab131158, rabbit monoclonal, dilution 1:1,000), anti-PSMC1 (Atlas, HPA000872, rabbit polyclonal, dilution 1:750), anti-GSTO1/2 (Santa Cruz Biotechnology, sc-166040, mouse monoclonal, dilution 1:100), and anti-HCCS (Atlas, HPA002946, rabbit polyclonal, dilution 1:500)). Membranes were then washed 3 × TBST before being incubated with the appropriate secondary antibody in blocking solution with gentle agitation for 2 h at room temperature (Goat anti-Rabbit IgG-HRP secondary antibody (Invitrogen, dilution 1:5,000) and Goat anti-Mouse IgG-HRP (BD Pharminigen, dilution 1:10,000)). Membranes were then washed with 3 × TBST before being developed with Luminata Crescendo Western HRP substrate (Millipore) according to the supplier's protocol.

4.4 In-cell quantitative, competition-based assays for target identification by mass spectrometry

The following method was used for MS-based proteomic identification of targets of **2** competed against a concentration gradient of sulforaphane in live, intact MCF7 and MDA-MB-231 cells. All experimental samples were generated in duplicate. The amount of peptide injected onto the LC-MS/MS for affinity purified peptide digests was equivalent to 20-40 µg of the starting protein lysate.

4.4.1 Compound treatment of cells and cell lysis

MDA-MB-231 and MCF7 cells were grown in 10 cm plates under standard cell culture conditions. Cells were incubated with D,L-sulforaphane (0 µM, 5 µM, 25 µM or 100 µM) in cell media (0.2% DMSO final) for 30 min. The media was aspirated and replaced with fresh media (0.2% DMSO final) containing D,L-sulforaphane and **2** (5 µM) for 30 min. The media was aspirated and the cells washed three times with PBS. Cells were then lysed using a fractionation protocol as described. Cells were lifted from the plate into PBS using a cell scraper and transferred to low protein binding tubes (ProteinLoBind tubes, Eppendorf). Cells were pelleted by centrifugation (2000 × g, 5 min). The supernatant was discarded and the cell pellet re-suspended in Buffer A (400 µL, 5 mM KCl, 0.5 mM $MgCl_2$, 0.5% NP-40, 25 mM HEPES pH 7.9, 1 × Complete EDTA-free protease inhibitor) and shaken at 4 ˚C for 15 min. Samples were then centrifuged (600 × g, 2 min) and the supernatant (cytosolic fraction) transferred to a new tube. The pellet was then washed with Buffer A (100 µL) then incubated with Buffer B (200 µL, 350 mM NaCl, 10% sucrose, 25 mM HEPES pH 7.9, 1 × Complete

EDTA-free protease inhibitor) for 1 h at 4 ˚C. The sample was then centrifuged (17,000 × g, 10 min, 4 ˚C) and the supernatant (nuclear fraction) transferred to a new tube. Protein concentration was determined for the cytosolic and nuclear fraction for each sample.

4.4.2 Preparation of ‘spike-in’ SILAC cell lysates as quantification standards

To generate ‘spike-in’ SILAC ‘heavy’ lysates, 8 × 10 cm plates of MDA-MB-231 cells and 9 × 10 cm plates of MCF7 cells previously established with R10K8 labelled proteomes were grown in ‘heavy’ media to 90% confluence. These cells were then treated with **2** (20 µM) for 30 min in ‘heavy’ media (0.2% DMSO final). Media was aspirated and cells washed three times with PBS prior to lysis as described previously to generate cytosolic and nuclear fractions. Lysates from the two fractions were pooled into a single master ‘spike-in’ SILAC ‘heavy’ lysate for each of the cytosolic and nuclear fractions for which the protein concentration was determined. A total protein amount for the master ‘spike-in’ SILAC lysate of 1.8 mg (nuclear) and 8.2 mg (cytosolic) for the MCF7 cell line and 1.6 mg (nuclear) and 6.4 mg (cytosolic) for the MDA-MB-231 cell line were generated.

4.4.3 CuAAC, affinity enrichment and on-bead reduction, alkylation and trypsin digest

Cytosolic fraction lysates (300 µL, 2 mg/mL) had added to them cytosolic master ‘spike-in’ SILAC lysate (100 µL, 2 mg/mL). Nuclear fraction lysates (90 µL, 1 mg/mL) had added to them nuclear master ‘spike-in’ SILAC lysate (30 µL, 1 mg/mL). Lysates were then subjected to CuAAC functionalisation with click reaction master mix containing azide capture reagent (24 µL for cytosolic lysates, 7.2 µL for nuclear lysates). Lysates were left to shake at room temperature for 1 h before being quenched and the protein precipitated.

The protein pellet was then re-suspended in 2% SDS in PBS (80 µL), 100 mM EDTA (80 µL), 100 mM DTT (8 µL), 1 × Complete EDTA-free protease inhibitor, and PBS (507 µL) (final volume 675 µL) for the cytosolic samples, and in 2% SDS in PBS (12 µL), 100 mM EDTA (12 µL), 100 mM DTT (1.2 µL), 1 × Complete EDTA-free protease inhibitor, and PBS (74.8 µL) (final volume 100 µL) for the nuclear samples. Samples were centrifuged (17,000 × g, 3 min) after re-suspension and transferred to new tubes. Neutravidin sepharose resin was washed with 3 × 0.2% SDS in PBS and to cytosolic samples resin slurry (125 µL) was added (800 µL volume, protein concentration 1 mg/mL, 0.2% SDS final), and to nuclear samples resin slurry (20 µL) was added (120 µL volume, protein concentration 1 mg/mL, 0.2% SDS final). Samples were shaken at room temperature for 2 h, the supernatant discarded and the Neutravidin sepharose resin washed 3 × 1% SDS in PBS, 2 × 4 M urea in 50 mM ammonium bicarbonate (AMBIC) and 4 × 50 mM AMBIC (5 vol. for each wash consisting of 2 min vortexing followed by brief centrifugation to pellet the resin and discard washings).

After washing, proteins on the Neutravidin sepharose resin were reduced with 100 mM DTT in 50 mM AMBIC (4 µL for cytosolic samples, 1.2 µL for nuclear samples) at 55 ˚C for 30 min with gentle agitation. The resin was washed 2 × 50 mM AMBIC. Cysteines were then alkylated with 100 mM iodoacetamide in 50 mM AMBIC (4 µL for cytosolic samples, 1.2 µL for nuclear samples) in the dark at room temperature. The resin was then washed 2 × 50 mM AMBIC. Samples were then digested with Trypsin (Sequencing Grade Modified Trypsin (Promega), 0.5 µg for cytosolic samples, 0.25 µg for nuclear samples) at 37 ˚C overnight with gentle agitation. Samples were then centrifuged and the supernatant transferred to a new tube. The resin was washed with 0.1% formic acid in water and centrifuged and this

supernatant added to the same tube. The peptide solutions were then stage-tipped according to a published protocol.[6] Briefly, stage tips were prepared by fitting C18 Empore disks (SDC-XC, 3M) into 200 µL pipette tips. The stage tip was initially washed by centrifuging (2000 × g, 2 min) with MeOH (150 µL) followed by water (150 µL). Peptide solutions were then added to the top of the stage tip and centrifuged (2000 × g, 2 min) to load the peptides onto the C18 sorbent followed by desalting by washing with water (150 µL). Peptides were eluted with 79% acetonitrile in water and dried with speed-vac-assisted solvent removal. Peptides were then re-dissolved in 0.5% TFA, 2% acetonitrile in water and transferred to LC-MS sample vials for LC-MS/MS analysis.

4.4.4 LC-MS/MS

LC-MS/MS analysis was performed on an Easy nLC-1000 system coupled to a Q Exactive mass spectrometer via an easy-spray source (Thermo Fisher Scientific). Tryptic peptide samples were separated with a reverse phase Acclaim PepMap RSLC column 50 cm × 75 µm inner diameter (Thermo Fisher Scientific) using a 2 h acetonitrile gradient in 0.1% formic acid at a flow rate of 250 nL/min. The Q Exactive mass spectrometer was operated in data-dependent mode with survey scans acquired at a resolution of 75,000 at *m/z* 200 (transient time 256 ms). Up to the top 10 most abundant isotope patterns with charge +2 from the survey scan were selected with an isolation window of 3.0 *m/z* and fragmented by HCD with normalized collision energies of 25 W. The maximum ion injection times for the survey scan and the MS/MS scans (acquired with a resolution of 17,500 at *m/z* 200) were 250 and 80 ms, respectively. The ion target value for MS was set to 10^6 and for MS/MS to 10^5.

4.4.5 LC-MS/MS data analysis

The mass spectrometry proteomics data have been deposited to the ProteomeXchange Consortium via the PRIDE[7] partner repository with the dataset identifier PXD006279. The .raw data file obtained from each LC-MS/MS acquisition was directly processed with the software MaxQuant version 1.3.0.5,[8] with the peptides being identified from the MS/MS spectra searched against the human UniProt+isoforms database (January 2014) using the Andromeda search engine. Cysteine carbamidomethylation (+57.021 Da) was set as a fixed modification and methionine oxidation (+15.995 Da) and N-terminal acetylation (+42.011 Da) set as variable modifications for the search. The multiplicity was set to 2 corresponding to the number of labels to quantify against one another (Lys0, Lys8, Arg0 and Arg10). The minimum length of a peptide was set to 7 residues, the maximum amount of missed trypsin cleavages was set to 2, the maximum number of modifications per peptide was set to 5 and the maximum charge of a peptide as +7. Peptide and protein FDRs were set to 0.01. Quantification of peptides was allowed off 'razor+unique' peptides carrying no modifications as well as methionine oxidation, N-terminal acetylation or carbamidomethylation. All other parameters were used as pre-set by the software.

Data outputted from MaxQuant was analysed using a combination of Perseus version 1.4.0.20, Microsoft Office Excel 2010 and GraphPad Prism 5.0. The corresponding data files from the two fractions (cytosolic and nuclear) of the same sample were combined to form a single file. Protein identifications by MaxQuant based on 'contaminants', 'only identified by site' and 'reverse' were filtered out. Further filtering only allowed identification of a protein target if it contained at least two 'razor+unique peptides' in both biological duplicates for each differing experimental condition. A two sample t-test (S0 = 1, FDR = 0.01) was carried out across the replicates on the original H/L ratios between the **2** only sample and the **2** plus

sulforaphane competition samples to identify statistically significant differences. Medium-confidence targets were defined as those giving a statistically significant difference at any concentration of sulforaphane competition. High-confidence targets were defined as medium-confidence targets present at 5, 25 and 100 μM, or 25 and 100 μM sulforaphane competition.

The averaged H/L ratios for each concentration of competition against sulforaphane (5 μM, 25 μM and 100 μM) were normalised to the H/L ratio of the **2** only sample to generate a QS for each concentration of competition against sulforaphane. To visualise competition across the sulforaphane concentration gradient against **2**, heat maps were generated in Perseus. The colour coding for the $\log_2$(quantification score) was defined as: Blue – 0, Green – 1.0, Yellow – 2.0, Red – 3.0, Grey – no value calculated. The quantification score for the three sulforaphane competition concentrations (5 μM, 25 μM and 100 μM) from the MCF7 and MDA-MB-231 cell line for a select number of targets were then plotted against one another as depicted in Fig. 3. Where a protein was only present in one cell line, an arbitrary value of 0 was assigned as the quantification score for that protein ID for the purpose of the plot.

4.4.6 Abundance and bioinformatic analysis

High-confidence target abundances were taken from iBAQ values for each cell line from proteomic analysis of the NCI-60 cell line panel.[1] To allow analysis of proteins that were not detected in a given cell line (iBAQ = 0), relative abundances were calculated using the following formula:

$$Relative\ Abundance = \frac{Abundance_{MCF7} - Abundance_{average}}{Abundance_{average}}$$

Targets that were not detected in MCF7 or MDA-MB-231 cell lines would give values of -1 or 1, respectively. Targets that were not detected in iBAQ measurements in either cell line were excluded from analysis.

High confidence targets of sulforaphane were analysed using Ingenuity® Pathway Analysis (Qiagen), inputting $-\log_2$(QS) at each concentration to represent downregulation of protein function upon sulforaphane binding.

5 References

1 A. M. Gholami, H. Hahne, Z. Wu, F. J. Auer, C. Meng, M. Wilhelm and B. Kuster, *Cell Rep.*, 2013, **4**, 609–620.
2 W. P. Heal, M. H. Wright, E. Thinon and E. W. Tate, *Nat. Protoc.*, 2012, **7**, 105–117.
3 E. Thinon, R. A. Serwa, M. Broncel, J. A. Brannigan, U. Brassat, M. H. Wright, W. P. Heal, A. J. Wilkinson, D. J. Mann and E. W. Tate, *Nat. Commun.*, 2014, **5**, 4919.
4 M. Broncel, R. A. Serwa, P. Ciepla, E. Krause, M. J. Dallman, A. I. Magee and E. W. Tate, *Angew. Chem. Int. Ed Engl.*, 2015, **54**, 5948–5951.
5 S.-E. Ong and M. Mann, *Nat. Protoc.*, 2007, **1**, 2650–2660.
6 J. Rappsilber, Y. Ishihama and M. Mann, *Anal. Chem.*, 2003, **75**, 663–670.
7 J. A. Vizcaíno, A. Csordas, N. del-Toro, J. A. Dianes, J. Griss, I. Lavidas, G. Mayer, Y. Perez-Riverol, F. Reisinger, T. Ternent, Q.-W. Xu, R. Wang and H. Hermjakob, *Nucleic Acids Res.*, 2016, **44**, D447–D456.
8 J. Cox and M. Mann, *Nat. Biotechnol.*, 2008, **26**, 1367–1372.
